# Exploring the Nonenzymatic Origin of Duclauxin-like Natural Products

**DOI:** 10.1021/acs.jnatprod.4c00558

**Published:** 2024-09-10

**Authors:** Enrique Aguilar-Ramírez, José Rivera-Chávez, Brandon D. Alvarado-Zacarías, José E. Barquera-Lozada

**Affiliations:** † Department of Natural Products, Institute of Chemistry, Universidad Nacional Autónoma de México, Mexico City, 04510, México; § Department of Physical Chemistry, Institute of Chemistry, Universidad Nacional Autónoma de México, Mexico City, 04510, México

## Abstract

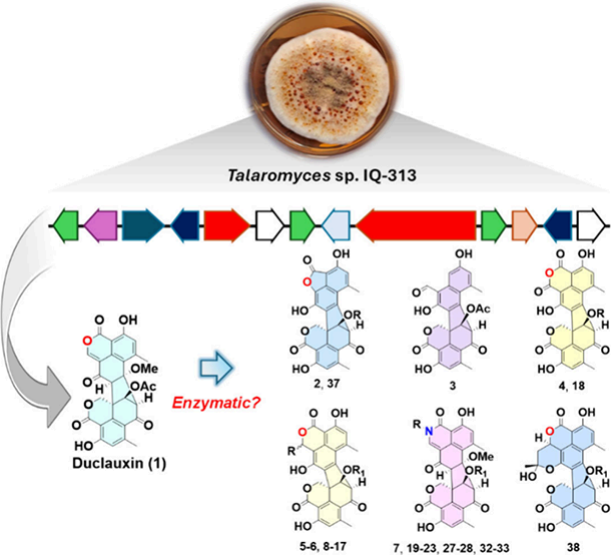

Chemical-biological efforts to increase the diversity of duclauxin (**1**)-like molecules for medicinal chemistry purposes unveiled the reactivity of duclauxin (**1**) toward amines and alcohols. To expand the compound class, a semisynthetic strategy conjugating amines to duclauxin (**1**) was employed. Insights gained from this approach led to the hypothesis that certain duclauxin-like “natural products” such as talaromycesone B (**2**), bacillisporin G (**3**), xenoclauxin (**4**), bacillisporins F (**5**/**6**), bacillisporins J (**8**/**9**), bacillisporins I (**12**/**13**), and verruculosin A (**38**) may be isolation artifacts rather than enzymatic products. Further experimentation, involving adsorption of **1** onto silica gel, resulted in the production of **2**–**6**. To gain insights into the conditions that generate such molecules, one-step reactions under mild conditions were set. Outcomes from both experiments confirmed that duclauxin-like molecules are generated via nonenzymatic reactions. This article presents analytical evidence, indicating that these molecules originate from **1**, with the epimeric mixture of bacillisporins J (**8** and **9**) acting as the primary intermediate.

Fungal natural products are commonly perceived as the result of biosynthetic processes driven by enzymes encoded by a group of genes concatenated together, referred to as biosynthetic gene clusters (BCGs).^1^ However, recent investigations have revealed that in some cases the inherent chemical reactivity of metabolic intermediates and substrates escapes the contribution of enzymes.^2^ Despite considerable progress in elucidating biosynthetic pathways, challenges persist, particularly in unraveling the roles of nonenzymatic reactions in product diversification.^2,3^

Nonenzymatic reactions (intramolecular reactions, homologous multicomponent reactions, heterologous multicomponent reactions, tailoring reactions, and light-induced reactions) can be divided into two main groups: (i) those that are functional in the context of an organism, and (ii) those that occur spontaneously as isolation artifacts.^2^ Several examples of these kinds of modifications have been reported in the literature, for example elansolid A3,^4^ torreyanic acid,^5^ rubrolones,^6^ ammosamides,^7^ brevinamides,^10^ and glyclauxins (duclauxin analogues conjugated with aminoglycosides),^8^ among several others.^2^

Duclauxin (**1**) is a dimeric heptaketide synthesized by a nonreducing polyketide synthase (NR-PKS), composed of an isocoumarin and a dihydroisocoumarin unit.^9,10^ Isolated from a strain of *Penicillium duclauxii* cultivated on Czapek-Dox medium in 1965,^11^**1** has captivated the interest of natural products chemists for years. This attention is likely attributed to its intriguing scaffold, which is structured as a heptacyclic ring system. This distinctive structure is derived from the extensive redox modifications of phenalenone to generate the dihydroisocoumarin SF226,^12^ which undergoes subsequent modifications to generate C-9a′ and C-8 radicals, that react to form the C-9a′–C-8 bond, resulting in the formation of a heterodimer. Following this, an aldol-like condensation gives rise to duclauxin-scaffold, which subsequently undertakes additional late-stage redox modifications to generate **1** (Scheme 1).^13^

**Scheme 1:**
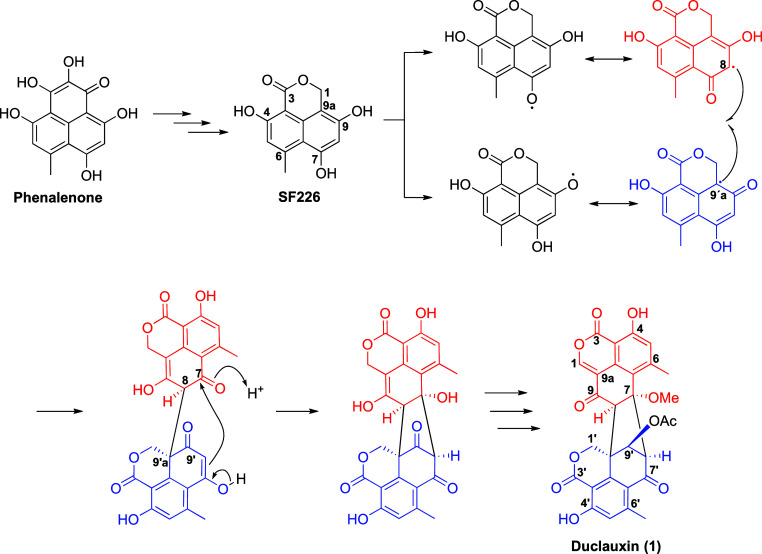
Proposed Biosynthesis for Duclauxin (**1**)

Aside from duclauxin (**1**), currently, more than 50 bioactive analogues of **1** (Figure S1) have been isolated and reported as natural products from many species of *Penicillium* and *Talaromyces*.^14^ Presumably, the great diversity of this class of “natural products” arises from (i) the ability of the enzymatic machinery of this genus to perform homo- and heterodimeric couplings among SF226 (**3**) and several phenalenone-like units;^13^ (ii) additional late-stage redox modifications of the duclauxin-scaffold;^13^ (iii) the reactivity of the isocoumarin moiety in **1**, which can couple to primary amines to generate amido-derivatives;^8,15,16^ (iv) the [4 + 2] cycloadditions between the heterodiene in **1** (C-1 to C-9 α,β-unsaturated carbonyl) with olefinic functionalities;^17,18^ and (v) the nucleophilic addition of exocyclic or endocyclic-enols to the electrophilic isocoumarin of **1**.^17,18^

Interestingly, from the great diversity of duclauxin-like derivatives, some are products of the C-9′ acetate hydrolysis and/or the C-7 MeOH elimination reactions, as well as the addition of alcohols to C-1 in **1** to generate epimeric acetals or hemiacetals, products of decarboxylation, deformylation, and oxidation reactions. Given these observations, it is plausible to hypothesize that some of the reported duclauxin-like natural products may, in fact, be products generated through the isolation process (artifacts).

## Results and Discussion

As part of an ongoing project aimed at identifying allosteric inhibitors of full-length human PTP1B (*h*PTP1B_1–400_), the extract of *Talaromyces* sp. IQ-313 was investigated. Through chromatographic fractionation, duclauxin (**1**), talaromycesone B (**2**),^19^ bacillisporin G (**3**),^20^ and xenoclauxin (**4**)^21^ were isolated.^22^ All these compounds inhibited the enzymatic activity of *h*PTP1B_1–400_ in a concentration-dependent fashion, with IC_50_ values ranging from 12 to 90 μM.^22^ Further exploration of the fungal strain’s potential to produce duclauxin (**1**) analogues led to the isolation of bacillisporins F (**5** and **6**) and bacillisporin H (**7**) through cocultivation experiments with fungal (*Aspergillus* sp. IQ-045) and bacterial (*Klebsiella pneumoniae*) strains, respectively. However, this approach did not yield new derivatives of **1**.^16^ To address this limitation, and building on previous findings, a one-step semisynthetic strategy to conjugate amines to the core structure of **1** was applied, thereby expanding the chemical diversity of this compound class.^16^ During this process, two noteworthy observations were made: (i) the transformation of **1** into **2** under the presence of diffrent amnines and DMSO and (ii) the elimination of the MeOH moiety at C-7, resulting in the formation of a new double bond between C-7 and C-8. These observations suggest that some duclauxin-like natural products reported in the literature may actually be artifacts of the isolation process rather than enzymatic products, as previously presumed.^13^

**Figure 1:**
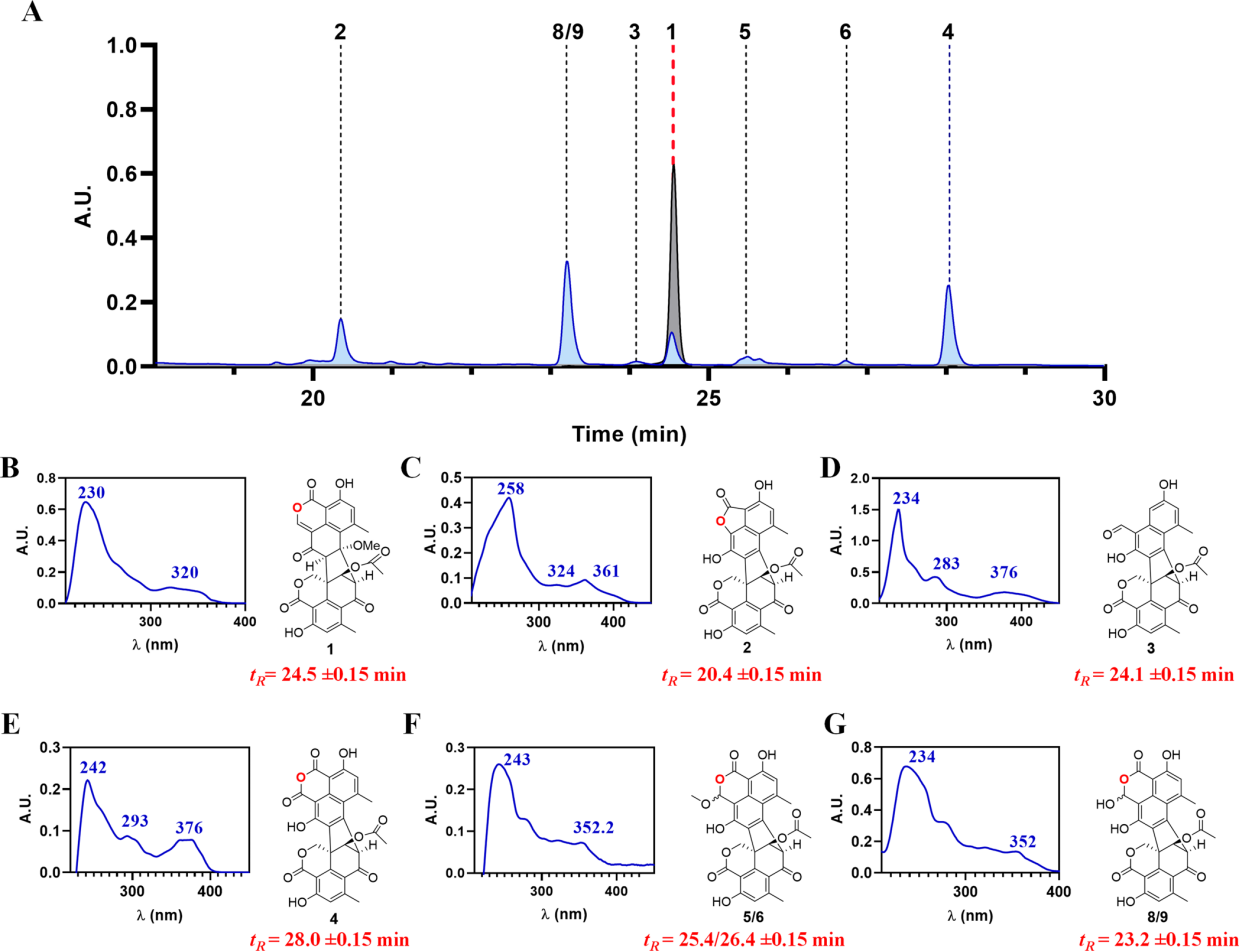
A) In gray HPLC profile of pure duclauxin (**1**). In blue HPLC profile of recovered material obtained after extracting (CH_3_CN) **1** adsorbed onto silica gel over 20 days. B) UV profile of **1**. C) UV profile of talaromycesone B (**2**). D) UV profile of bacillisporin G (**3**). E) UV profile of xenoclauxin (**4**). F) UV profile of bacillisporin F and its 1-epimer (**5/6**). G) UV profile of bacillisporin J and its 1-epimer (**8/9**).

Based upon these observations, it was hypothesized that **2**–**6**, **8**, and **9** the most isolated duclauxin-like molecules are not naturally occurring compounds. Instead, they are believed to be byproducts of **1**, produced during the isolation process. To test this premise, duclauxin (**1**) was adsorbed onto silica gel for a period of 20 days, followed by extraction with CH_3_CN. HPLC analysis (Figure 1) of the recovered material confirmed the conversion of **1** to the anticipated products. The identity of all molecules was confirmed by comparison of their UV-profile, retention time (*t*_R_) (Figure S2) and 1D-NMR spectroscopic data (Supporting Information).

**Scheme 2:**
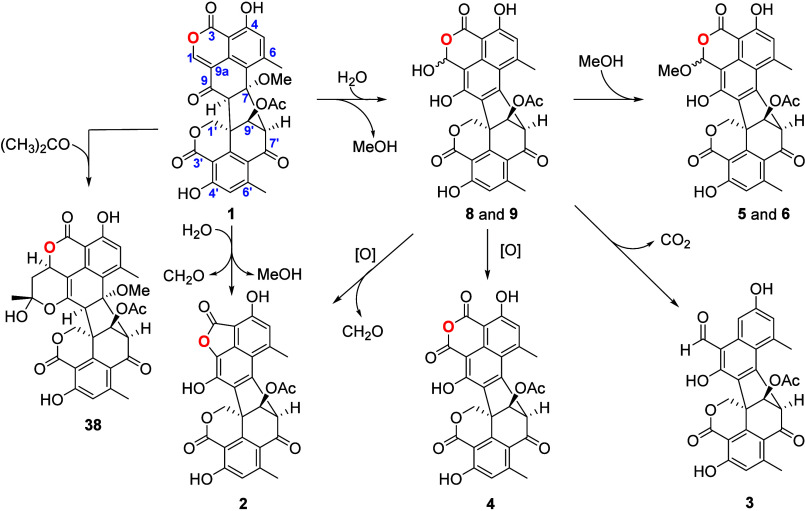
Transformation of Duclauxin (**1**) into Talaromycesone B (**2**), Bacillisporin G (**3**), Xenoclauxin (**4**), Bacillisporins F and J and Their Epimers (**5**, **6**, **8**, and **9**)

To further investigate the reaction conditions responsible for producing these molecules and considering the significant proficiency of *Talaromyces* sp. IQ-313 in production of **1** (up to 0.6 g/10 g substrate), this molecule was exposed to mild reaction conditions using wet DMSO, MeOH, or CH_2_Cl_2_ as solvents. Remarkably, these experiments yielded talaromycesone B (**2**), bacillisporin G (**3**), xenoclauxin (**4**), and bacillisporins F (**5**/**6**) and J (**8**/**9**), alongside other derivatives. In this paper, we present analytical evidence indicating that these molecules likely originated from **1**, with the epimeric mixture of **8** and **9** being identified as the primary intermediate (Scheme 2) in all cases.

### Transformation of Duclauxin (**1**) into Bacillisporins J (**8** and **9**)

**Figure 2:**
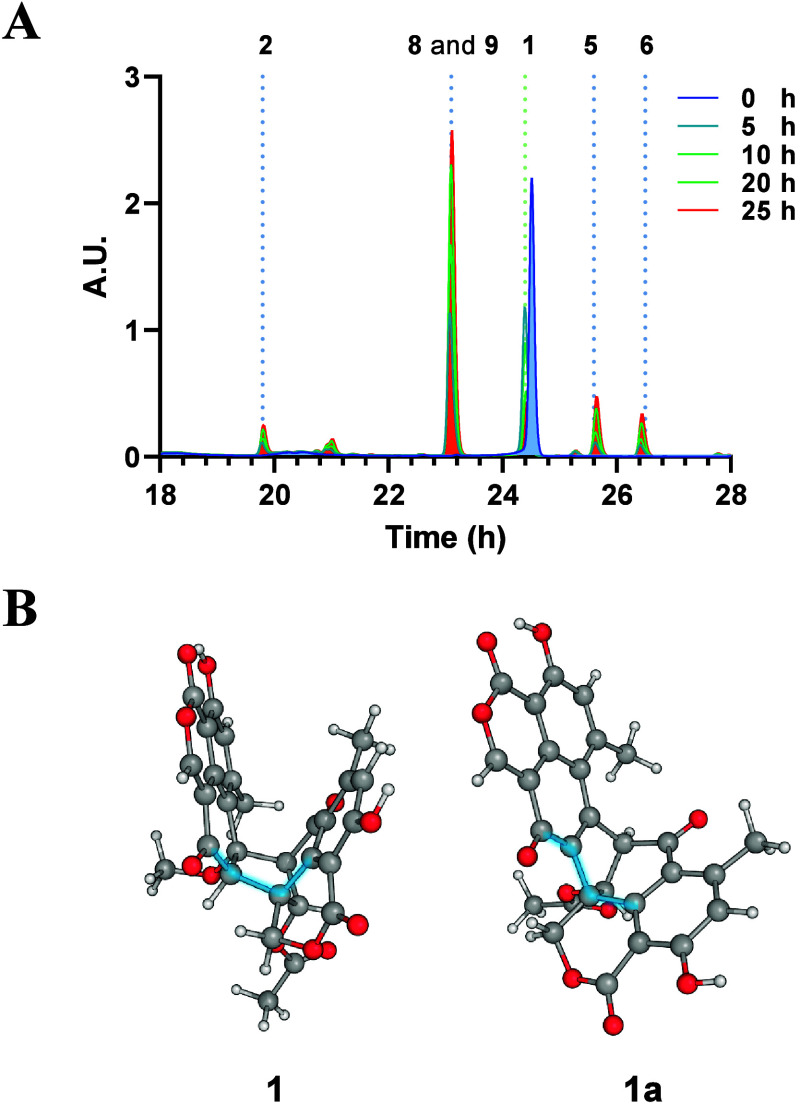
A) Kinetics for the transformation of **1** into **8** and **9**. Through time small amounts of bacillisporins F (**5** and **6**) and talaromycesone B (**2**) are also generated, while the concentration of duclauxin (**1**) decreases. B) Optimized structures for compounds **1** and **1a**, showing the conformational change from U to Z-shape, respectively. The blue bonds indicate significant conformational changes caused by E1cB-MeOH elimination.^16^

**Scheme 3:**
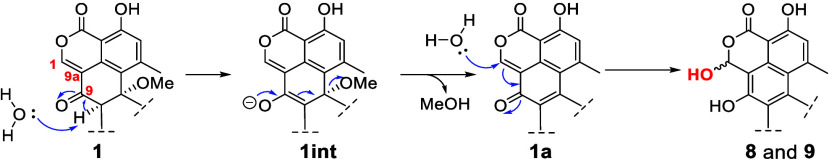
Proposed Mechanism for the Transformation of Duclauxin (**1**) into Bacillisporins J (**8** and **9**)

Bacillisporin J (**8**) and its 1-epimer (**9**) were semisynthesized by reacting **1** with H_2_O in DMSO, with continuous stirring for 24 h (Figure 2A), following the proposed mechanism outlined in Scheme 3. This pathway suggests that **1** undergoes an E1cB-MeOH elimination^23−25^ to produce **1a**. This transformation induces substantial conformational changes, transitioning from a U shape in **1** to a Z shape in **1a** (Figure 2B).^16^ The altered conformation facilitates a 1:1 *Re*, *Si*, 1,4-addition of H_2_O to the C-1 to C-9 α,β-unsaturated ketone, eventually leading to the formation of bacillisporin J and its 1-epimer (**8**/**9**).^23^ Compound **1** satisfies classical structural prerequisites for the ElcB mechanism. It possesses an acidic hydrogen atom (H-8, adjacent to the ketone at C-9), has the potential to stabilize a negative charge by electron withdrawing, and contains a poor leaving group (MeO^–^), and both the H-8 and MeO– are *syn* oriented.^25,26^ Interestingly, the addition of triethylamine (Et_3_N) to the mixture accelerated the reaction, suggesting that the β-elimination of methoxide from **1** is a base catalyzed reaction. Additionally, the formation of a fully conjugated system acting as the driving force for this reaction supports the E1cB elimination.

### Transformation of Bacillisporins J (**8** and **9**) into Bacillisporins F (**5** and **6**)

**Figure 3:**
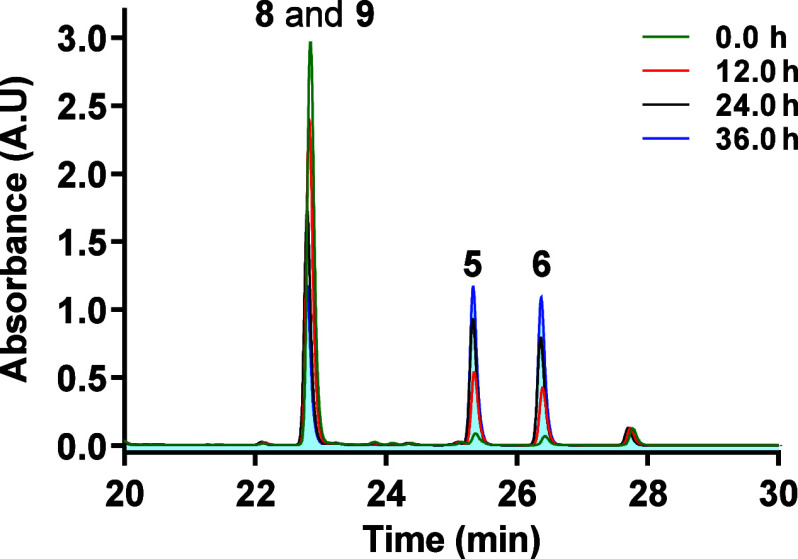
Kinetics for the transformation of **8**/**9** into **5**/**6**.

**Scheme 4:**
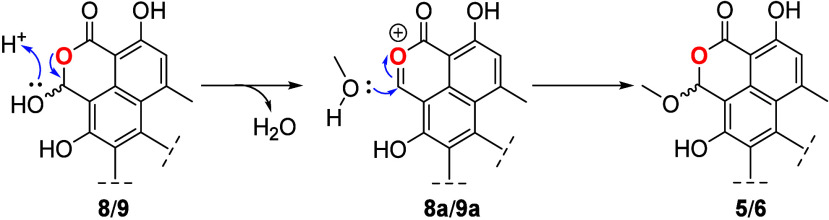
Proposed Mechanism for the Transformation of Bacillisporins J (**8** and **9**) into Bacillisporins F (**5** and **6**)

Transformation of bacillisporin J (**8** and **9**) into bacillisporins F (**5** and **6**) occurs spontaneously as indicated by DFT calculations, predicting a *ΔG*_r_ of −2.3 kcal/mol. According to kinetics studies, this reaction generates an ∼1:1 mixture of the 1*S* and 1*R* epimers of bacillisporin F (Figure 3), when dissolved in MeOH. This process follows the typical acetal reaction formation from hemiacetals, proceeding through the mechanism outlined in Scheme 4. In this scheme, the 1-OH group protonates, leading to H_2_O elimination and formation of intermediate **8a**/**9a**, which underwent a MeOH addition reaction, followed by a subsequent deprotonation step.^27^ Beyond **5** and **6**, this reaction could lead to obtain acetal-derivatives of **1** with other alcohols. In this study, the new bacillisporin M (**10**) and its 1-epimer (**11**) were semisynthesized by reacting slightly acidic isopropanol with the epimeric mixture of **8**/**9**.

### Hydrolysis of Bacillisporins J (**8/9**) into Bacillisporins I (**12/13**)

The C-1 epimeric mixture of bacillisporins I- (C-9′ desacetylated bacillisporin J) was generated via a hydrolysis reaction by exposing the 1*R* and 1*S* diastereomeric mixture of **8**/**9** to 0.25 N aqueous HCl, suggesting that the C-9′ deacetylated duclauxin-like natural products must be generated during the chromatographic processes, especially if acidic silica is used as a stationary phase, generating appropriate conditions.

Together, these two reactions could originate a series of duclauxin acetals bearing an acetate (bacillisporin F (**5**),^20^ 1-*epi*-bacillisporin F (**6**),^20^ bacillisporin J (**8**),^28^ 1-*epi*-bacillisporin J (**9**),^28^ bacillisporin M (**10**), and 1-*epi*-bacillisporin M (**11**)) or an alcohol moiety at C-9′ (bacillisporin I (**12**),^28^ 1-*epi*-bacillisporin I (**13**),^28^ bacillisporin K (**14**),^29^ bacillisporin L (**15**),^29^ macrospurosone E (**16**),^30^ and 1-*epi*-macrospurosone E (**17**)).^30^
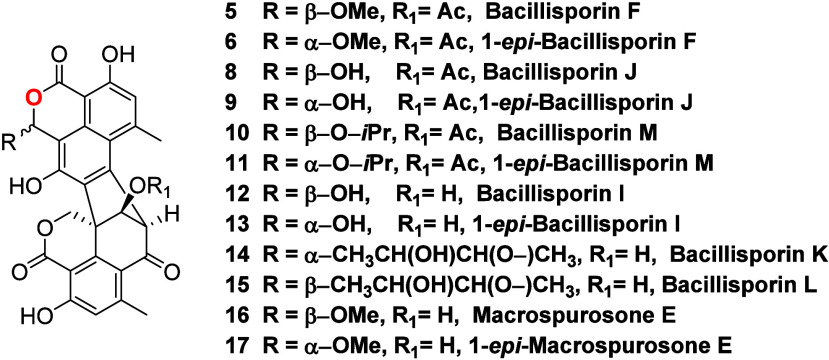


### Transformation of Bacillisporins J (**8/9**) into Bacillisporin G (**3**)

**Scheme 5:**
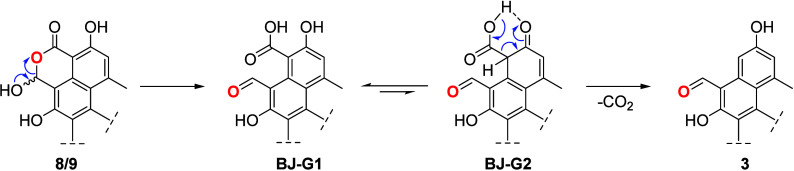
Proposed Mechanism for the Transformation of Bacillisporins J (**8** and **9**) into Bacillisporin G (**3**)

Compound **3** was obtained after recovering **1** and **8**/**9** adsorbed onto silica gel for 5 days. The presence of bacillisporin G (**3**) was confirmed by a comparison of the retention time and UV profile with those of an authentic reference. The proposed reaction mechanism (Scheme 5) for this transformation includes intermediate **BJ-G1** (phenolic acid), which tautomerizes to a β-keto acid, to generate a six-membered H-bonded system (intermediate **BJ-G2**). Following this, **BJ-G2** undergoes a decarboxylation reaction, through a cyclic, concerted transition state resulting in the formation of bacillisporin G (**3**).^31^ Interestingly, this reaction occurs at room temperature, in a similar way to decarboxylation of acetoacetic acid,^32^ and the conversion of ibotenic acid to muscimol in *Amanita muscaria*.^33^ Another example of natural products that undergo decarboxylation reactions are the cannabinoids synthesized by *Cannabis sativa*: Δ^9^-tetrahydrocannabinolic acid, cannabidiolic acid and cannabigerolic acid, which are unstable and can be decarboxylated when exposed to light or heat.^34^

### Transformation of Bacillisporin J (**8/9**) into Xenoclauxin (**4**)

**Figure 4:**
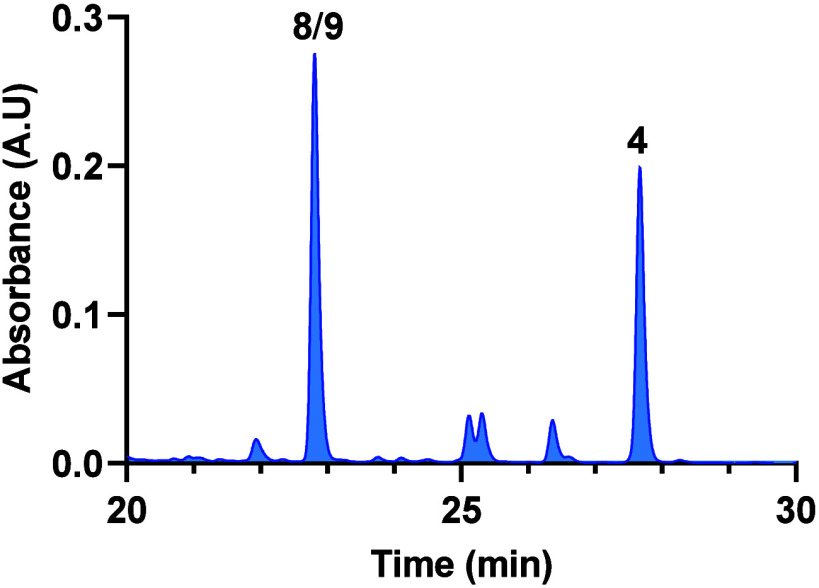
HPLC profile of the bacillisporin J epimeric mixture (**8**/**9**) after 5 days dissolved in a suspension of silica gel in CH_2_Cl_2_, revealing the formation of xenoclauxin (**4**).

**Scheme 6:**
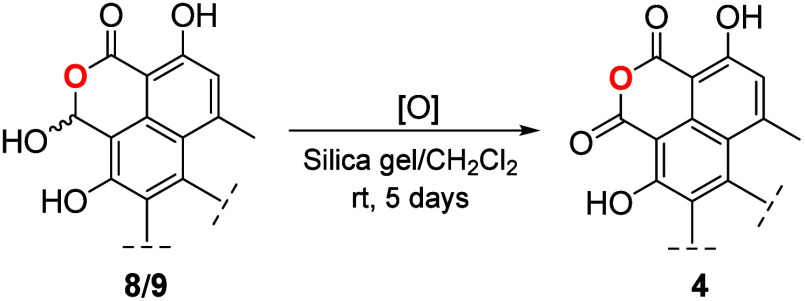
Reaction Conditions for the Transformation of Bacillisporins J (**8** and **9**) into Xenoclauxin (**4**)

Xenoclauxin (**4**) was initially obtained from the acetone extract of *Penicillium duclauxii* and prepared from **1** via an oxidation reaction with chromic acid in a mixture of acetic-acid and pyridine.^21^ In this work, **4** was prepared by dissolving bacillisporin J (**8**/**9**) in a suspension of silica gel in CH_2_Cl_2_ (Figure 4, Scheme 6). It is likely that the transformation involves an oxidation process facilitated by molecular oxygen,^35,36^ with silica serving as a catalyst, activating the polar substrates by hydrogen bonding.^37^ SiO_2_ has been reported to catalyze the oxidation of benzoins to benzils under solvent-free conditions, but neither diphenyl carbinol nor benzyl alcohol reacted.^38^ Although silica gel is commonly employed in normal-phase chromatography in natural products research,^39^ there have been only a few reports of it acting as a catalyst in oxidation processes. For example, it has been reported that silica catalyzes the oxidation of some natural products, such as kemposide A to form an aldehyde derivative with a shortened carbon scaffold formed through the breakdown of the isopropenyl group, the oxidation of the double bond in the prenyl chain of 8-prenylapigenin that leads to the formation of 1″-hydroxyretamasin C and retamasin B,^40^ and the oxidative cleavage of the elemene-type diterpenoid fuscol.^41,42^

Likewise, transformation of xenoclauxin (**4**) into macrosporusone D (**18**)^30^ could be achieved by reacting the former with diluted HCl, as described for the conversion of **8**/**9** into **12**/**13**.
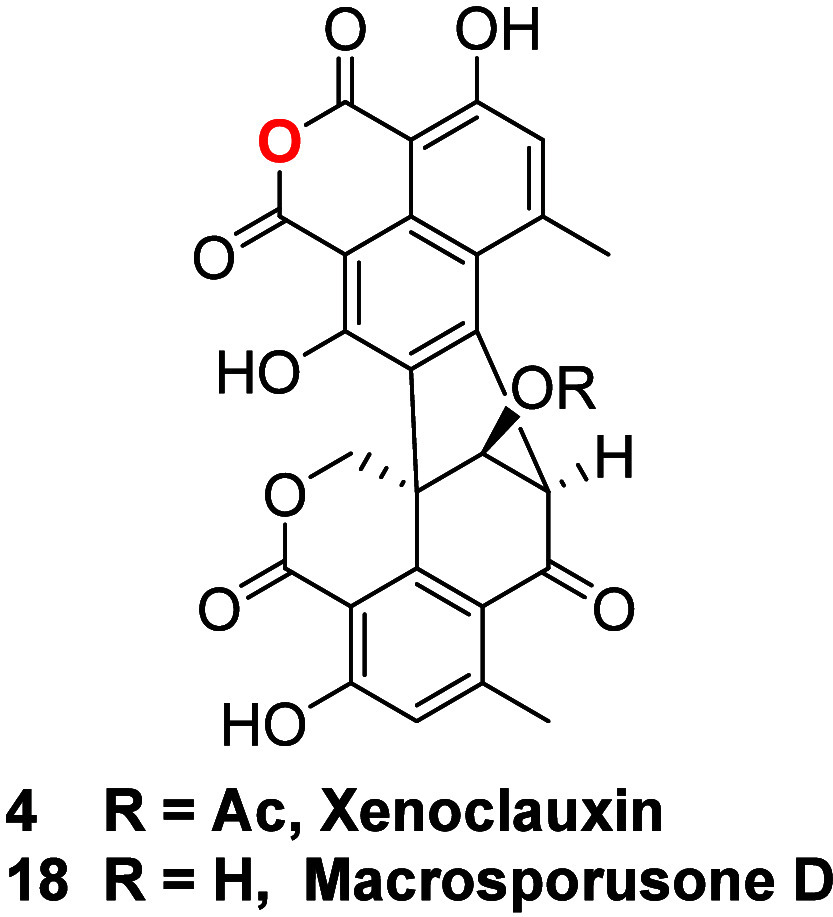


### Transformation of Duclauxin (**1**) into N-Containing Derivatives

**Scheme 7:**
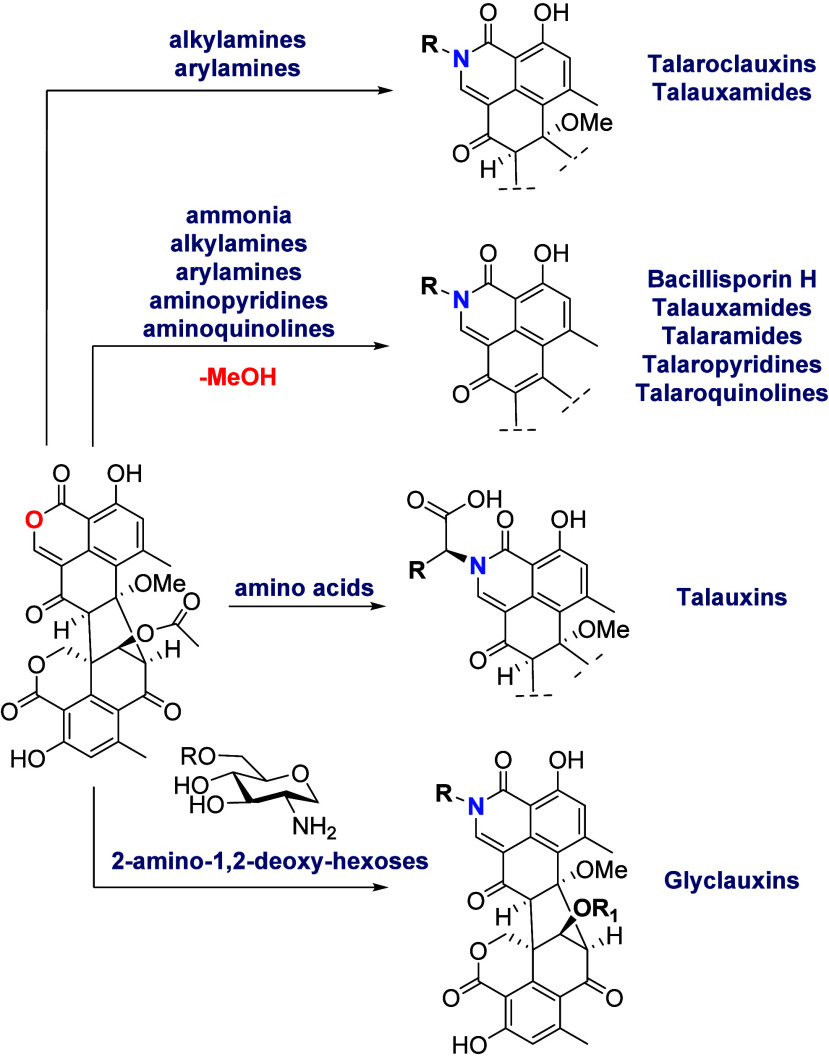
Plausible Synthetic Strategy to Access N-Containing Duclauxin Derivatives Having **1** as a Starting Material

The occurrence of N-containing duclauxin analogues has been well documented, with more than 15 derivatives isolated (bacillisporin H (**7**),^20^ talauxins (**19**–**23**),^15^ glyclauxins (**24**–**28**),^8^ duclauxamides (**29**–**31**),^28,43^ and talaroclauxins (**32**–**33**)).^17^ Although these molecules are considered to be specialized metabolites produced from conditional metabolic pathways, it is likely that such derivatives are in fact generated *in situ* without enzymatic participation, by combination of **1** and primary amines that are present in the cell or cellular environment, as previously demonstrated by Aguilar-Ramirez et al.^16^ who performed the partial synthesis of more than 30 N-containing duclauxin derivatives (talauxamides, talaramides, talaropyridines, and talaroquinolines), including **7** and some talauxins (Scheme 7).

A plausible mechanism to generate N-containing duclauxin derivatives involves an initial step of nucleophilic addition and subsequent ring opening of the lactone group (C-3), followed by a Schiff base recyclization process, accompanied by the release of H_2_O.^8,15^

**Chart 1:**
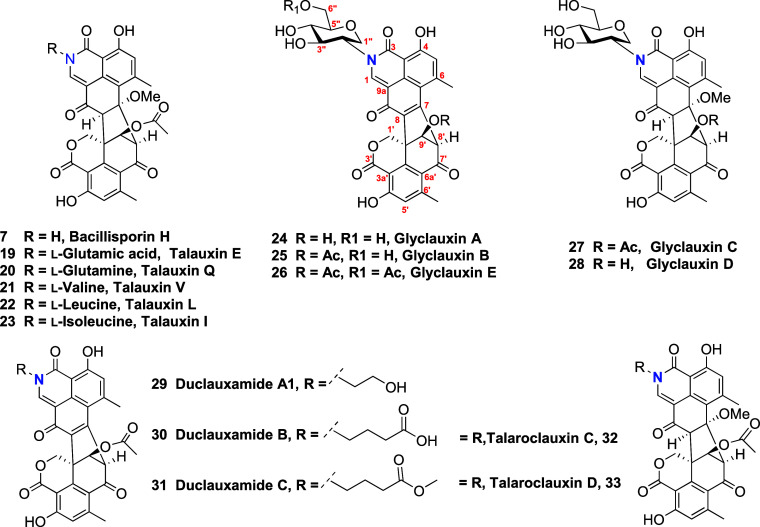


**Figure 5:**
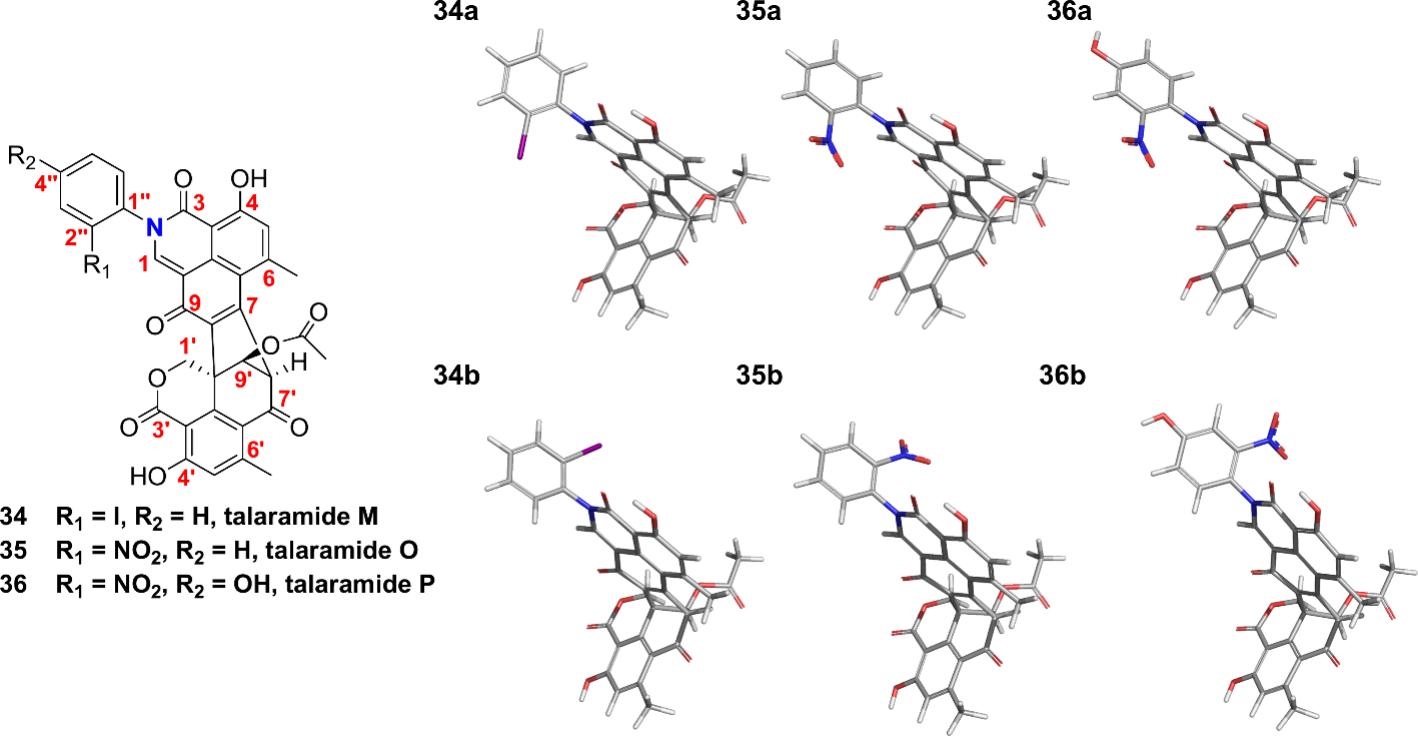
Structural models generated for the atropisomers of **35**–**37**. The structures were built using Spartan10 and geometrically optimized employing a semiempirical PM3 method. All molecules in series “a” have an axial configuration *R*a (*P*), while molecules in series “*b*” have the opposite configuration *S*a (*M*).

In the work by Samarasekera et al.^8^ an intriguing observation was noted: the absence of detectable NMR resonances for C-1, C-2′, and H-2′ (as well as C-3′ and H-3′) in certain glyclauxins. The authors attributed this phenomenon to NMR resonance broadening, which is linked to increased rotational freedom, resulting in the existence of multiple rotamers on the NMR time scale. Conversely to the observations made by Capon’s group, in recent work by our group it was observed the appearance of doubled ^1^H (1:1 to 3:2 proportion) and ^13^C NMR signals when coupling bulky-*ortho* substituted anilines, methyl-aminopyridines, or quinolines with duclauxin (**1**) for all protons of the corresponding substituent and hydrogens H-1′, H-5′, H-8′, H-9′, and 4′-OH. This evidence suggests that *ortho*-substituted anilines with bulky groups like iodine (talaramide M (**34**)), and -NO_2_ (talaramides O (**35**) and P (**36**))^16^ restrict the σ bond rotation between the aromatic ring and the N atom of the lactam. This feature induces epimerization due to conformational chirality or atropisomerism, altering the chemical and magnetic environments of hydrogen and carbon atoms. According to LaPlante’s classification (based on the half-lives of atropisomers - class 1 (*t*_1/2_ < 60 s), class 2 (60 s < *t*_1/2_ < 4.5 years), and class 3 (*t*_1/2_ > 4.5 years),^44,45^ product **34** is classified as class 2, as both conformers are distinguishable by HPLC (Figure S42). These results suggest that those substances were obtained as mixtures of axial isomers *R*a (*P*) and *S*a (*M*) (Figure 5).

### Transformation of Duclauxin (**1**) into Talaromycesone B (**2**)

**Figure 6:**
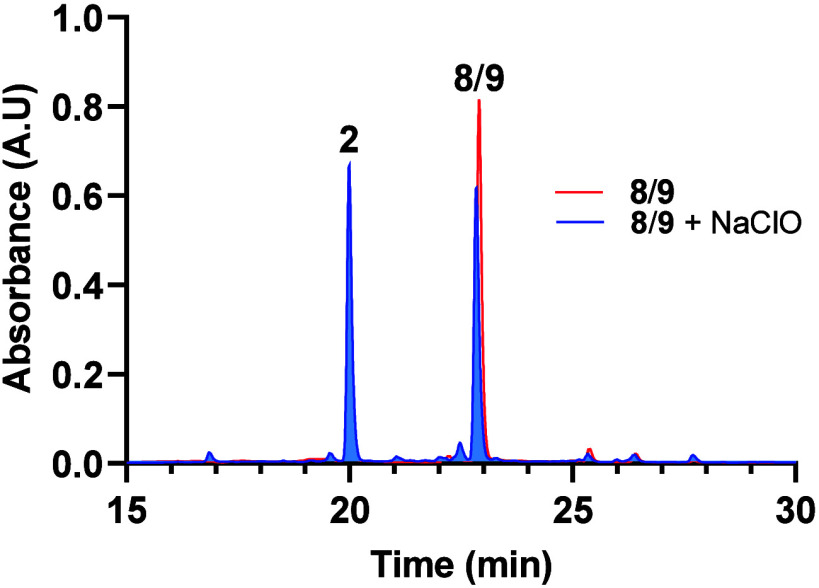
HPLC profile of bacillisporin J epimeric mixture (**8**/**9**, red line). In blue, HPLC profile of the reaction overnight (12 h) of bacillisporin J epimeric mixture (**8**/**9**) with NaClO to generate **2**.

Talaromycesone B (**2**)^19^ and C (**37**)^30^ represent the exclusive duo within duclauxin-like natural products featuring a unique 5/6/6/5/6/6/6 ring system as opposed to the more common 6/6/6/5/6/6/6 skeleton found in other dimeric phenalenones. This alteration in molecular architecture significantly influences their bioactivity as modulators of the full-length-human recombinant protein tyrosine phosphatase 1B (*h*PTP1B_1–400_).^16,22^ The complex structure of these specialized metabolites would suggest a natural origin. Surprisingly, exposing bacillisporin J (**8/9**) to an excess of base (diethylenetriamine) in DMSO yielded talaromycesone B (**2**) as evidenced by the retention time and UV profile of the products and confirmed by comparison of the ^1^H NMR data with an authentic reference sample. Also, compound **2** was obtained when **1** was reacted with different amines, by adding sodium hypochlorite to bacillisporin J (**8**/**9**) in DMSO (Figure 6), or by adsorbing **1** into silica gel for a few days. Based on the evidence presented, **2** could indeed be an artifact derived from **1** or **8**/**9**. This process involves the release of formaldehyde; nonetheless, the exact reaction mechanism remains unclear.

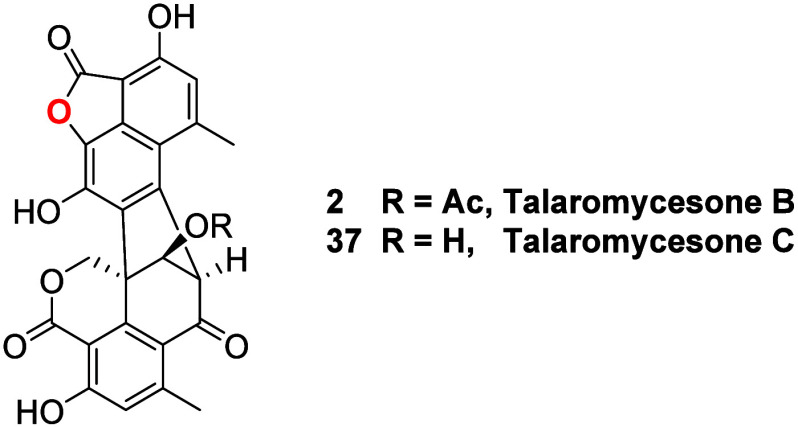



Transformation of **2** into talaromycesone C (**37**)^30^ could be achieved by reacting the former with diluted HCl, as described for the conversion of **8**/**9** into **12**/**13**. This product could also be accessed *via* basic hydrolysis using diethylenetriamine.

### Transformation of Duclauxin (**1**) into Verruculosin A (**38**)

**Scheme 8:**
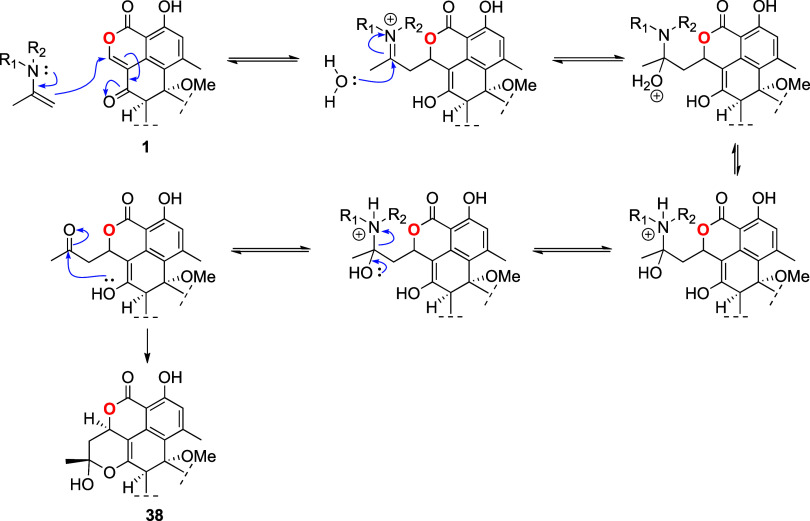
Proposed Mechanism for the Transformation of Duclauxin (**1**) into Verruculosin A (**38**)

Verruculosin A (**38**) is an oligophenalenone possessing an octacyclic scaffold. It was obtained from the acetone extract of *Talaromyces verruculosus*. Building upon the reactivity of the C-1 to C-9 α,β-unsaturated carbonyl segment of **1** and the plausibility of reacting with exocyclic or endocyclic-enols, it was hypothesized that **38** is in fact generated from **1** and acetone, *via* a mechanism (Scheme 8) involving the reaction of acetone with an amine (present in the intracellular environment) to generate an enamine (Scheme S1), which undergoes a Michael addition, hydrolysis, and cyclization.

**Scheme 9:**
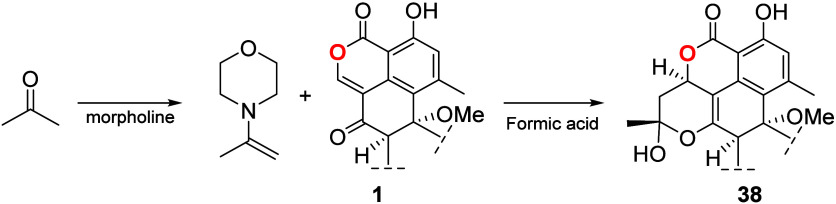
Reaction Conditions for the Transformation of Duclauxin (**1**) into Verruculosin A (**38**)

To test this hypothesis, acetone (100 μL) was mixed with morpholine (100 μL) to generate the corresponding enamine (4-(prop-1-en-2-yl)morpholine). Afterward, this mixture was added to a solution of 10 mg of **1** in acetone (300 μL), and the mixture was allowed to react under continuous stirring for 24 h at room temperature. Subsequently, to the reaction mixture was added 50 μL of formic acid (Scheme 9).

**Figure 7:**
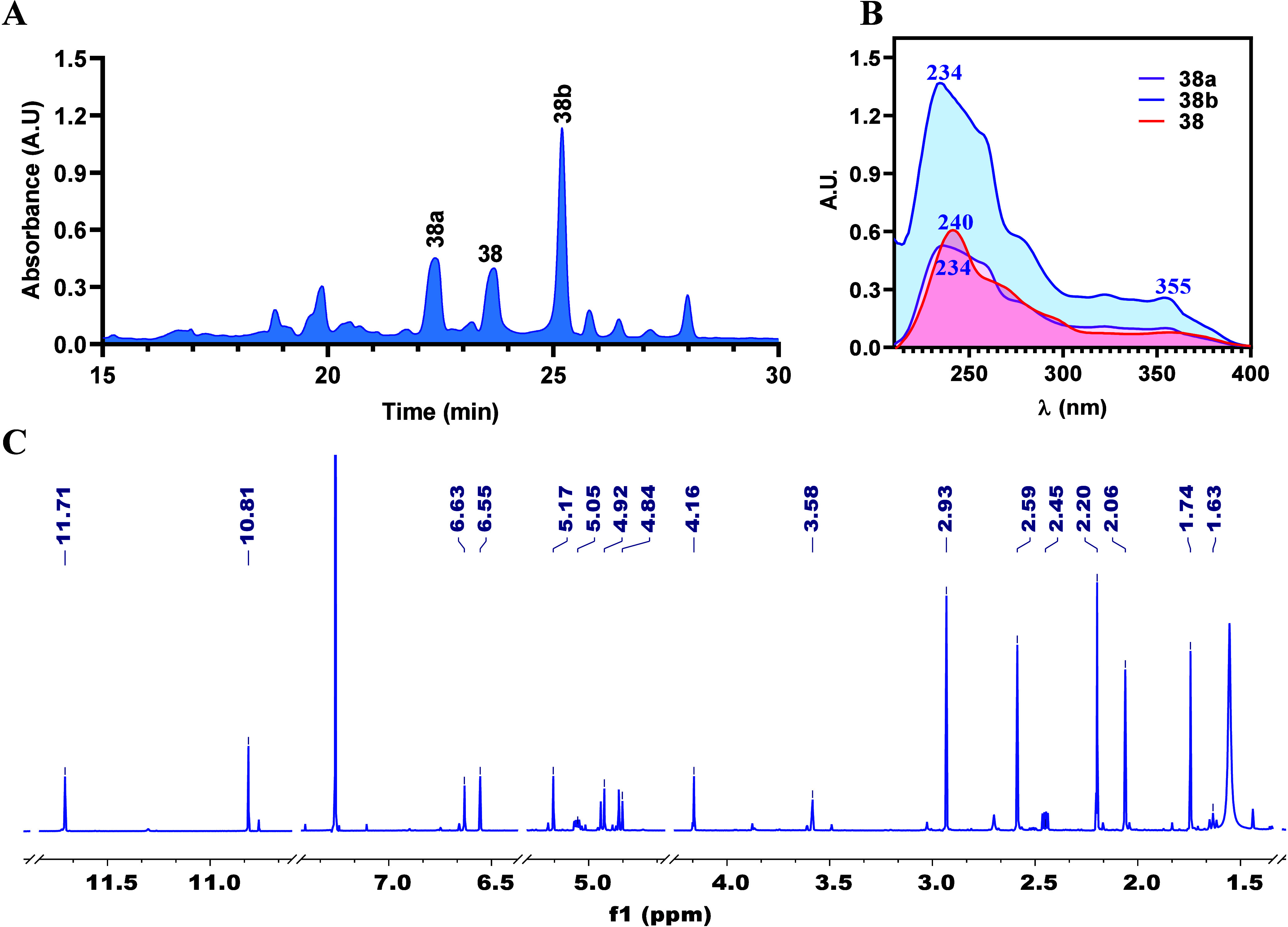
HPLC profile of the reaction mixture of **1** with acetone/morpholine/formic acid to generate **38**. B) UV profile of the most abundant components of the reaction mixture (**38a**, **38**, and **38b**). C) ^1^H NMR spectrum recorded for **38** in CDCl_3_ at 700 MHz.

TLC of the processed reaction mixture after extraction with H_2_O and CH_2_Cl_2_ revealed that **1** had been completely converted into a less polar compound. HPLC analysis (Figure 7A) of the reaction mixture showed three products with UV profiles similar to those reported for verruculosin A (**38**)^46^ (λ_max_ 240 and 355 nm, Figure 7B). Using semipreparative HPLC, the three major components were successfully isolated. ^1^H NMR analysis of the isolated compounds confirmed the transformation of compound **1** into compound **38** (Figure 7C). Additionally, two new analogues of verruculosin A (**38a** and **38b**, verruculosins C and D, respectively) were identified. Their structures were established by ^1^H NMR (Table 1) and NOESY experiments. Coupling constant analysis between H-1 and H_2_-11 indicated that the relative configuration at C-1 in verruculosin A derivatives remains the same as that in verruculosin A (**38**). Considering these facts, the observed NOESY interactions and the proposed reaction mechanism to generate **38**, the main difference between **38a** and **38b** is the relative configuration at C-12.

**Table 1:**
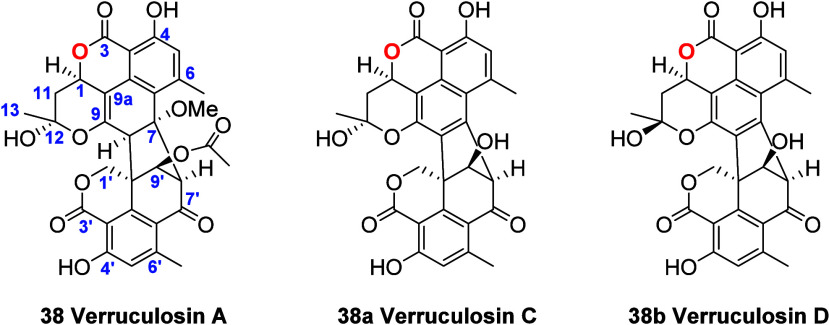
^1^H and ^13^C NMR Spectroscopic Data for **38a** and ^1^H NMR for **38b**, Recorded in CDCl_3_, at 175 and 700 MHz, respectively

	**38a**		**38b**	
no.	δ_C_, type	δ_H_ (*J* in Hz)	δ_C_, type	δ_H_ (*J* in Hz)
**1**	70.3, CH	5.66, dd (11.9, 5.8)	70.3, CH	5.82, d (11.9, 5.7)
**3**	170.4, C	-	170.2, C	
**3a**	99.0, C		98.9, C	
**3b**	131.5, C		131.8, C	
**4**	163.6, C		163.6, C	
**5**	120.8, CH	6.90, s	121.0, CH	6.93, brs
**6**	146.6, C		146.4, C	
**7**	120.1, C		119.9, C	
**8**	137.6, C		137.4, C	
**9**	131.1, C		130.9, C	
**9a**	147.4, C		147.5, C	
**10**	25.0, CH_3_	3.01, s	25.2, CH_3_	3.02, s
**11**	36.4, CH_2_	2.72, dd (12.3, 5.8)	36.6, CH_2_	2.71, dd (12.5, 5.7)
		2.29, t (12.3)		2.19, t (12.2)
**12**	100.1, C	-	100.2, C	
13	29.2, CH_3_	1.87, s	29.1, CH_3_	1.80, s
**1′**	70.3, CH_2_	5.13, d (12.7)	70.4, CH_2_	5.12, d (12.7)
		5.03, d (12.7)		5.01, d (12.7)
**3′**	168.8, C	-	168.0, C	
**3′a**	103.5, C	-	103.7, C	
**3′b**	147.0, C	-	146.8, C	
**4′**	164.4, C	-	164.8, C	
**5′**	121.1, CH	6.75, s	121.1, CH	6.76, brs
**6′**	154.0, C	-	154.0, C	
**6′a**	117.0, C	-	117.0, C	
**7′**	191.5, C		191.5, C	
**8′**	65.5, CH	4.94, s	65.1, CH	4.95, s
**9′**	86.4, CH	4.78, d (5.6)	86.5, CH	4.77, brs
**10′**	24.0, CH_3_	2.57, s	24.0, CH_3_	2.56, brs
**4-OH**	-	11.50, s	-	11.67, s
**4′-OH**	-	11.71, s	-	11.89, s

Interestingly, while writing this work, a series of nine new oligophenalenones (adpresisins A-G; **39**–**47**) from *Talaromyces adpressus* were reported by Zheng et al.^47^ Based on the outcomes from our research, it is plausible to consider that at least seven of them are artifacts (adpressins B–F and the corresponding C-1 epimers of adpressins B and C), generated by the C-9′ acetate hydrolysis and/or the C-7 MeOH elimination reactions and by a combination of **1** and primary amines via the proposed mechanisms discussed herein.

**Chart 2:**
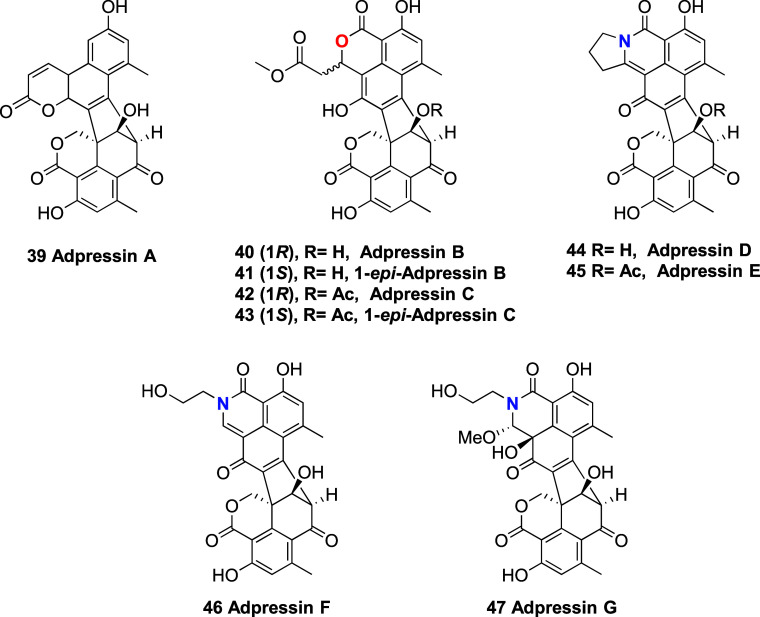


**Are other molecules within the duclauxin family authentic enzymatic products?** Given the findings presented above, it is timely to raise the question of whether other duclauxin analogs, such as verruculosin B (**48**),^46^ talarocketals A-B (**49**–**50**),^18^ talaroclauxins A-B (**51**–**52**),^17^ macrosporousones A–C (**53**–**55**),^30^ and bacillisporins A-B (**56**–**57**), D-E (**58**–**60**),^30^ are truly produced by specialized enzymes, or if the generation of such derivatives is simply a consequence of the highly reactive nature of the C-1 to C-9 α,β-unsaturated carbonyl segment of **1**, which undergoes transformations under certain extraction/isolation conditions, and even in the intracellular environment. In any case, this work underscores that the reactivity exhibited by duclauxin (**1**) can be harnessed to facilitate the semisynthesis or directed biosynthesis of a broader spectrum of pharmacologically valuable dimeric phenalenones.

**Chart 3:**
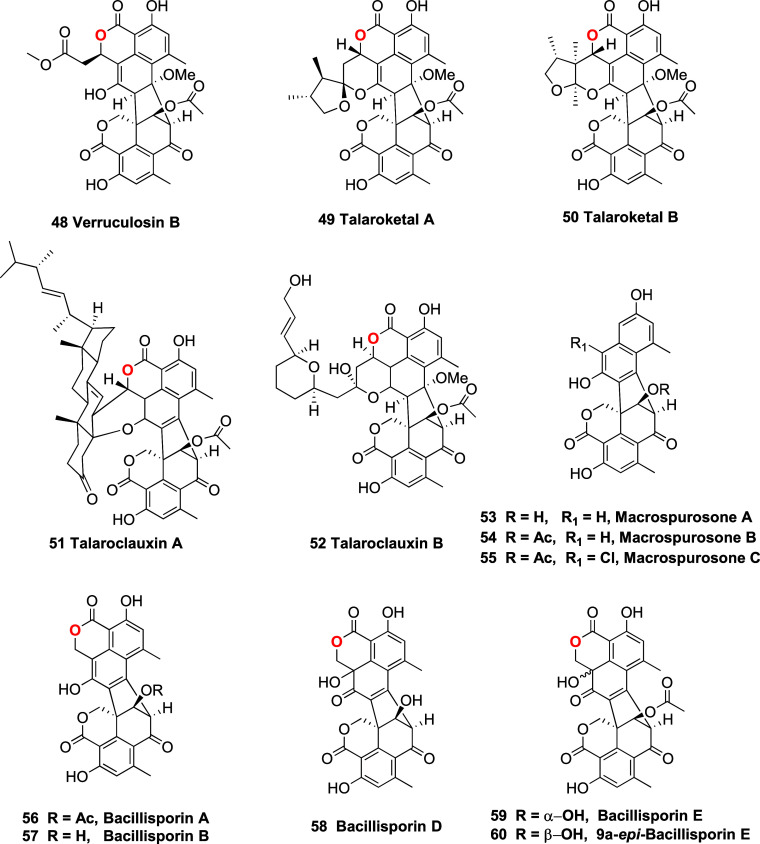


Altogether, the evidence supports that many members of the duclauxin family are in fact nonenzymatic products or even artifacts generated during the extraction and isolation process, due to the promiscuity of **1** that reacts with several alcohols, exocyclic or endocyclic-enols, or primary amines and might suffer transformations (oxidation reactions) and rearrangements under mild conditions.

## Experimental Section

### General Experimental Procedures

NMR experiments, both 1D and 2D, were carried out using CDCl_3_ or CD_3_OD as solvents. The NMR setup comprised either a Bruker Ascend III 700 MHz NMR spectrometer featuring a cryoprobe, operating at 700 MHz for ^1^H and 175 MHz for ^13^C, or a Bruker 500 Ascend equipped with an autosampler, operating at 500 MHz for ^1^H and 125 MHz for ^13^C. Chemical shifts are reported in parts per million relative to the solvent resonances as the internal standard (CDCl_3_ δ_H_/δ_C_ 7.26/77.16; CD_3_OD δ_H_/δ_C_ 3.31/49.00). High-resolution mass spectrometry data were obtained using a Jeol, AccuTOF JMS-T100LC mass spectrometer (HR-DART-MS), or an Agilent, 6530 Accurate-Mass Quadrupole Time of Flight (Q-TOF) LC/MS system or a Q-exactive Hybrid Quadrupole-Orbitrap LC/MS system. The structures of all phenalenones were determined by comparison of UV profiles and retention times to previously characterized references.

### Fermentation of *Talaromyces* sp. IQ-313

Fungal fermentation was performed as previously described by Aguilar-Ramírez et al. 2023.^16^ Briefly, a seed culture of the fungal strain *Talaromyce*s sp. IQ-313 was fermented on potato-dextrose-agar (PDA). Then, the mycelium was transferred into potato-dextrose broth (PDB, 15 mL × 10) and incubated for 7 days under orbital shaking at 100 rpm. Each seed culture was then transferred into an E-flask (250 mL) containing 12 g of autoclaved rice and incubated at room temperature for 21 days.

### Extraction

Each flask of solid fermentation was extracted with 60 mL of EtOAc and shaken for 24 h at 100 rpm. Next, the contents of all flasks were gathered, the biomass was removed through filtration, and 300 mL of H_2_O was added to a final volume of 900 mL. This mixture was stirred for 15 min and partitioned. The organic phase was evaporated to dryness, and the residue was resuspended in 200 mL of CH_3_CN and partitioned with 200 mL of hexanes (×3). Finally, the organic fraction was evaporated to give a final extract yield of 3.7 g.

### Chromatographic Analysis

All experiments were conducted using dry glassware, and monitoring was performed via HPLC-UV. Chromatographic analyses were executed in reversed-phase mode (C_18_, Gemini-NX, with a particle size of 5 μm, dimensions 4.6 mm × 250 mm; Phenomenex), employing a gradient ranging from 20% to 100% CH_3_CN:H_2_O/0.1% formic acid over 30 min. Samples were prepared at 1 mg/mL. Data acquisition and analysis were performed using Empower 3 software (Waters).

### Reactivity Studies

In each tested condition, 1 mg of extract was utilized and allowed to react for 24 h. Subsequently, a sample at a concentration of 1 mg/mL was subjected to analysis via reversed-phase HPLC, with the absorbance recorded at 254 nm. For kinetics assessment, the formation of products was monitored hourly.

#### Bacillisporin J (**8**/**9**)

1 mg of **1** was dissolved in 1 mL of DMSO and 30 μL of TEA, and stirred for 24 h at room temperature.

#### Bacillisporin I (**12**/**13**)

1 mg of **8**/**9** was dissolved in 1 mL of DMSO and 300 μL of HCl (1 N), and stirred for 24 h, at room temperature.

#### Bacillisporin F (and Epi-bacillisporin F) (**5**/**6**)

1 mg portion of **8**/**9** was dissolved in MeOH, and stirred for 36 h, at room temperature.

#### Xenoclauxin (**4**)

1 mg of **8**/**9** was adsorbed on 10 mg of silica gel for 5 days. After that, the mixture was dissolved in CH_2_Cl_2_ and stirred for 3 days. Finally, solvent was removed by decantation, and products were desorbed by partitioning with CH_3_CN.

#### Bacillsporin G (**3**)

10 mg of **1** was adsorbed on arbitrary mg of silica gel for 20 days. After that, the products were desorbed by partitioning with CH_3_CN.

#### Talaromycesone B (**2**)

5 mg of **1** was reacted with one equivalent of different amines, in 1 mL of DMSO, and stirred for 24 h, at room temperature. Alternatively, 1 mg of **8**/**9** was dissolved in 1 mL of DMSO with 300 μL of diethylentriamine and allowed to react for 5 days. Another method involved adding one drop of sodium hypochlorite (1:100) to 1 mg of **8**/**9** in 200 μL of DMSO.

### Isolation

#### Bacillisporin J (**8**/**9**)

989 mg of **1** was dissolved in 500 μL of DMSO and 300 μL of TEA, and stirred for 48 h, at room temperature. Following this, isolation was carried out using flash reversed-phase chromatography, utilizing a gradient of 50 to 80% CH_3_CN:H_2_O/0.1% formic acid, over 25 min.

#### Bacillisporin I (**12**/**13**)

Thirty mg of **8**/**9** was dissolved in 1 mL of DMSO and 300 μL of HCl (1 N), and stirred for 14 days, at room temperature. Following this, isolation was carried out using reversed-phase HPLC, employing a gradient of 50 to 90% CH_3_CN:H_2_O/0.1% formic acid, over 30 min.

#### Xenoclauxin (**4**)

50 mg of **8**/**9** was dissolved in 50 mL of CH_2_Cl_2_ and 500 mg of silica gel, and stirred for 4 days, at room temperature. The supernatant was kept and evaporated to dryness, and after that, isolation was carried out using HPLC in reversed phase, employing a gradient of 60 to 90% CH_3_CN:H_2_O/0.1% formic acid, over 30 min.

#### Verruculosin A (**38**)

To 10 mg of **1** dissolved in acetone (300 μL) was added 200 μL of a 1:1 mixture of acetone-morpholine, and the mixture was stirred for 24 at room temperature. Subsequently, to the reaction mixture 50 μL of formic acid was added and the mixture was allowed to react for 1 h. Afterward, the reaction was stopped by adding 5 mL of H_2_O and extracted (twice) with 10 mL of CH_2_Cl_2_. Consecutively, the organic layer was filtered through anhydrous sodium sulfate and dried in vacuo. Following this, isolation was carried out using reversed-phase HPLC, employing a gradient of 35 to 85% CH_3_CN:H_2_O/0.1% formic acid, over 30 min, in a Luna PFP column (10 × 250 mm, 5 μm particle size, Phenomenex).

## Data Availability

Raw NMR data for molecules **38a** and **38b** have been deposited to the NP-MRD database (NP0333733 and NP0333734, respectively).

## Supporting Information Available

Chromatographic profiles for compounds **1**–**6**, **8**, and **9**. Proposed mechanisms of reactions to access talauxins, talauxamides, talaramides, talaroquinolines, and talaropyridines from duclauxin (**1**). Copies of ^1^H and ^13^C and 2D NMR spectra (and tables of NMR data) and HR-MS data for each compound. Molecular Formula Strings (PDF)

## Notes

This work was taken in part from the PhD Thesis of Enrique Aguilar-Ramírez.
