# Supplementary material for: Exploring the Nonenzymatic Origin of Duclauxin-like Natural Products
Source: J Nat Prod. 2024 Sep 10;87(9):2230–42. doi: 10.1021/acs.jnatprod.4c00558 (PMC11443492; doi:10.1021/acs.jnatprod.4c00558)
Supplement: Supplementary file 1 — np4c00558_si_001.pdf [file np4c00558_si_001.pdf]

Exploring the Nonenzymatic Origin of Duclauxin-like Natural Products

Enrique Aguilar-Ramírez,[†] José Rivera-Chávez[†,*], Brandon D. Alvarado-Zacarías,[†] and José E. Barquera-Lozada[§]

[†]Department of Natural Products, Institute of Chemistry, Universidad Nacional Autónoma de México, Mexico City, 04510, México.

[§] Department of Physicalchemistry, Institute of Chemistry, Universidad Nacional Autónoma de México, Mexico City, 04510, México.

[*]**Correspondence**: Dr. José Rivera-Chávez.

Phone number: +52 55 5622-4450.

E-mail: jrivera@iquimica.unam.mx.

ORCID: 0000-0002-9225-6200

Table of contents

Figures

**Figure S1**. Structure of analogues of duclauxin family.	**S4**
**Figure S2**. Chromatographic and UV profiles for standard references of compounds **1**-**6**, **8** and **9**.	**S9**
**Figure S3**. MS spectrum of **1** obtained by DART *m/z*: 547.1254 $[M+H]^+$ (calcd. for $C_{29}H_{23}O_{11}$, 547.1240, Δ +2.6 ppm).	**S13**
**Figure S4**. ^1H spectrum of **1** recorded in $CDCl_3$ at 500 MHz.	**S13**
**Figure S5**. ^{13}C NMR spectrum of **1** recorded in $CDCl_3$ at 125 MHz.	**S13**
**Figure S6**. HSQC NMR spectrum of **1** (500 MHz, $CDCl_3$).	**S14**
**Figure S7**. HMBC NMR spectrum of **1** (500 MHz, $CDCl_3$).	**S14**
**Figure S8**. MS spectrum of **2** obtained by AJS-Q-TOF *m/z*: 503.0997 $[M+H]^+$ (calcd. for $C_{27}H_{19}O_{10}$, 503.0978, Δ +3.8 ppm).	**S15**
**Figure S9**. ^1H spectrum of **2** recorded in CD_3OD at 500 MHz.	**S15**
**Figure S10**. ^{13}C NMR spectrum of **2** recorded in CD_3OD at 125	**S15**
**Figure S11**. HSQC NMR spectrum of **2** (500 MHz, $CDCl_3$).	**S16**
**Figure S12**. HMBC NMR spectrum of **2** (500 MHz, $CDCl_3$).	**S16**
**Figure S13**. MS spectrum of **3** obtained by AJS-Q-TOF *m/z*: 489.1180 $[M+H]^+$ (calcd. for $C_{27}H_{21}O_9$, 489.1186, Δ -1.2 ppm).	**S17**
**Figure S14**. ^1H spectrum of **3** recorded in $CDCl_3$ at 500 MHz.	**S17**
**Figure S15**. ^1H spectrum of **3** recorded in $CDCl_3$ at 125 MHz.	**S17**
**Figure S16**. HSQC NMR spectrum of **3** (500 MHz, $CDCl_3$).	**S18**
**Figure S17**. HMBC NMR spectrum of **3** (500 MHz, $CDCl_3$).	**S18**
**Figure S18**. MS spectrum of **4** obtained by AJS-Q-TOF *m/z*: 531.0936 $[M+H]^+$ (calcd. for $C_{28}H_{19}O_{11}$, 531.0927, Δ +1.7 ppm).	**S19**
**Figure S19**. ^1H spectrum of **4** recorded in $CDCl_3$ at 400 MHz.	**S19**
**Figure S20**. ^{13}C NMR spectrum of **4** recorded in $CDCl_3$ at 100 MHz.	**S19**
**Figure S21**. HSQC NMR spectrum of **4** (400 MHz, $CDCl_3$).	**S20**
**Figure S22**. HMBC NMR spectrum of **4** (400 MHz, $CDCl_3$).	**S20**
**Figure S23**. MS spectrum of **5** obtained by AJS-Q-TOF *m/z*: 547.1248 $[M+H]^+$ (calcd. for $C_{29}H_{23}O_{11}$, 547.1240, Δ +1.5 ppm).	**S21**
**Figure S24**. ^1H spectrum of **5** recorded in $CDCl_3$ at 500 MHz.	**S21**
**Figure S25**. ^{13}C NMR spectrum of **5** recorded in $CDCl_3$ at 125 MHz.	**S21**
**Figure S26**. HSQC NMR spectrum of **5** (500 MHz, $CDCl_3$).	**S22**
**Figure S27**. HMBC NMR spectrum of **5** (500 MHz, $CDCl_3$).	**S22**
**Figure S28**. MS spectrum of **6** obtained by AJS-Q-TOF *m/z*: 547.1260 $[M+H]^+$ (calcd. for $C_{29}H_{23}O_{11}$, 547.1240, Δ +3.7 ppm).	**S23**
**Figure S29**. ^1H spectrum of **6** recorded in $CDCl_3$ at 500 MHz.	**S23**
**Figure S30**. ^{13}C NMR spectrum of **6** recorded in $CDCl_3$ at 125 MHz.	**S23**
**Figure S31**. HSQC NMR spectrum of **6** (500 MHz, $CDCl_3$).	**S24**
**Figure S32**. HMBC NMR spectrum of **6** (500 MHz, $CDCl_3$).	**S24**
**Figure S33**. MS spectrum of **8**/**9** obtained by Q-Exactive *m/z*: 531.0924 $[M-H]^-$ (calcd. for $C_{28}H_{19}O_{11}$, 531.0927, Δ -0.6 ppm).	**S25**
**Figure S34**. ^1H-NMR spectrum of **8**/**9** recorded in $CDCl_3$ at 700 MHz.	**S26**
**Figure S35**. ^{13}C-NMR spectrum of **8**/**9** recorded in $CDCl_3$ at 175 MHz.	**S26**
**Figure S36**. HSQC NMR spectrum of **8**/**9** recorded in $CDCl_3$ at 700 MHz.	**S27**
**Figure S37**. HMBC NMR spectrum of **8**/**9** recorded in $CDCl_3$ at 700 MHz.	**S27**
**Figure S38**. MS spectrum of **12**/**13** obtained by DART *m/z*: 473.0835 $[M+H^+-H_2O]^+$ (calcd. for $C_{26}H_{17}O_9$, 473.0873, Δ -8.0 ppm).	**S28**

Figure S39. ^{1}H-NMR spectrum of **12**/**13** recorded in $CDCl_3$ at 700 MHz. **S29**
Figure S40. ^{13}C-NMR spectrum of **12**/**13** recorded in $CDCl_3$ at 175 MHz. **S29**
Figure S41. HSQC NMR spectrum of **12**/**13** recorded in $CDCl_3$ at 700 MHz. **S30**
Figure S42. HMBC NMR spectrum of **12**/**13** recorded in $CDCl_3$ at 700 MHz. **S30**
Figure S43. ^{1}H-NMR spectrum of **38** recorded in $CDCl_3$ at 700 MHz. **S32**
Figure S44. ^{1}H-NMR spectrum of **38a** recorded in $CDCl_3$ at 700 MHz. **S32**
Figure S45. ^{13}C-NMR spectrum of **38a** recorded in $CDCl_3$ at 175 MHz. **S33**
Figure S46. HSQC NMR spectrum of **38a** recorded in $CDCl_3$ at 700 MHz. **S33**
Figure S47. HMBC NMR spectrum of **38a** recorded in $CDCl_3$ at 700 MHz. **S34**
Figure S48. NOESY NMR spectrum of **38a** recorded in $CDCl_3$ at 700 MHz. **S34**
Figure S49. ^{1}H-NMR spectrum of **38b** recorded in $CDCl_3$ at 700 MHz. **S35**
Figure S50. ^{13}C-NMR spectrum of **38b** recorded in $CDCl_3$ at 700 MHz. **S35**
Figure S51. HSQC NMR spectrum of **38b** recorded in $CDCl_3$ at 700 MHz. **S36**
Figure S52. HMBC NMR spectrum of **38b** recorded in $CDCl_3$ at 700 MHz. **S36**
Figure S53. NOESY NMR spectrum of **38b** recorded in $CDCl_3$ at 700 MHz. **S37**
Figure S54. Chromatographic profile of talaramide M recorded at 254 nm, showing the co-existence of both, *P* and *M* atropisomers. **S37**

Tables

Table S1. Spectroscopic data of ^{1}H-NMR for **1-6** products, recorded in $CDCl_3$ (compounds **1**, **3-6**) and MeOD (compound **2**). Note: All spectroscopic and spectrometric data presented herein were obtained from article "Harnessing the Reactivity of Duclauxin toward Obtaining *h*PTP1B$_{1-400}$ Inhibitors" **S11**
Tabla S2. Spectroscopic data of ^{13}C-NMR for **1-6** products, recorded in $CDCl_3$ (compounds **1**, **3-6**) and MeOD (compound **2**). Note: All spectroscopic and spectrometric data presented herein were obtained from article "Harnessing the Reactivity of Duclauxin toward Obtaining *h*PTP1B$_{1-400}$ Inhibitors" **S12**
Table S3. Spectroscopic data of ^{13}C-NMR and ^{1}H-NMR for **8**/**9** and **12**/**13**, recorded in $CDCl_3$, at 175 MHz and 700 MHz, respectively. **S31**

Schemes

Scheme S1. General mechanism for the formation of enamines from ketones. **S38**

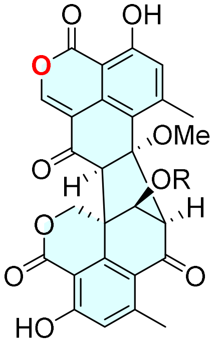

R = Ac, Duclauxin
R = H, Desacetylduclauxin

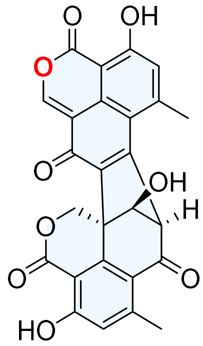

Neoclauxin

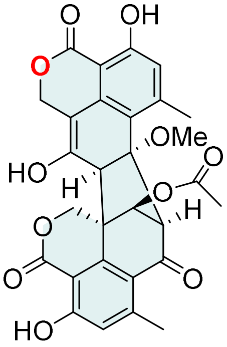

Talaromycesone A

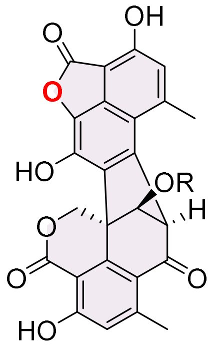

R = Ac, Talaromycesone B
R = H, Talaromycesone C

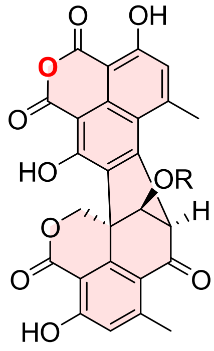

R = Ac, Xenoclauxin
R = H, Macrospurosone D

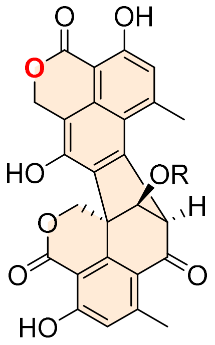

R = Ac, Bacillisporin A
R = H, Bacillisporin B

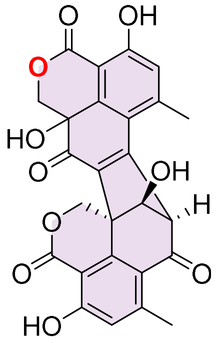

Bacillisporin D

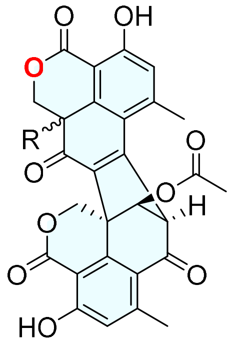

R = α–OH, Bacillisporin E
R = β–OH, 9a-*epi*-Bacillisporin E

Figure S1. Structure of analogues of duclauxin family.

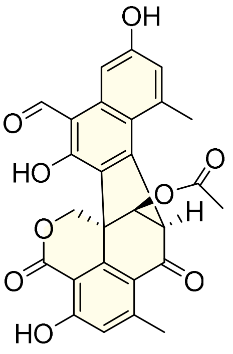

Bacillisporin G

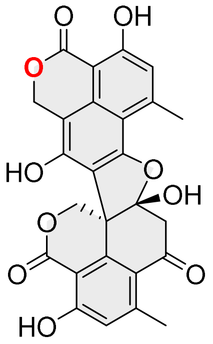

Bacillosporin C

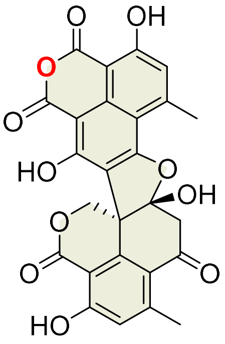

Bacillosporin D

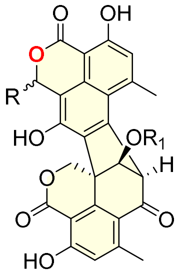

R = β–OMe, R_1= Ac, Bacillisporin F
R = α–OMe, R_1= Ac, 1-*epi*-Bacillisporin F
R = β–OH, R_1= Ac, Bacillisporin I
R = α–OH, R_1= Ac,1-*epi*-Bacillisporin I
R = β–OH, R_1= H, Bacillisporin J
R = α–OH, R_1= H, 1-*epi*-Bacillisporin J
R = α–2,3-dihydroxybutanil, R_1= H, Bacillisporin K
R = β–2,3-dihydroxybutanil, R_1= H, 1-*epi*-Bacillisporin L

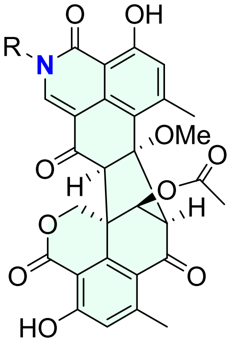

R = H, Bacillisporin H
R = L-Glutamic acid, Talauxin E
R = L-Glutamine, Talauxin Q
R = L-Valine, Talauxin V
R = L-Leucine, Talauxin L
R = L-Isoleucine, Talauxin I

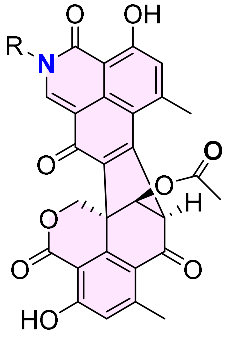

Duclauxamide A1, R = 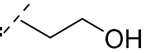

Duclauxamide B, R = 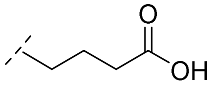

Duclauxamide C, R = 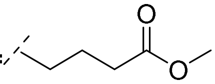

Figure S1 (continued). Structure of analogues of duclauxin family.

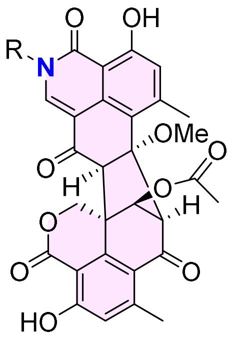

Talaroclauxin C, R = 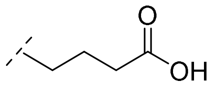

Talaroclauxin D, R = 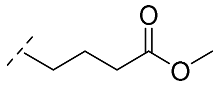

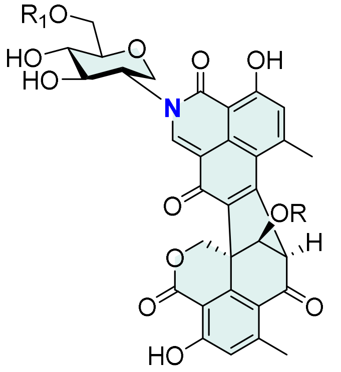

R = H, R1 = H, Glyclauxin A
R = Ac, R1 = H, Glyclauxin B
R = Ac, R1 = Ac, Glyclauxin E

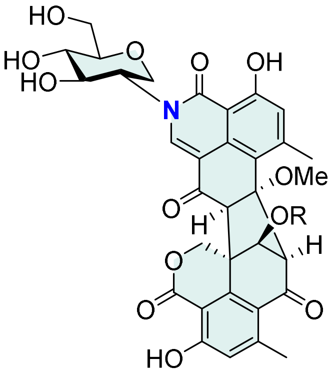

R = Ac, Glyclauxin C
R = H,   Glyclauxin D

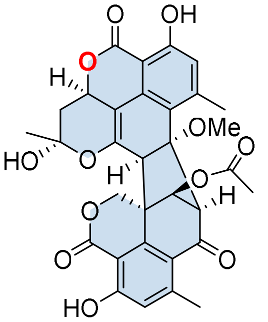

Verruculosin A

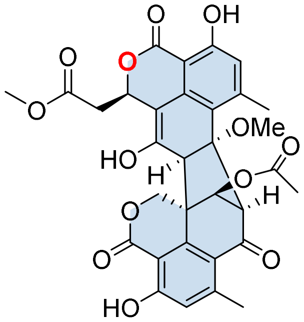

Verruculosin B

Figure S1 (Continued). Structure of analogues of duclauxin family.

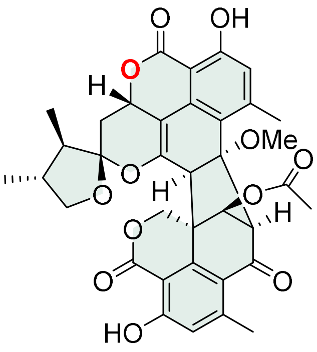

Talaroketal A

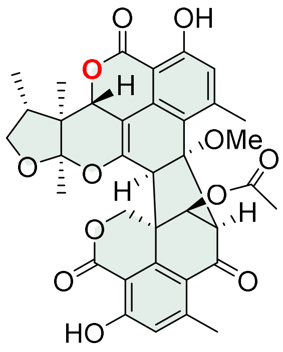

Talaroketal B

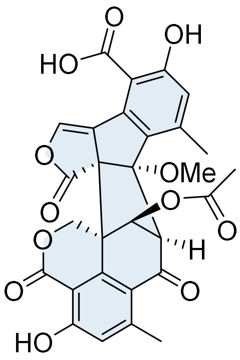

Talaverrucin A

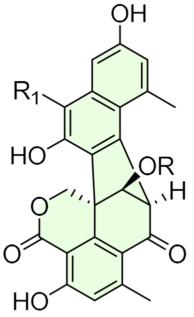

R = H, R_1 = H, Macrospurosone A
R = Ac, R_1 = H, Macrospurosone B
R = Ac, R_1 = Cl, Macrospurosone C

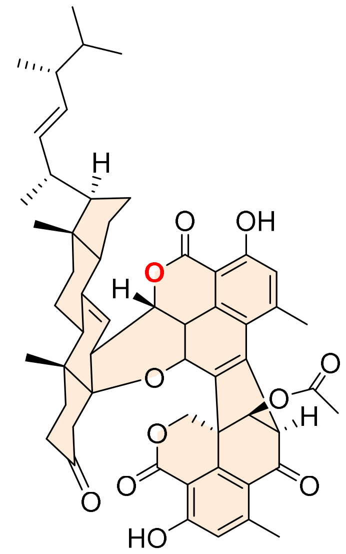

Talaroclauxin A

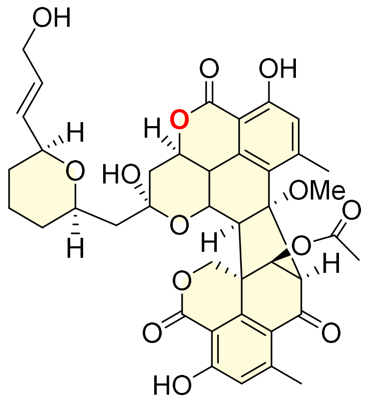

Talaroclauxin B

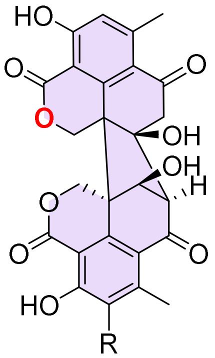

R = Cl, Gilmaniellin
R = H, Dechlorogilmaniellin

Figure S1 (Continued). Structure of analogues of duclauxin family.

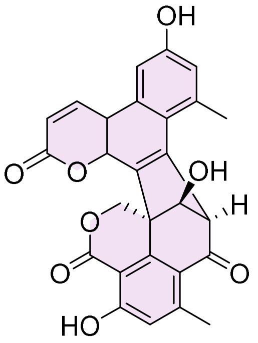

39 Adpressin A

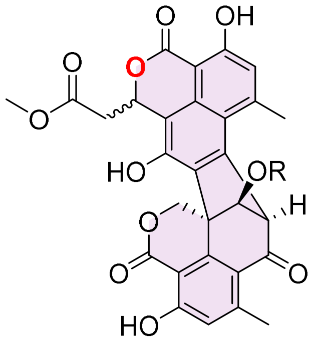

40 (1*R*), R= H, Adpressin B
41 (1*S*), R= H, 1-*epi*-Adpressin B
42 (1*R*), R= Ac, Adpressin C
43 (1*S*), R= Ac, 1-*epi*-Adpressin C

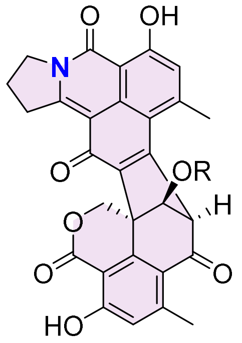

44 R= H, Adpressin D
45 R= Ac, Adpressin E

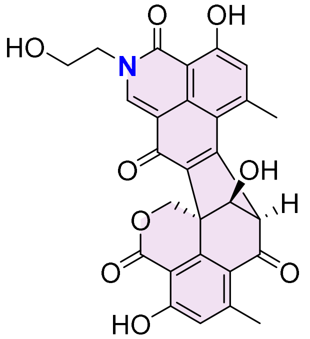

46 Adpressin F

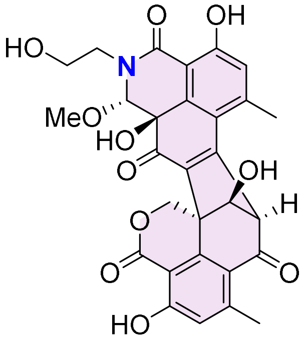

47 Adpressin G

Figure S1 (Continued). Structure of analogues of duclauxin family.

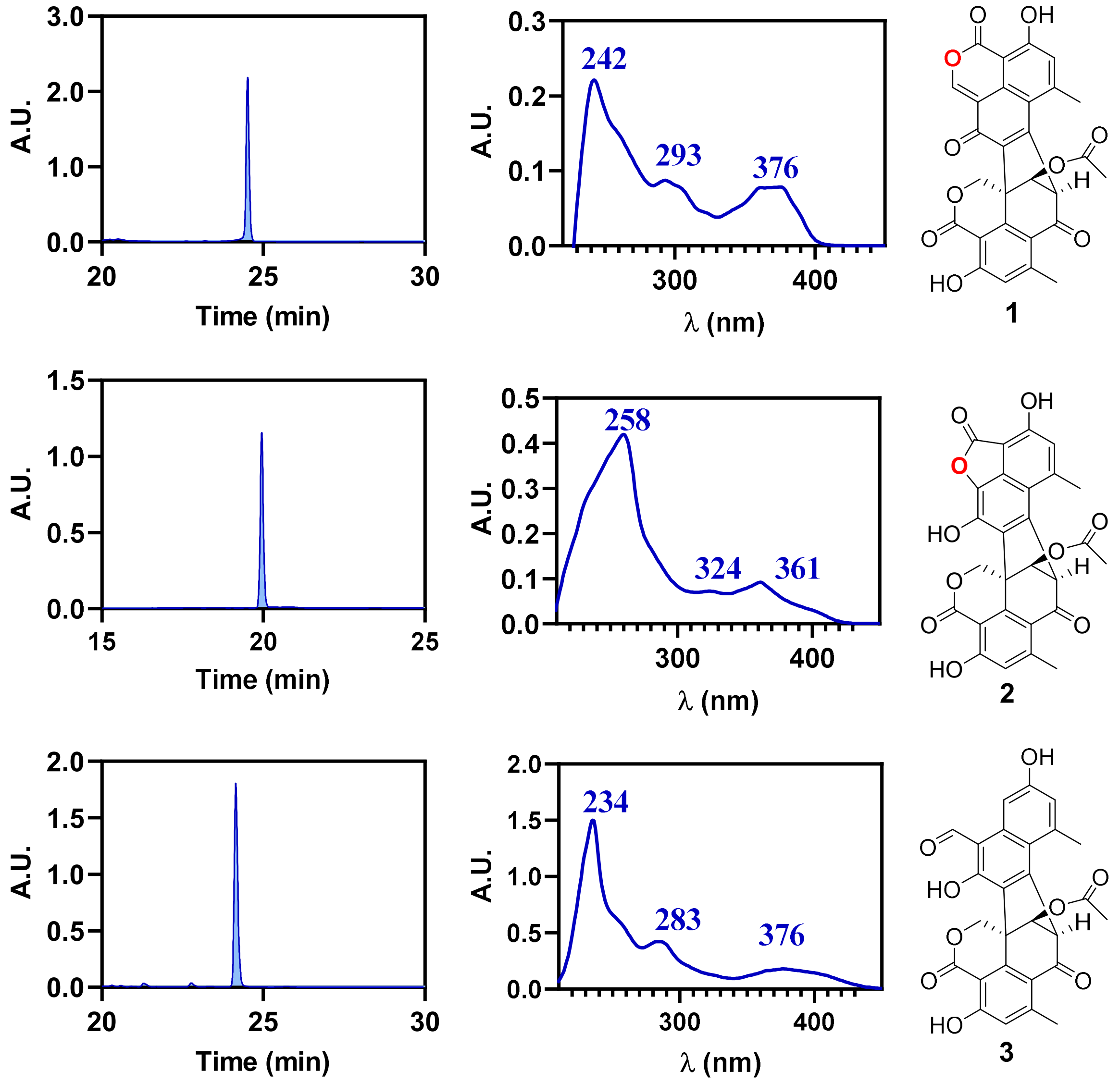

Figure S2. Chromatographic and UV profiles for standard references of compounds **1-6**, **8** and **9**.

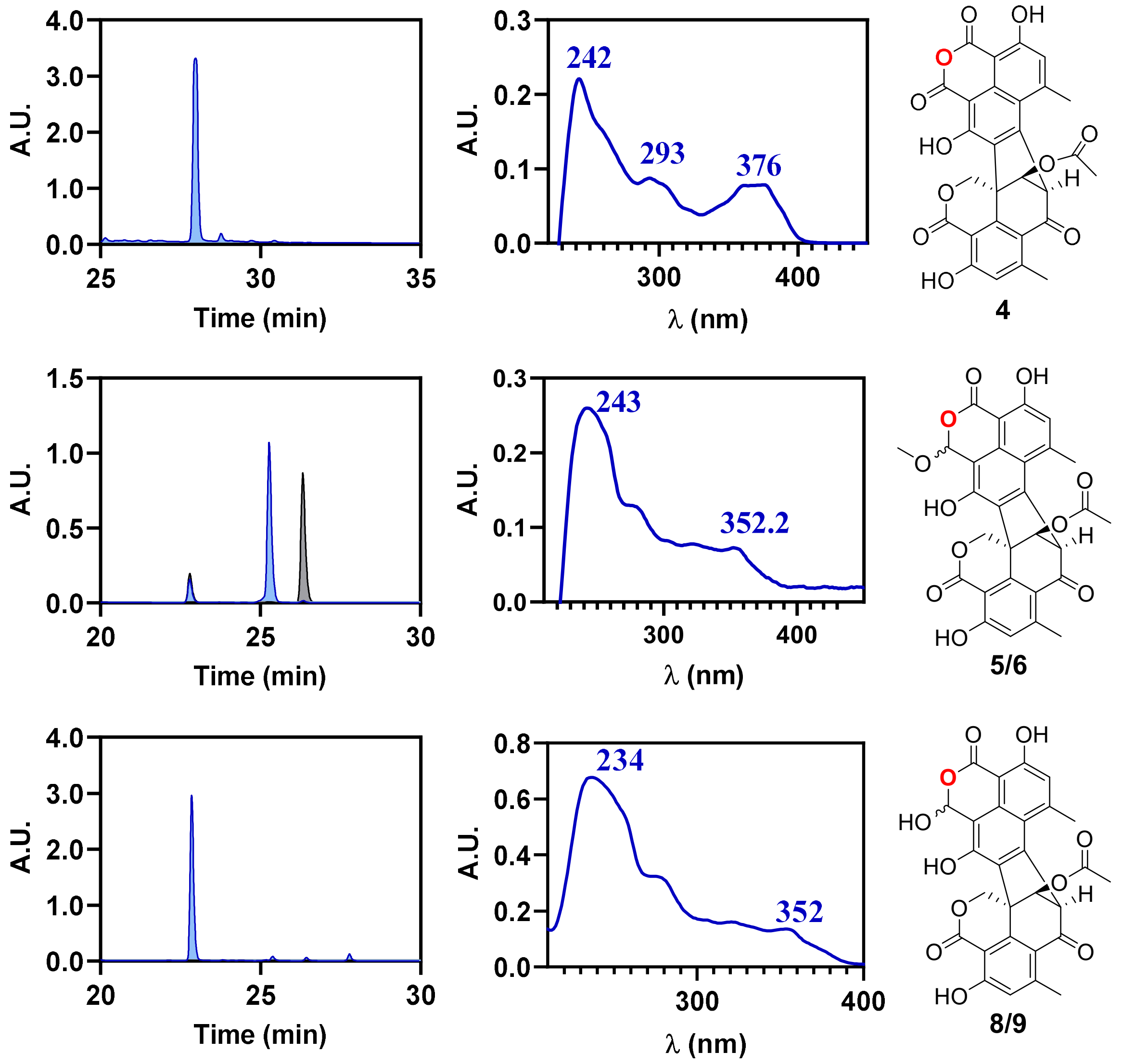

Figure S2 (Continued). Chromatographic and UV profiles for standard references of compounds **1**-**6**, **8** and **9**.

Table S1. Spectroscopic data of ^{1}H-NMR for **1**, **3-6** products, recorded in $CDCl_3$ (compounds **1**, **3-6**) and MeOD (compound **2**). Note: All spectroscopic and spectrometric data presented herein were obtained from article "Harnessing the Reactivity of Duclauxin toward Obtaining *h*PTP1B$_{1-400}$ Inhibitors"

**Position**	**δ_H, mult. (J en Hz)**					
	**1**^{b}	**2**^{b}	**3**^{b}	**4**^{c}	**5**^{b}	**6**^{b}
1	7.71, s	-	10.57, s	-	6.57, s	6.50, s
2	-	-	-	-	-	-
3a	-	-	7.55, s	-	-	-
5	6.91, s	6.86, d (1.0)	6.88, s	7.05, s	6.94, s	6.94, d (0.7)
8	3.98, s	-	-	-	-	-
10	2.74, s	2.91, d (0.8)	3.00, s	3.10, s	3.01, s	3.00, s
OCH_3	2.97, s	-	-	-	3.74, s	3.80, s
1′α	5.08, d (12.2)	5.16, d (12.4)	5.14, d (12.3)	5.17, d (12.4)	5.05, d (12.1)	5.11, d (12.4)
1′β	4.78, d (12.2)	5.04, d (12.4)	4.90, d (12.2)	4.94, d (12.5)	4.96, d (12.4)	4.95, d (12.5)
5′	6.64, d (0.7)	6.76, d (0.9)	6.78, s	6.82, s	6.82, s	6.81, d (0.7)
8′	4.14, s	4.80, d (1.1)	5.17, s	5.15, brs	5.06, d (1.1)	5.08, (1.2)
9′	5.20, s	5.85, d (1.1)	5.81, s	5.86, brs	5.80, d (0.8)	5.80, (1.1)
10′	2.11, s	2.54, d (0.7)	2.57, s	2.57, s	2.59, s	2.59, s
4-OH	10.66, s	-	-	-	11.87, s	11.86, s
9-OH	-	-	13.40, s	-	7.45, s	11.60, s
4′-OH	11.71, s	-	11.98, s	-	11.58, s	11.60, s
9′-<u>CH3</u>COO	2.22, s	2.03, s	2.05, s	2.07, s	2.04, s	2.06, s

^{a}Recorded at 700 MHz, ^{b}Recorded at 500 MHz, ^{c}Recorded at 400 MHz.

Tabla S2. Spectroscopic data of ^{13}C-NMR for **1**, **3-6** products, recorded in $CDCl_3$ (compounds **1**, **3-6**) and MeOD (compound **2**). Note: All spectroscopic and spectrometric data presented herein were obtained from article "Harnessing the Reactivity of Duclauxin toward Obtaining *h*PTP1B$_{1–400}$ Inhibitors"

**Position**	δ_C					
	**1**[b]	**2**[b]	**3**[b]	**4**[c]	**5**[b]	**6**[b]
1	148.8	-	193.6	164.9	100.2	100.4
3	164.0	167.2	-	164.6	168.1	168.0
3a	101.5	100.4	101.0	97.5	97.1	97.2
3b	133.0	133.5	138.3	134.8	131.5	131.6
4	161.9	160.7	156.1	166.4	164.2	164.1
5	120.9	121.4	119.9	120.9	120.9	120.9
6	152.1	148.1	139.4	149.6	146.7	146.6
6a	118.4	117.5	120.9	118.5	119.6	119.6
7	88.9	132.7	147.2	131.9	139.6	139.5
8	64.1	136.6	131.1	145.5	130.8	130.7
9	193.8	137.4	160.8	161.4	150.2	150.1
9a	113.4	133.7	111.9	100.1	109.0	109.1
10	22.3	22.6	25.4	25.7	25.5	25.5
OCH_3	51.9	-	-	-	-	-
1′	71.5	70.5	68.9	68.6	69.6	69.3
3′	167.4	169.3	167.8	167.5	167.6	167.6
3′a	104.9	104.8	103.7	103.7	103.3	103.2
3′b	143.0	148.2	146.0	148.7	146.2	146.5
4′	164.9	165.5	164.9	165.2	165.0	164.9
5′	121.5	121.5	121.3	121.8	121.7	121.6
6′	152.1	154.6	154.0	154.2	154.8	154.7
6′a	121.0	118.0	116.9	116.5	117.3	117.1
7′	190.9	192.5	189.9	189.0	190.2	190.3
8′	67.5	62.2	63.1	62.9	62.0	62.0
9′	79.0	88.1	85.8	85.7	85.7	85.7
9′a	51.2	50.2	48.2	48.4	49.2	49.3
10′	22.8	23.9	24.1	24.1	24.1	24.1
$\underline{CH_3}COO$-9′	21.1	20.6	21.0	20.9	21.0	21.0
$CH_3\underline{C}OO$-9′	169.6	171.9	170.3	170.1	170.3	170.3

[a]Recorded at 175 MHz, [b]Recorded at 125 MHz, [c]Recorded at 100 MHz.

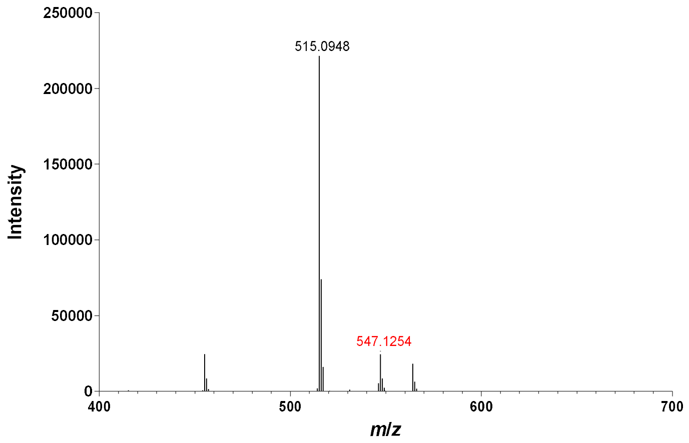

Figure S3. MS spectrum of **1** obtained by DART *m/z*: 547.1254 $[M+H]^+$ (calcd. for $C_{29}H_{23}O_{11}$, 547.1240, Δ +2.6 ppm).

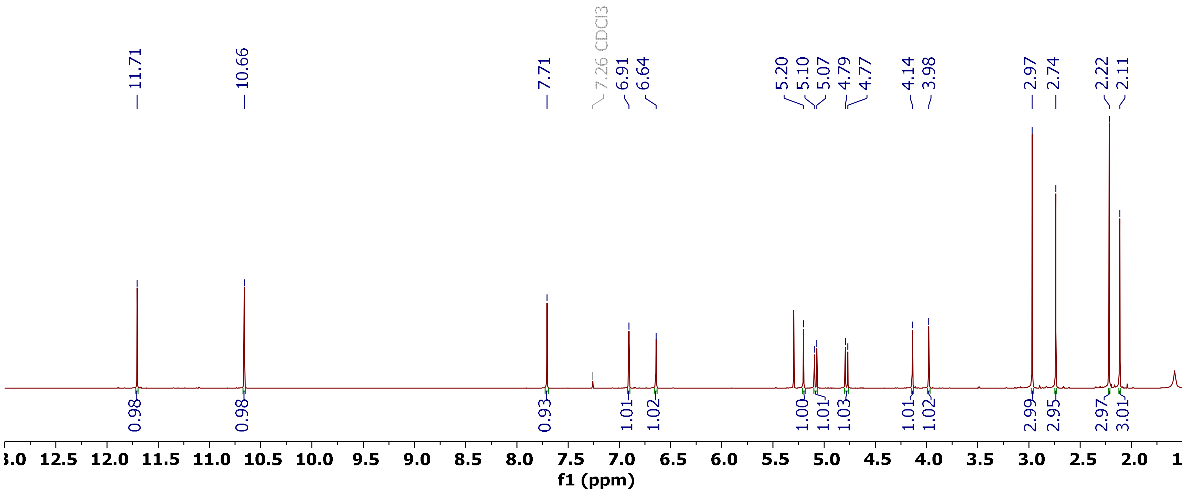

Figure S4. ^{1}H spectrum of **1** recorded in $CDCl_3$ at 500 MHz.

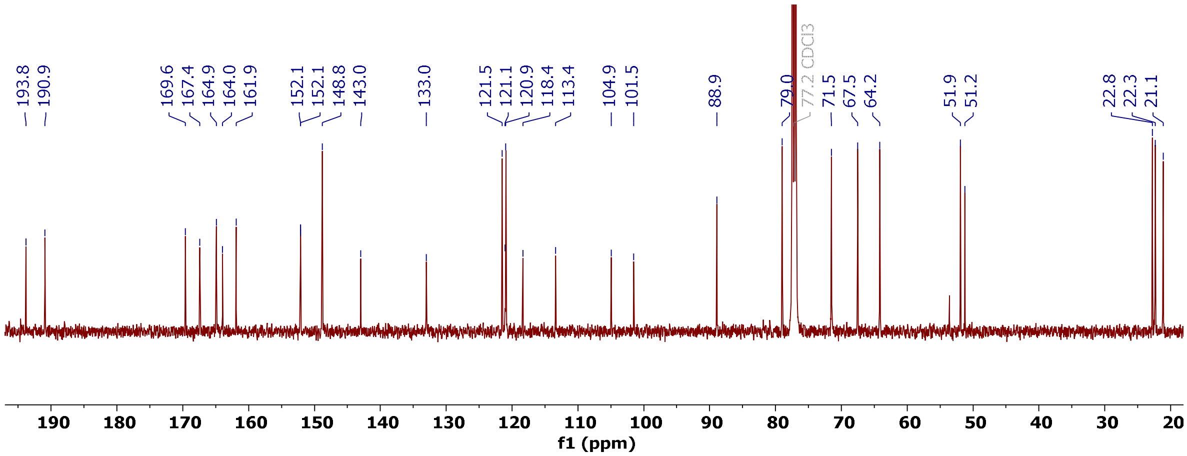

Figure S5. ^{13}C NMR spectrum of **1** recorded in $CDCl_3$ at 125 MHz.

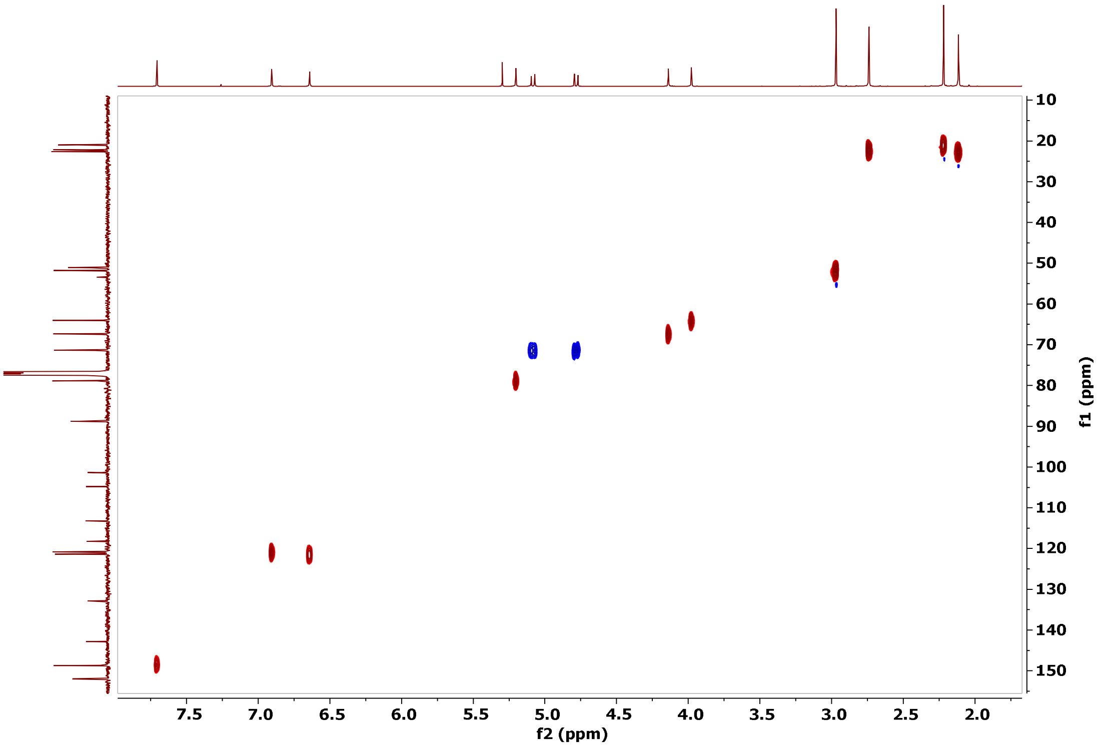

Figure S6. HSQC NMR spectrum of **1** (500 MHz, $CDCl_3$).

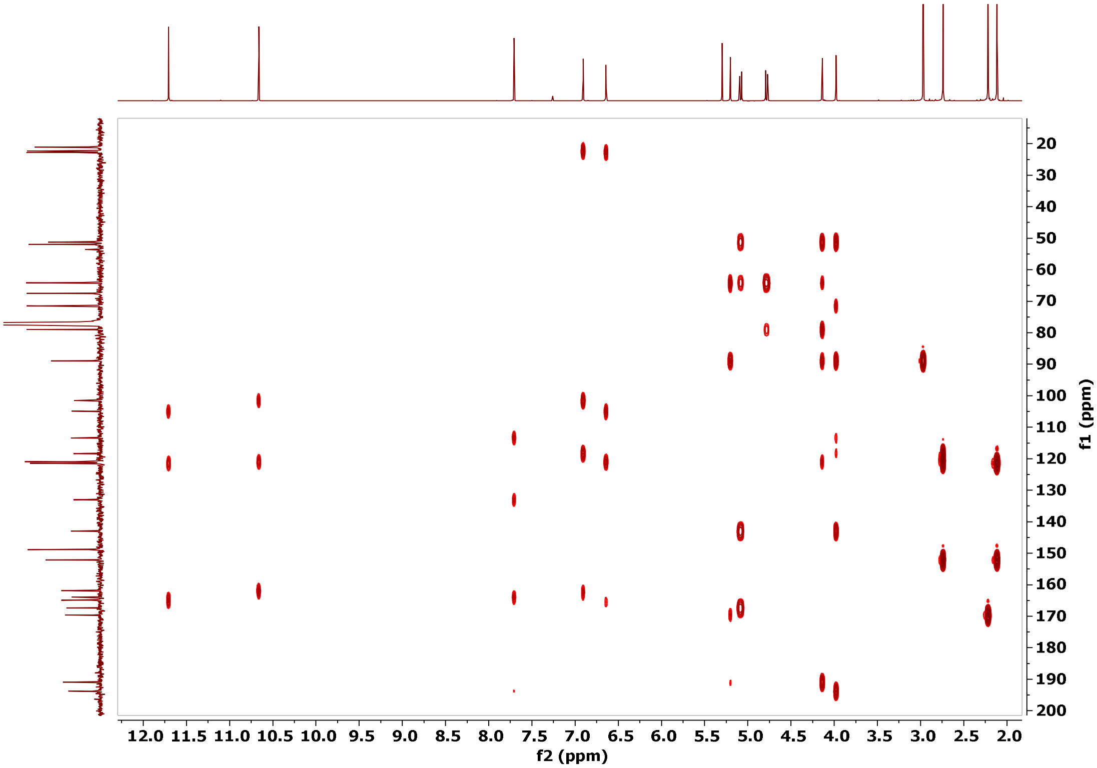

Figure S7. HMBC NMR spectrum of **1** (500 MHz, $CDCl_3$).

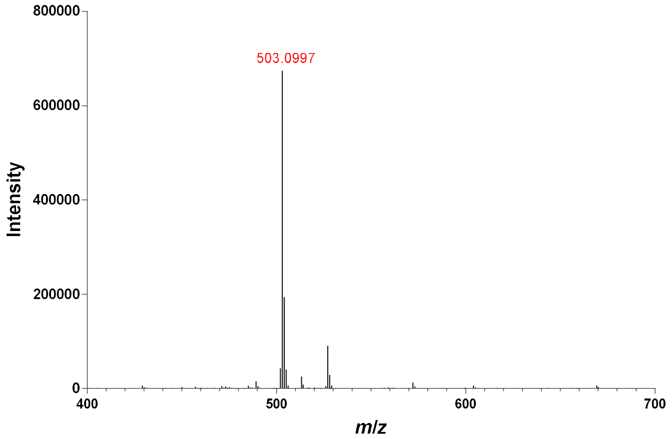

Figure S8. MS spectrum of **2** obtained by AJS-Q-TOF *m/z*: 503.0997 $[M+H]^+$ (calcd. for $C_{27}H_{19}O_{10}$, 503.0978, Δ +3.8 ppm).

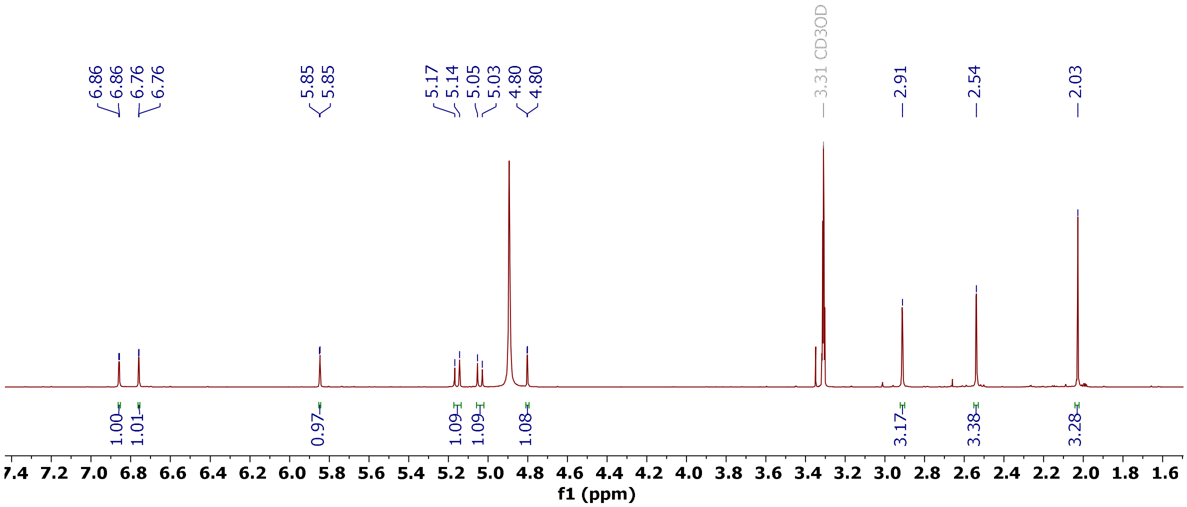

Figure S9. ^1H spectrum of **2** recorded in CD_3OD at 500 MHz.

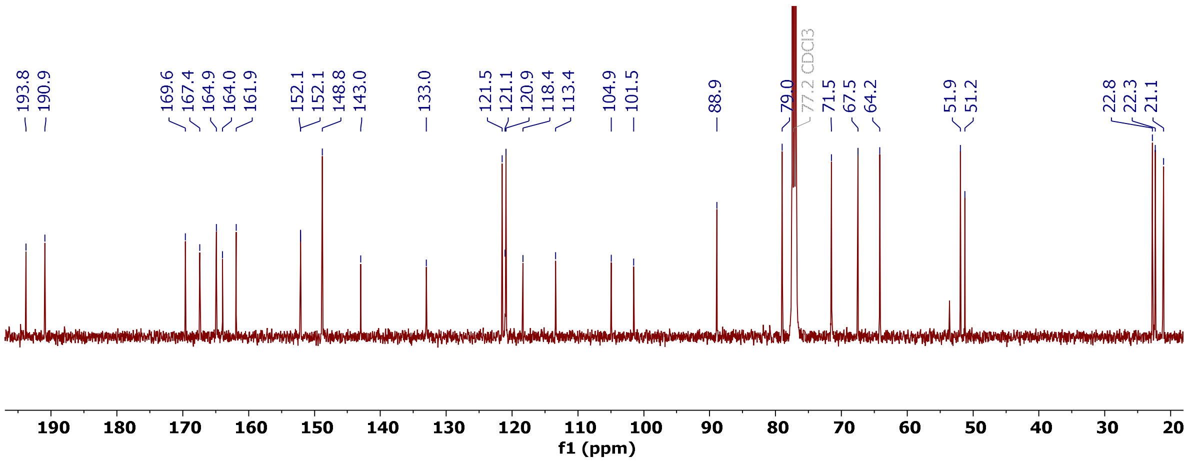

Figure S10. ^{13}C NMR spectrum of **2** recorded in CD_3OD at 125 MHz.

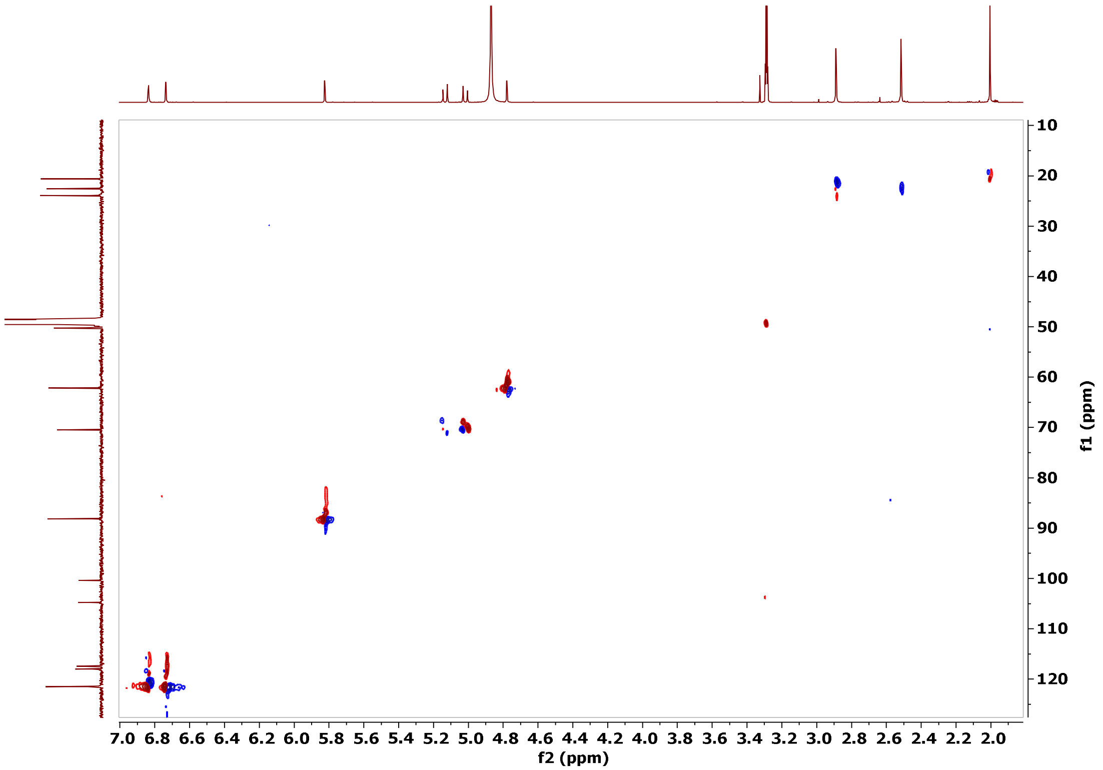

Figure S11. HSQC NMR spectrum of **2** (500 MHz, CD_3OD).

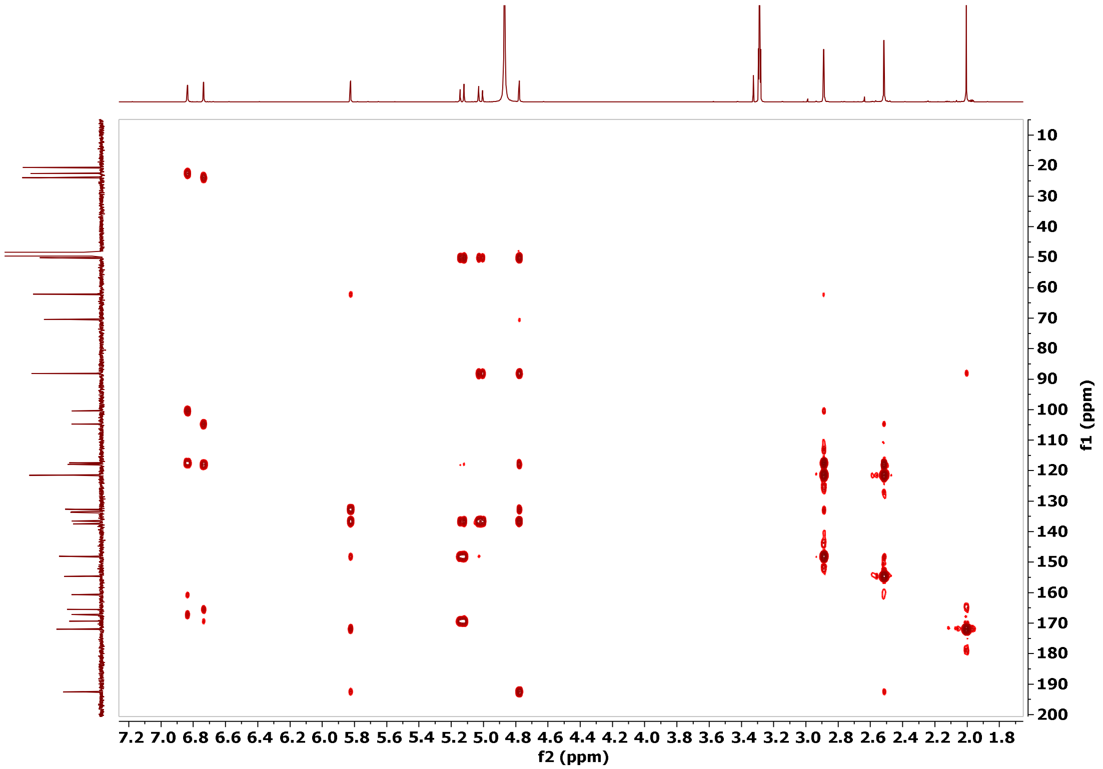

Figure S12. HMBC NMR spectrum of **2** (500 MHz, CD_3OD).

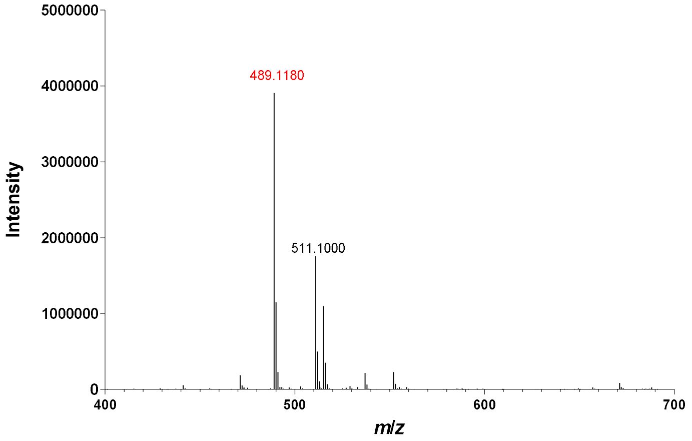

Figure S13. MS spectrum of **3** obtained by AJS-Q-TOF *m/z*: 489.1180 $[M+H]^+$ (calcd. for $C_{27}H_{21}O_9$, 489.1186, Δ -1.2 ppm).

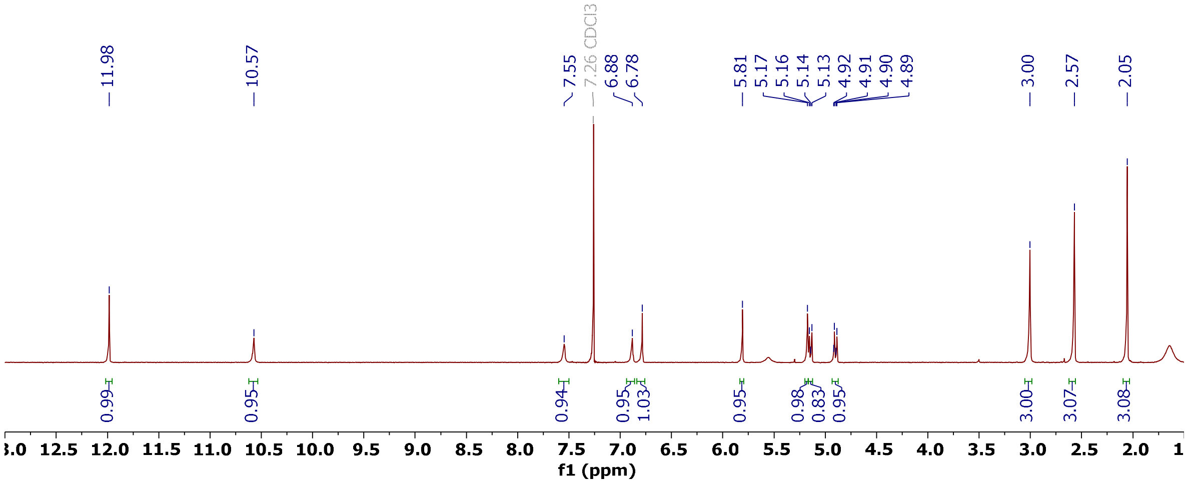

Figure S14. ^{1}H spectrum of **3** recorded in $CDCl_3$ at 500 MHz.

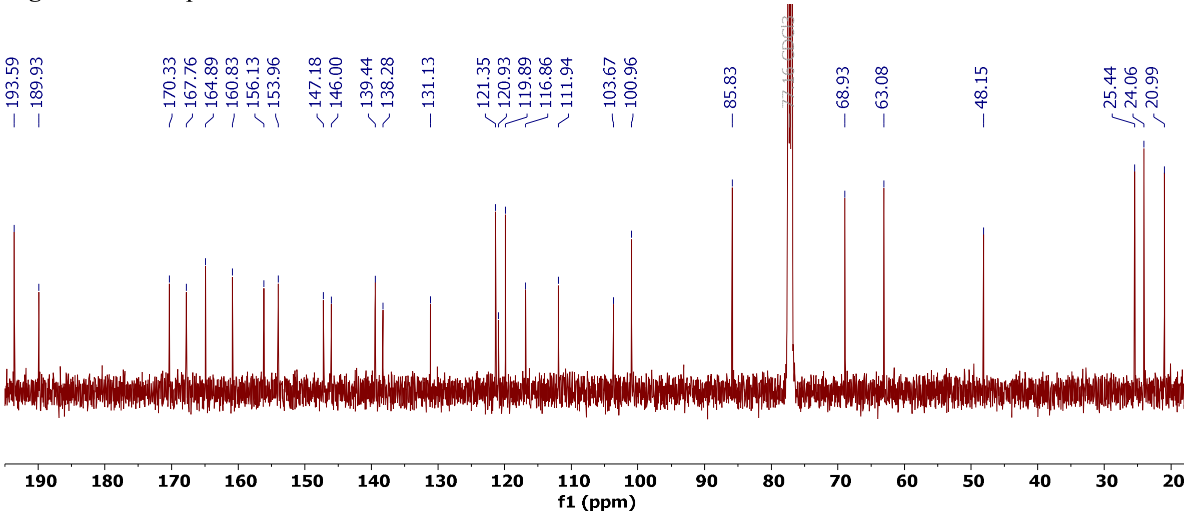

Figure S15. ^{1}H spectrum of **3** recorded in $CDCl_3$ at 125 MHz.

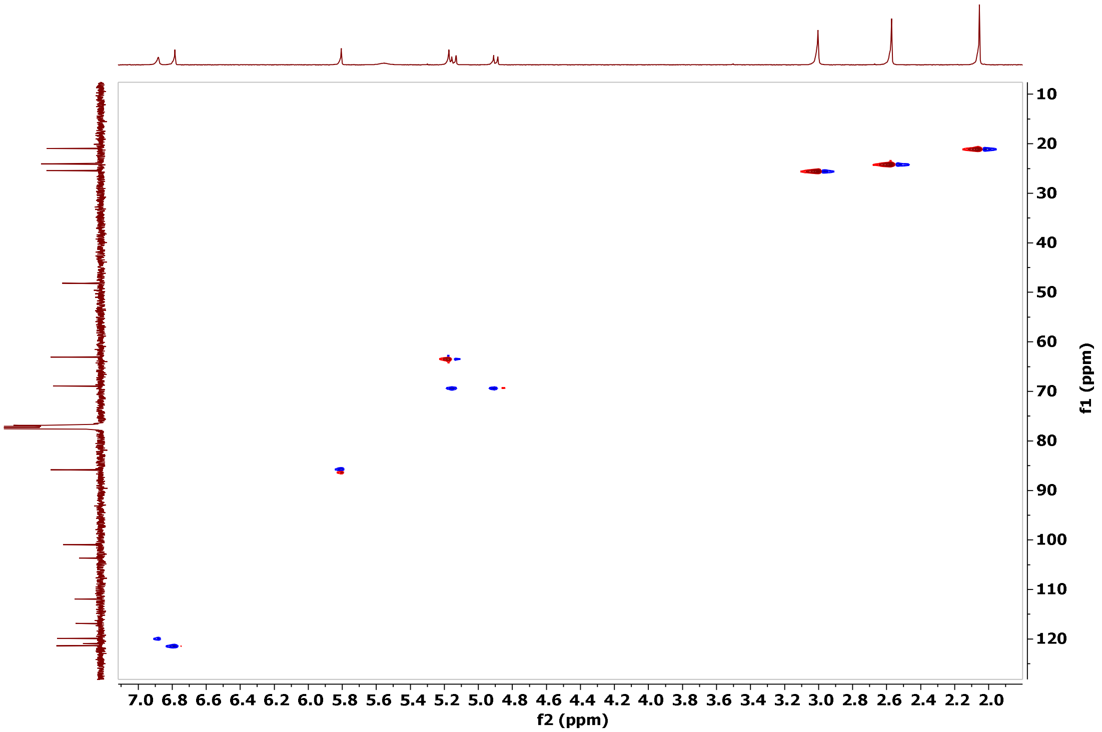

Figure S16. HSQC NMR spectrum of **3** (500 MHz, $CDCl_3$).

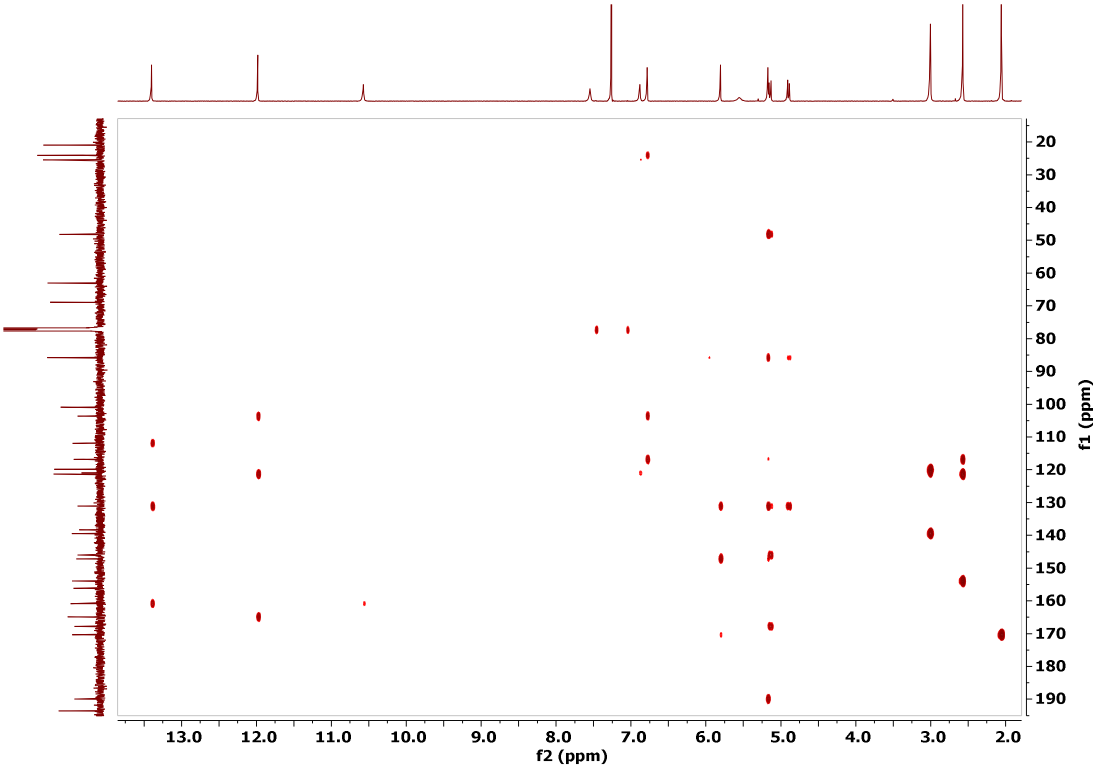

Figure S17. HMBC NMR spectrum of **3** (500 MHz, $CDCl_3$).

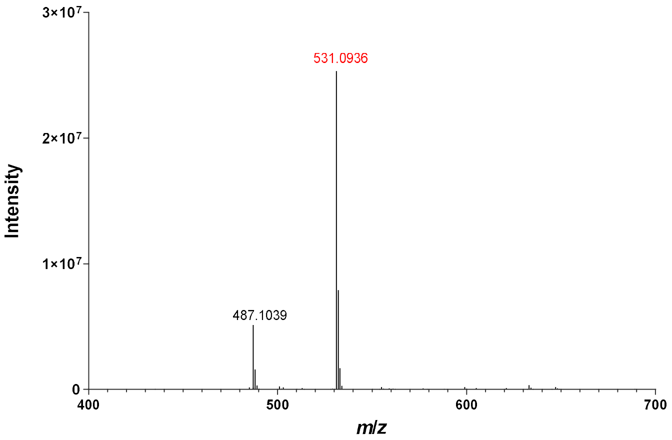

Figure S18. MS spectrum of **4** obtained by AJS-Q-TOF *m/z*: 531.0936 $[M+H]^+$ (calcd. for $C_{28}H_{19}O_{11}$, 531.0927, Δ +1.7 ppm).

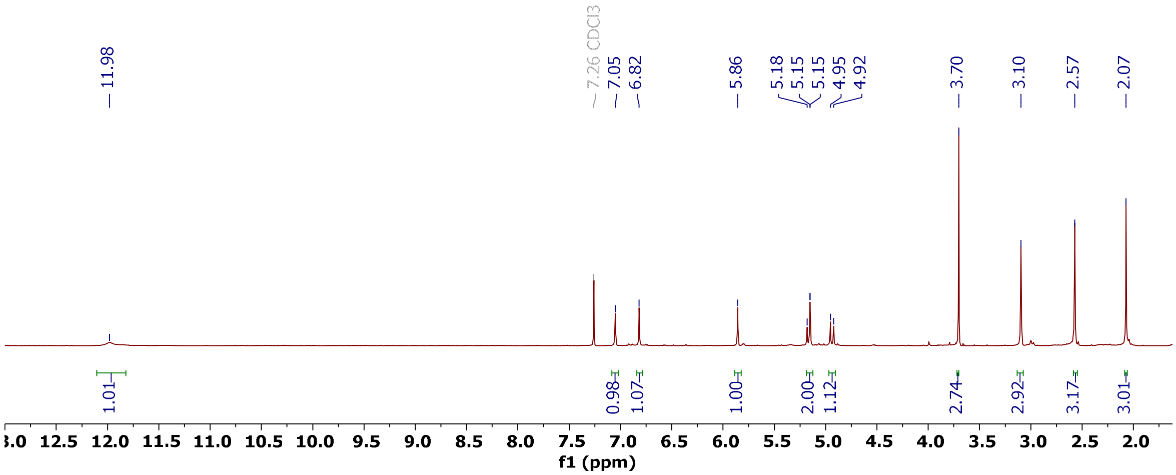

Figure S19. ^1H spectrum of **4** recorded in $CDCl_3$ at 400 MHz.

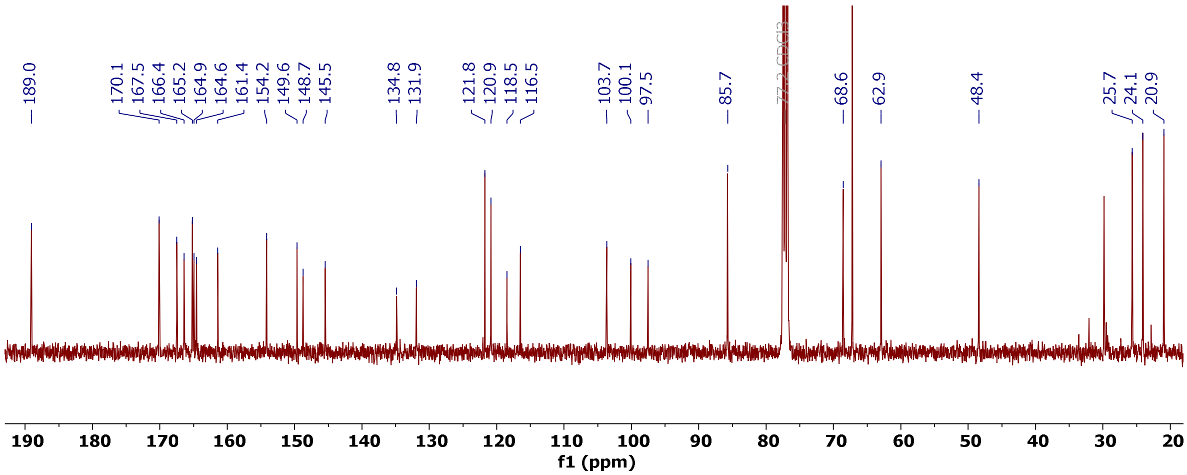

Figure S20. ^{13}C NMR spectrum of **4** recorded in $CDCl_3$ at 100 MHz.

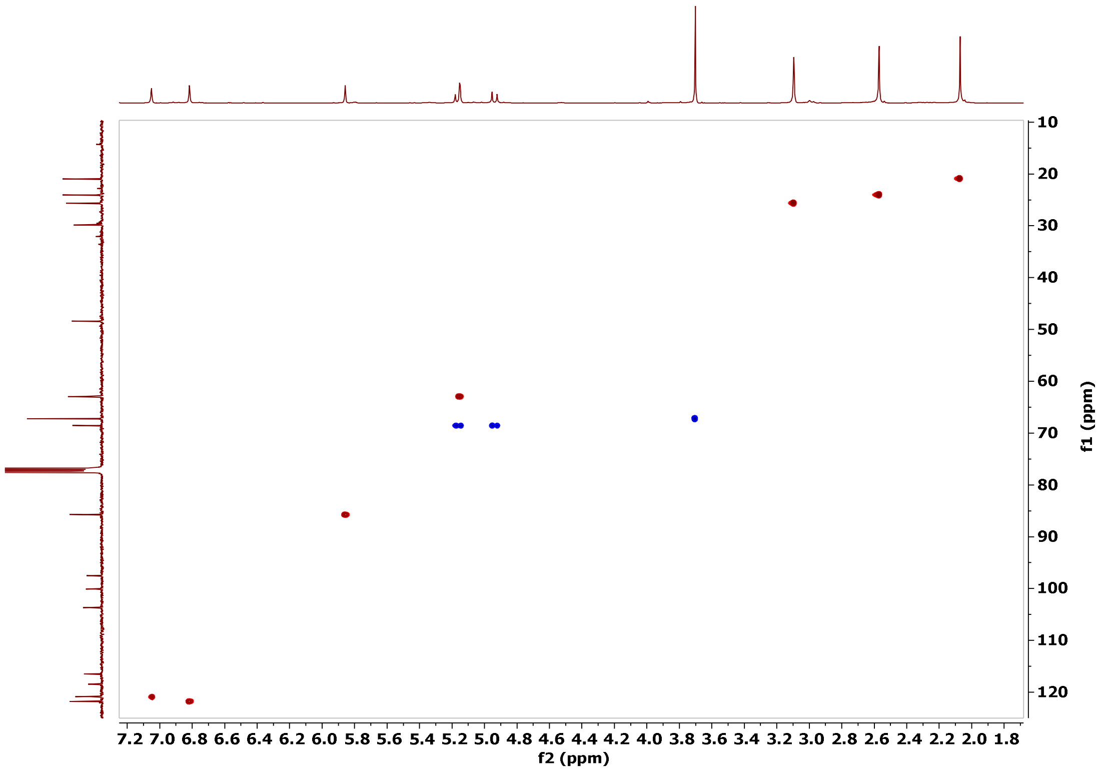

Figure S21. HSQC NMR spectrum of **4** (400 MHz, $CDCl_3$).

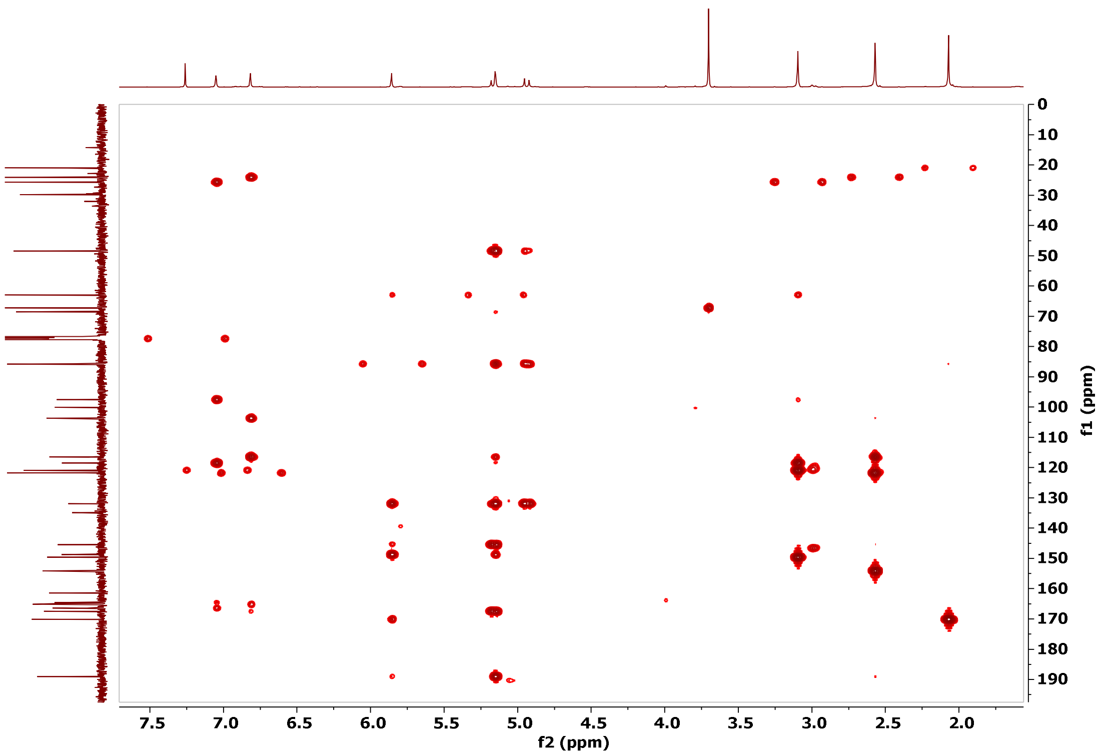

Figure S22. HMBC NMR spectrum of **4** (400 MHz, $CDCl_3$).

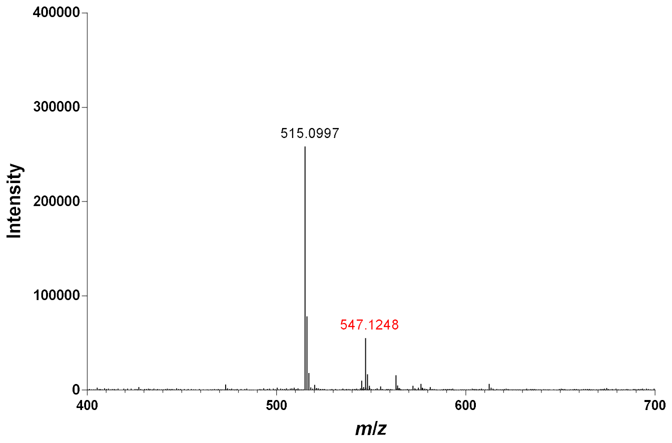

Figure S23. MS spectrum of **5** obtained by AJS-Q-TOF *m/z*: 547.1248 [M+H]$^+$ (calcd. for $C_{29}H_{23}O_{11}$, 547.1240, Δ +1.5 ppm).

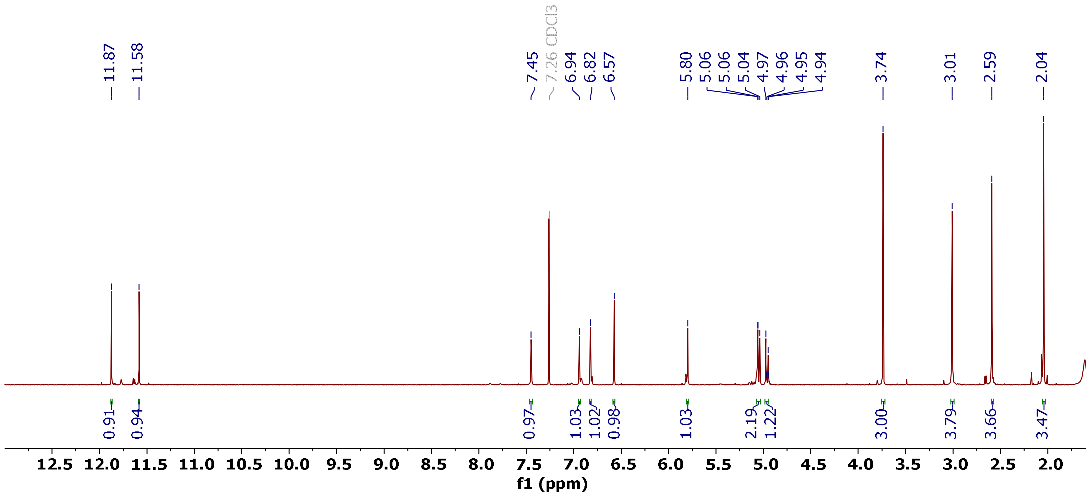

Figure S24. ^{1}H spectrum of **5** recorded in CDCl$_3$ at 500 MHz.

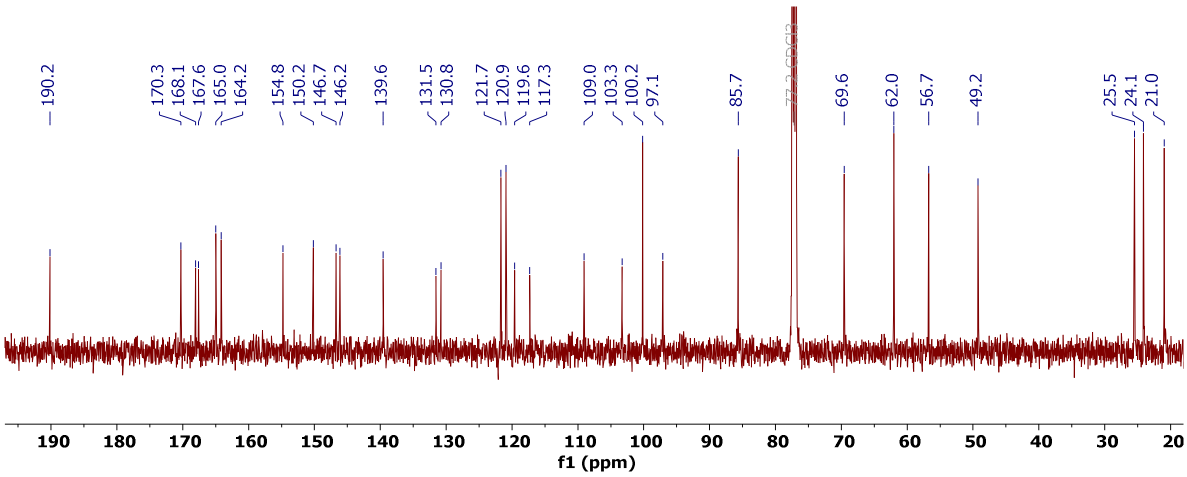

Figure S25. ^{13}C NMR spectrum of **5** recorded in CDCl$_3$ at 125 MHz.

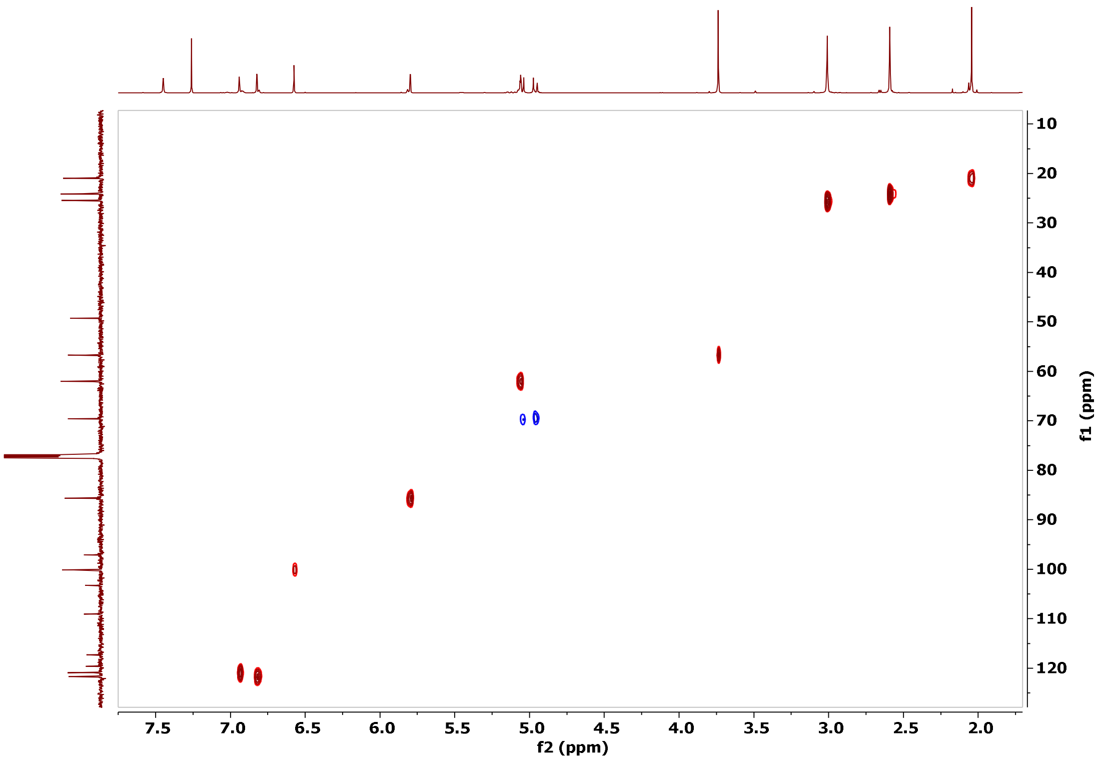

Figure S26. HSQC NMR spectrum of **5** (500 MHz, $CDCl_3$).

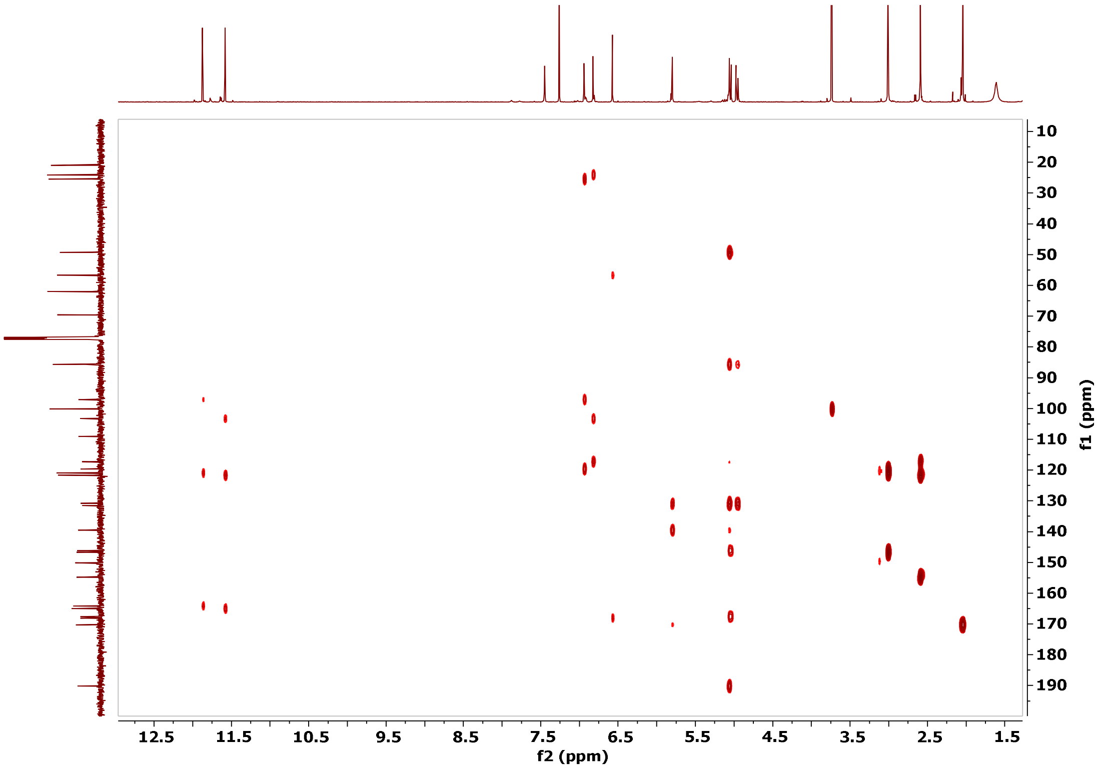

Figure S27. HMBC NMR spectrum of **5** (500 MHz, $CDCl_3$).

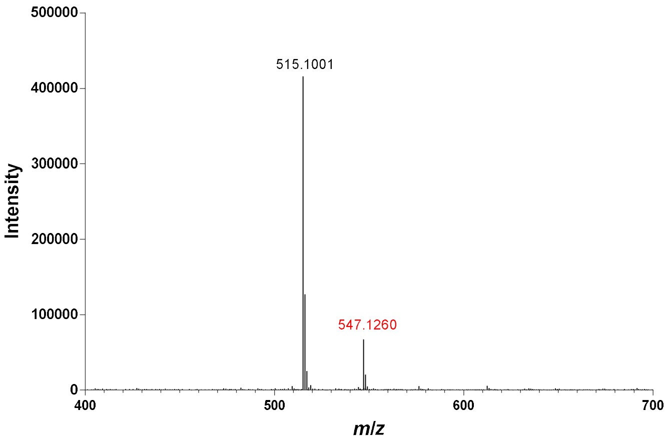

Figure S28. MS spectrum of **6** obtained by AJS-Q-TOF *m*/*z*: 547.1260 $[M+H]^+$ (calcd. for $C_{29}H_{23}O_{11}$, 547.1240, Δ +3.7 ppm).

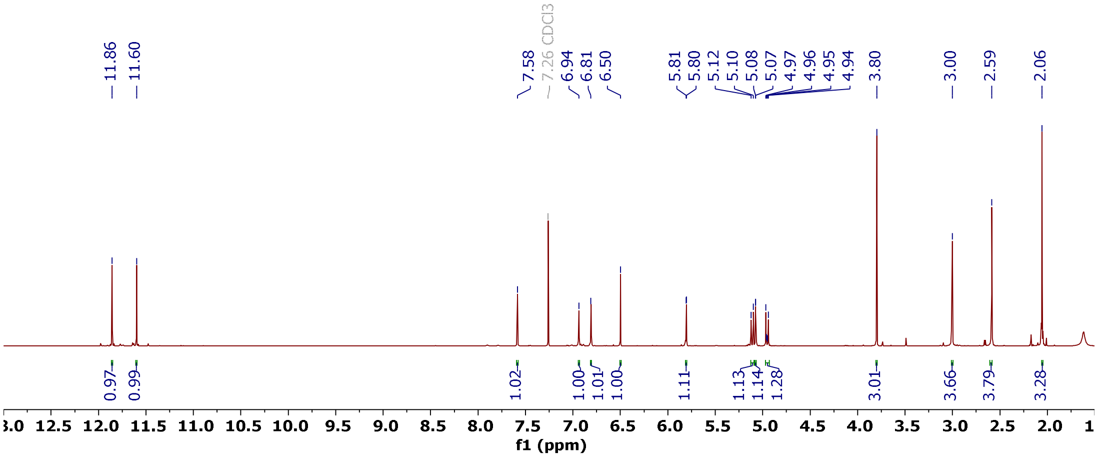

Figure S29. ^{1}H spectrum of **6** recorded in $CDCl_3$ at 500 MHz.

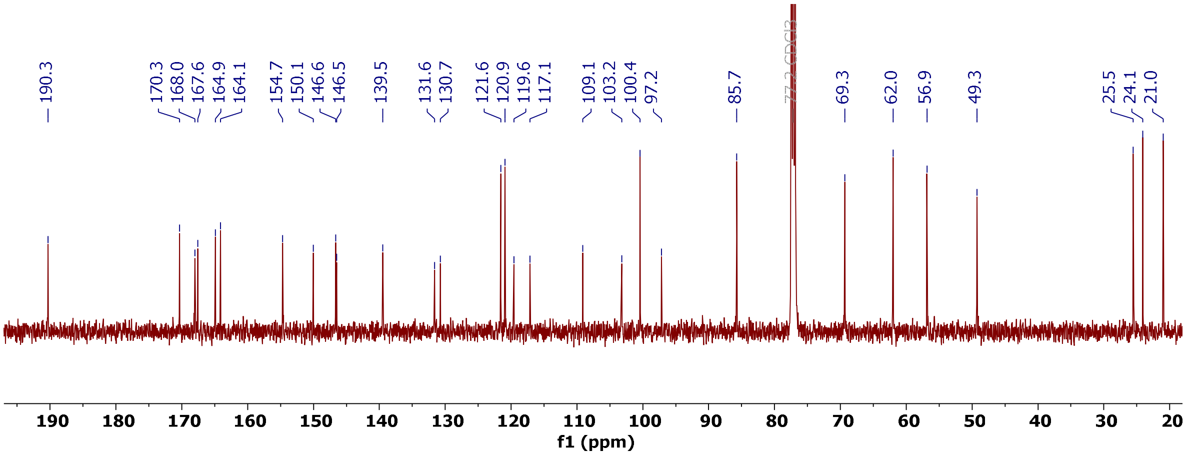

Figure S30. ^{13}C NMR spectrum of **6** recorded in $CDCl_3$ at 125 MHz.

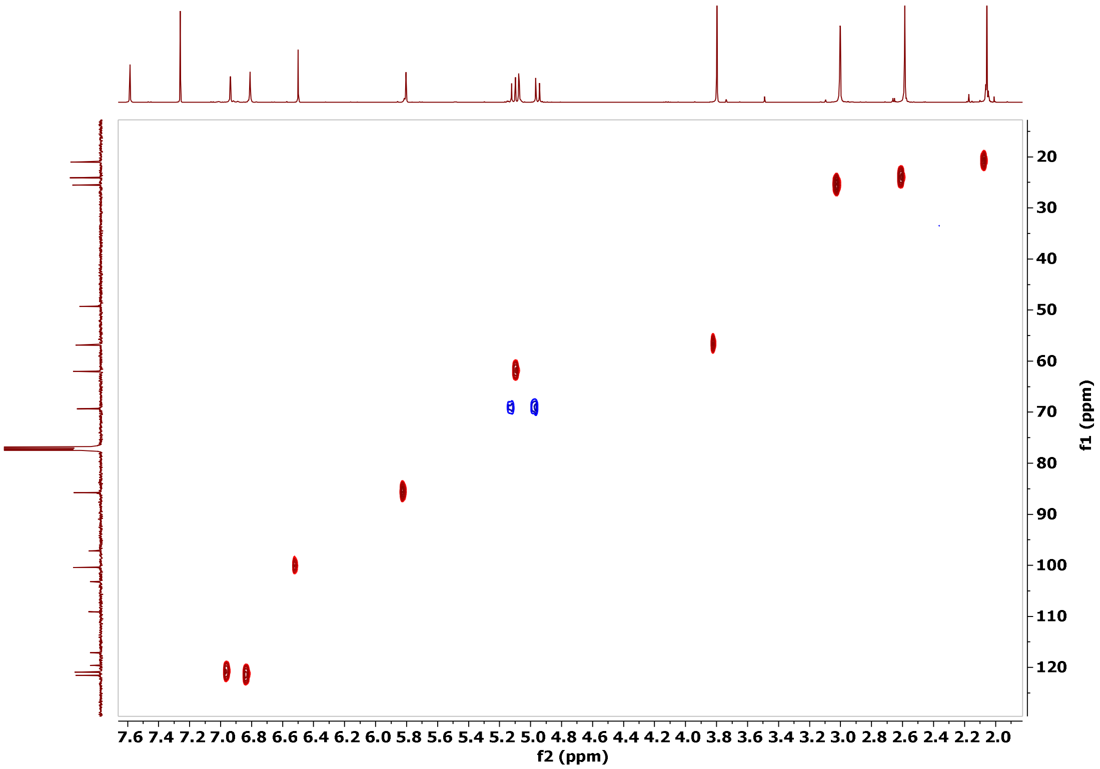

Figure S31. HSQC NMR spectrum of **6** (500 MHz, $CDCl_3$).

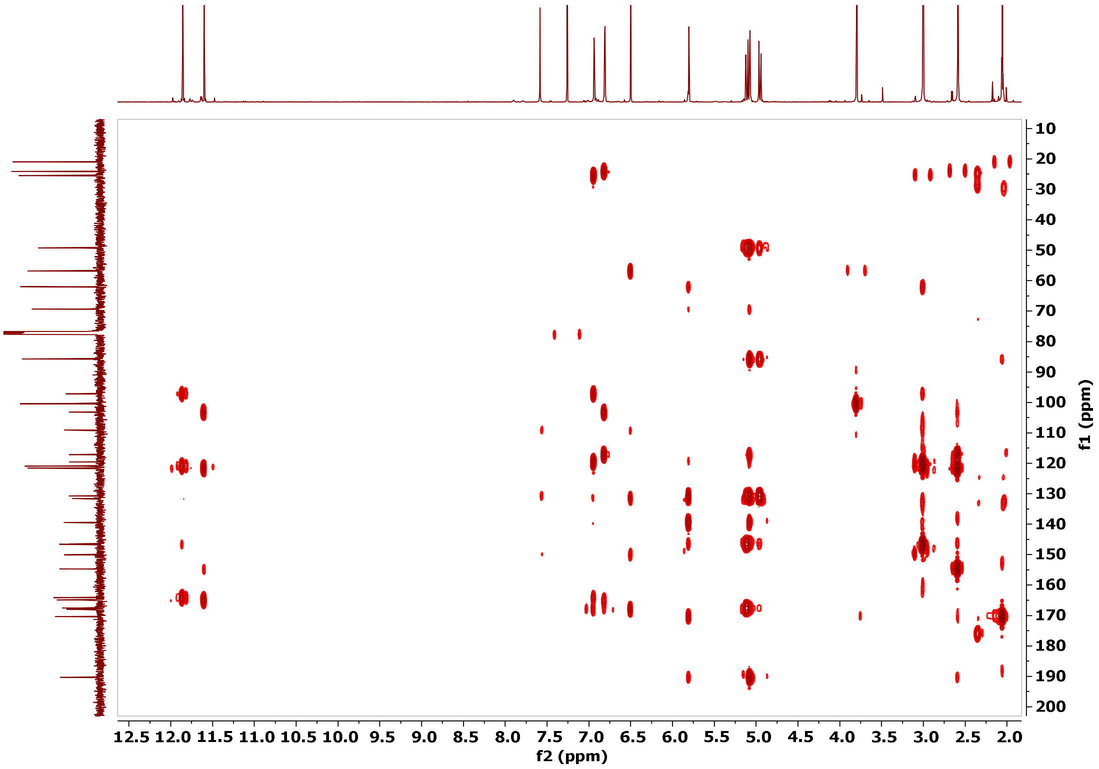

Figure S32. HMBC NMR spectrum of **6** (500 MHz, $CDCl_3$).

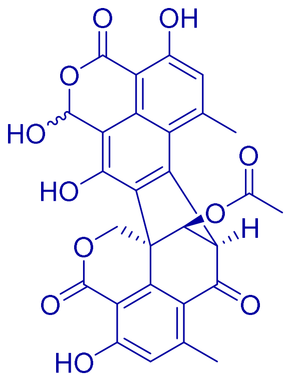

8/9

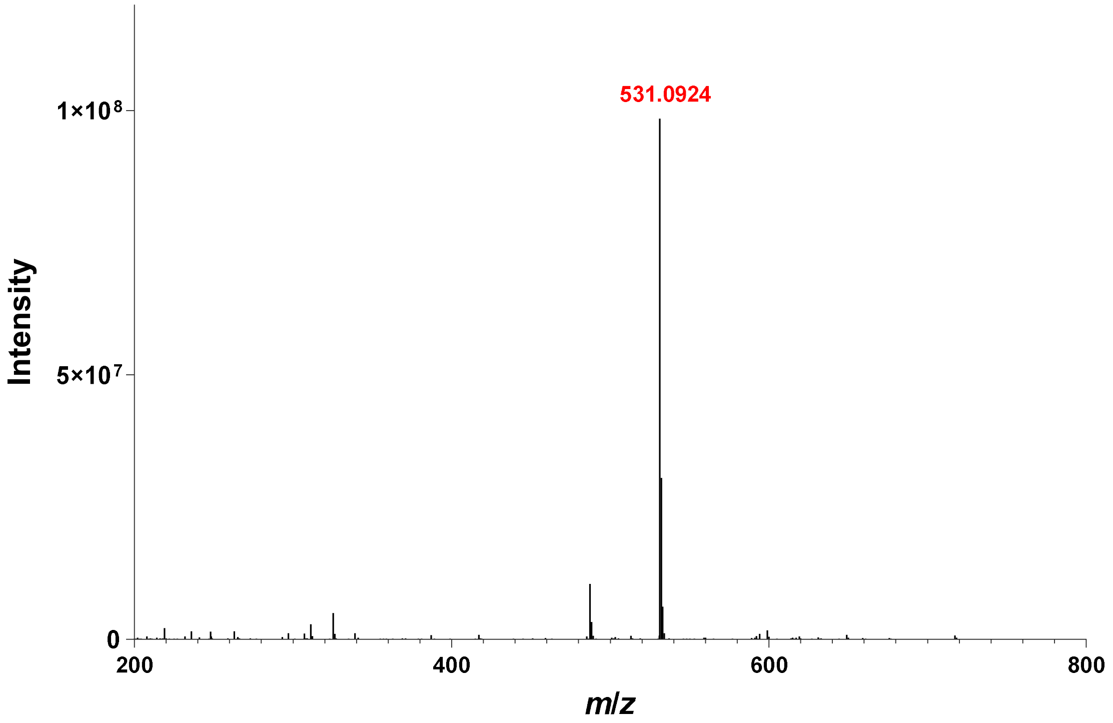

Figure S33. MS spectrum of **8**/**9** obtained by Q-Exactive *m*/*z*: 531.0924 [M-H]$^-$ (calcd. for $C_{28}H_{19}O_{11}$, 531.0927, Δ -0.6 ppm).

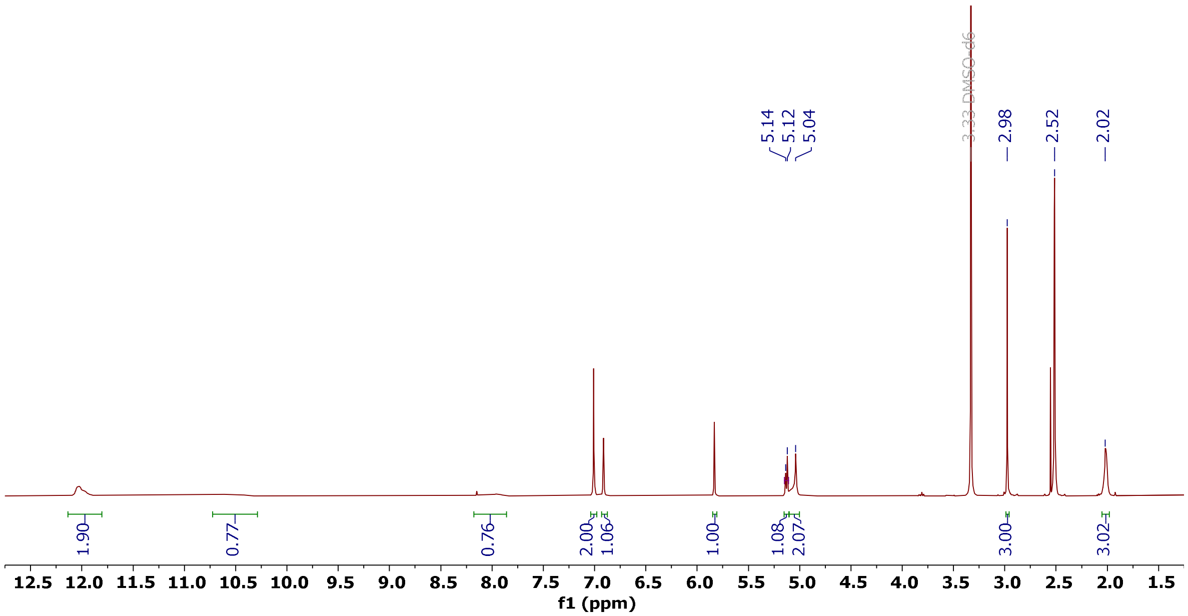

Figure S34. ^{1}H-NMR spectrum of **8**/**9** recorded in $CDCl_3$ at 700 MHz.

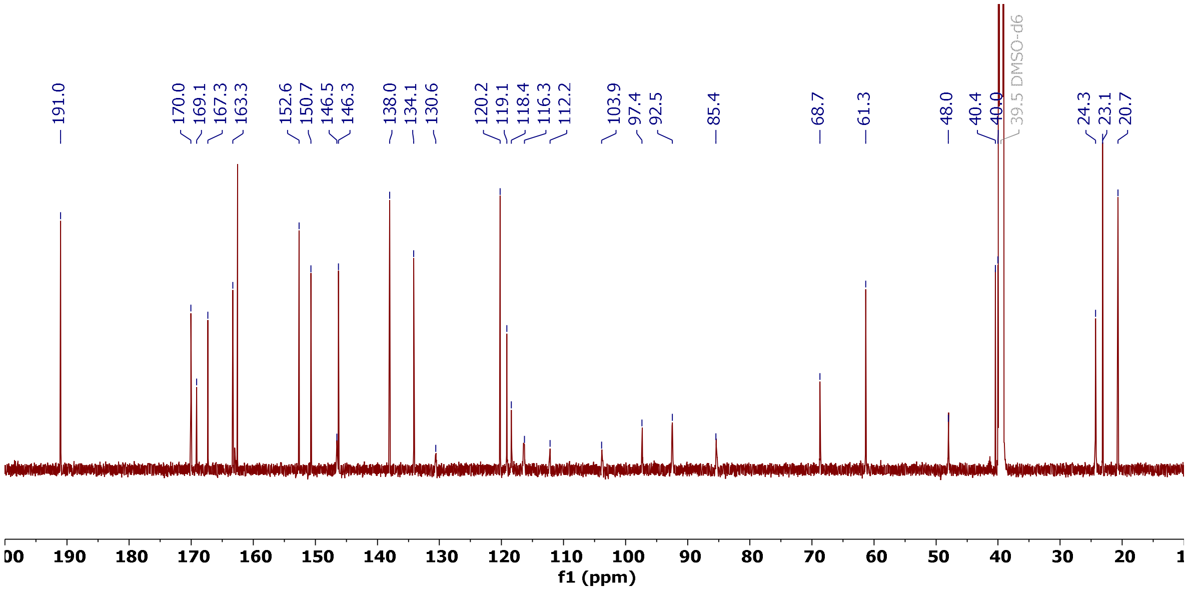

Figure S35. ^{13}C-NMR spectrum of **8**/**9** recorded in $CDCl_3$ at 175 MHz.

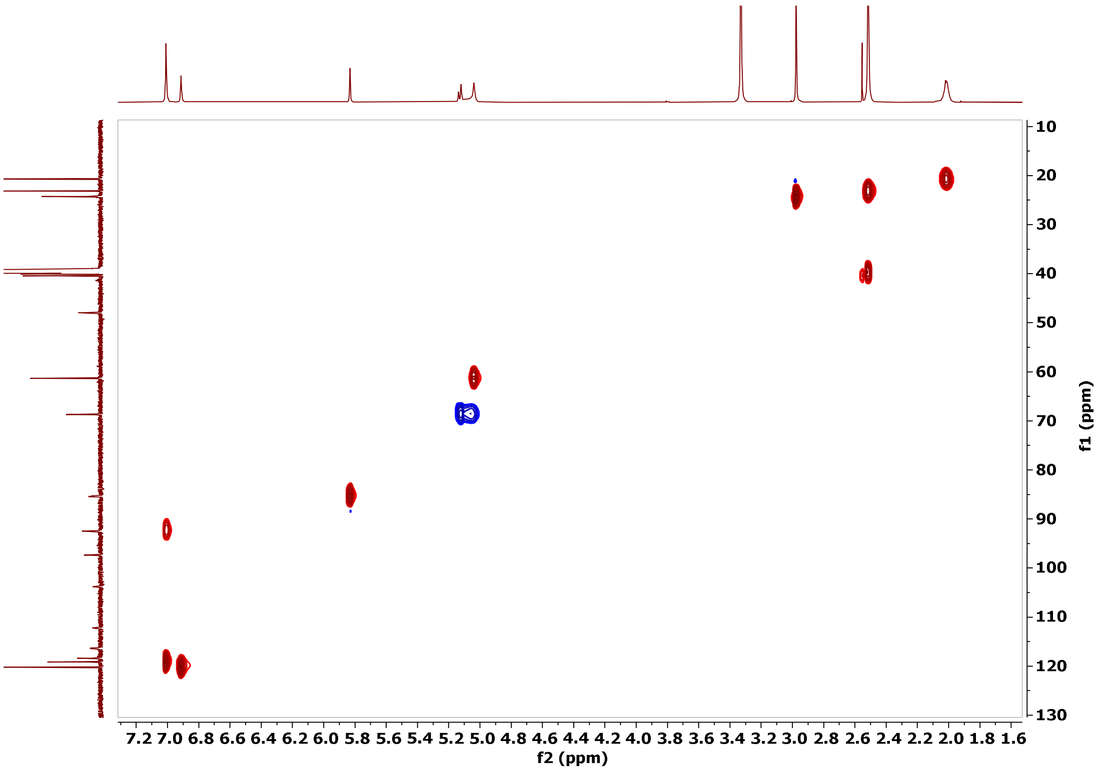

Figure S36. HSQC NMR spectrum of **8**/**9** recorded in $CDCl_3$ at 700 MHz.

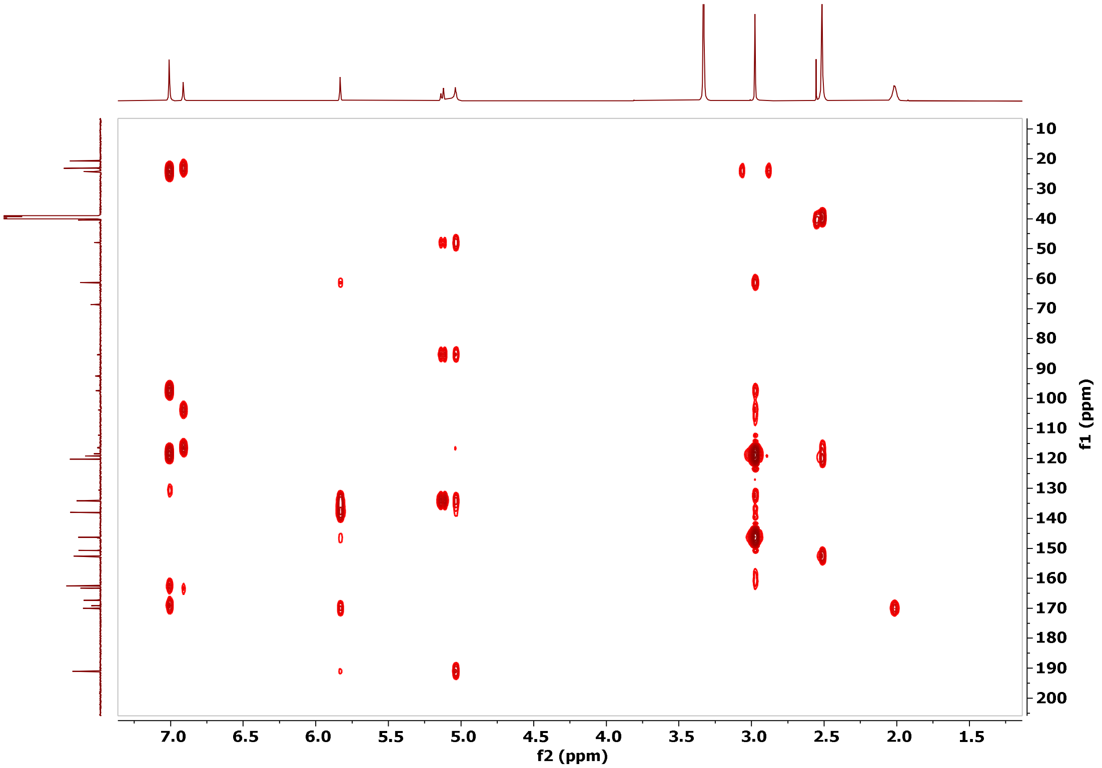

Figure S37. HMBC NMR spectrum of **8**/**9** recorded in $CDCl_3$ at 700 MHz.

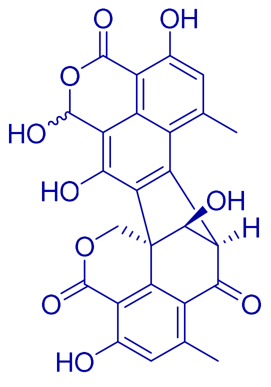

12/13

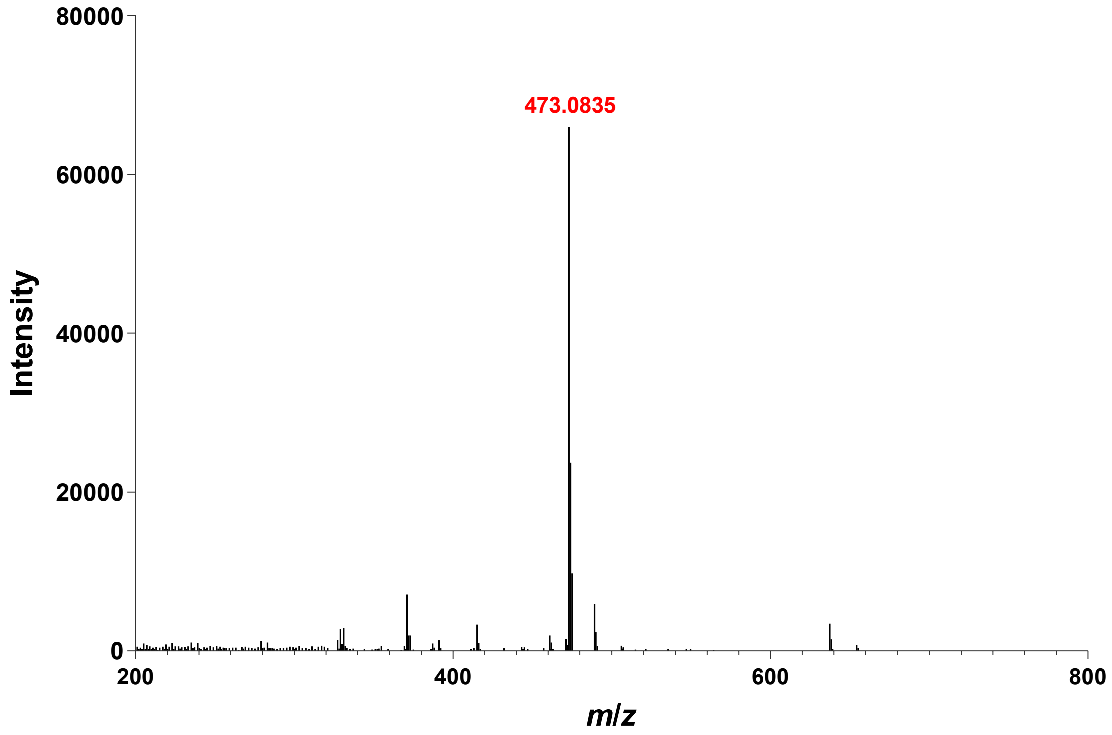

Figure S38. MS spectrum of **12/13** obtained by DART *m/z*: 473.0835 $[M+H^{+}-H_2O]^{+}$ (calcd. for $C_{26}H_{17}O_9$, 473.0873, Δ -8.0 ppm).

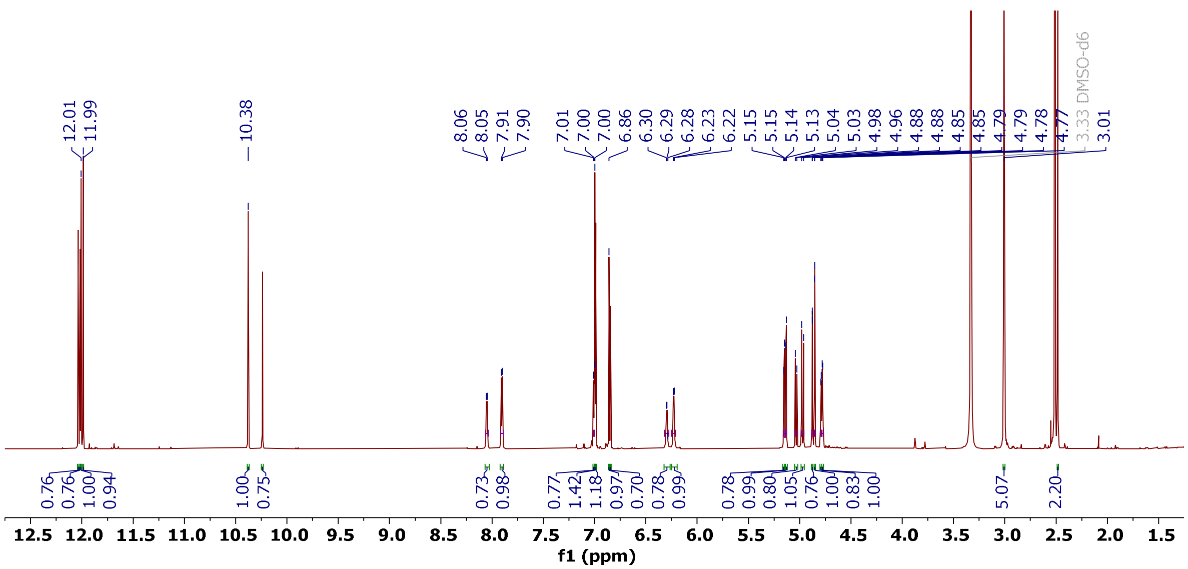

Figure S39. ^{1}H-NMR spectrum of **12**/**13** recorded in $CDCl_3$ at 700 MHz.

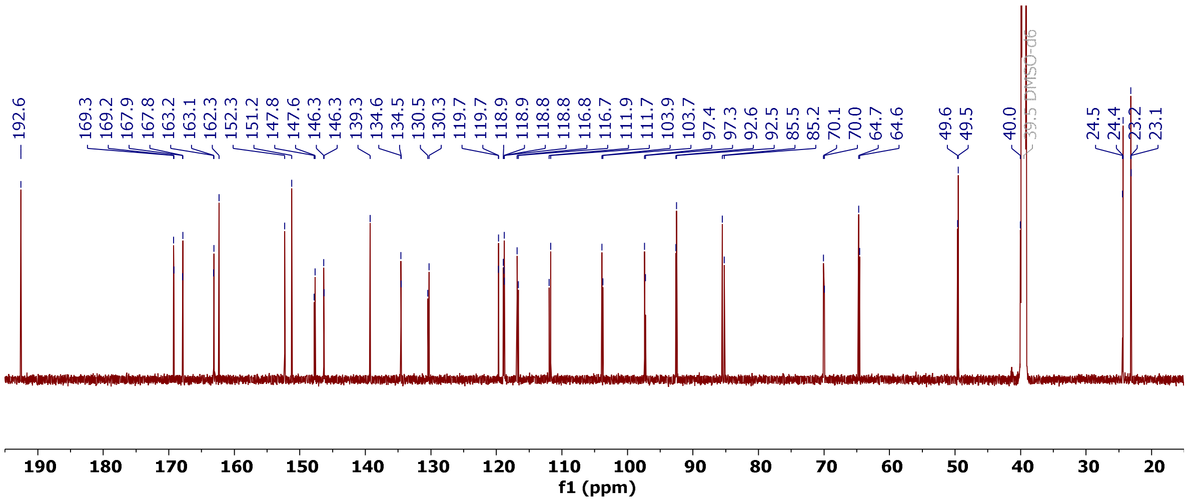

Figure S40. ^{13}C-NMR spectrum of **12**/**13** recorded in $CDCl_3$ at 175 MHz.

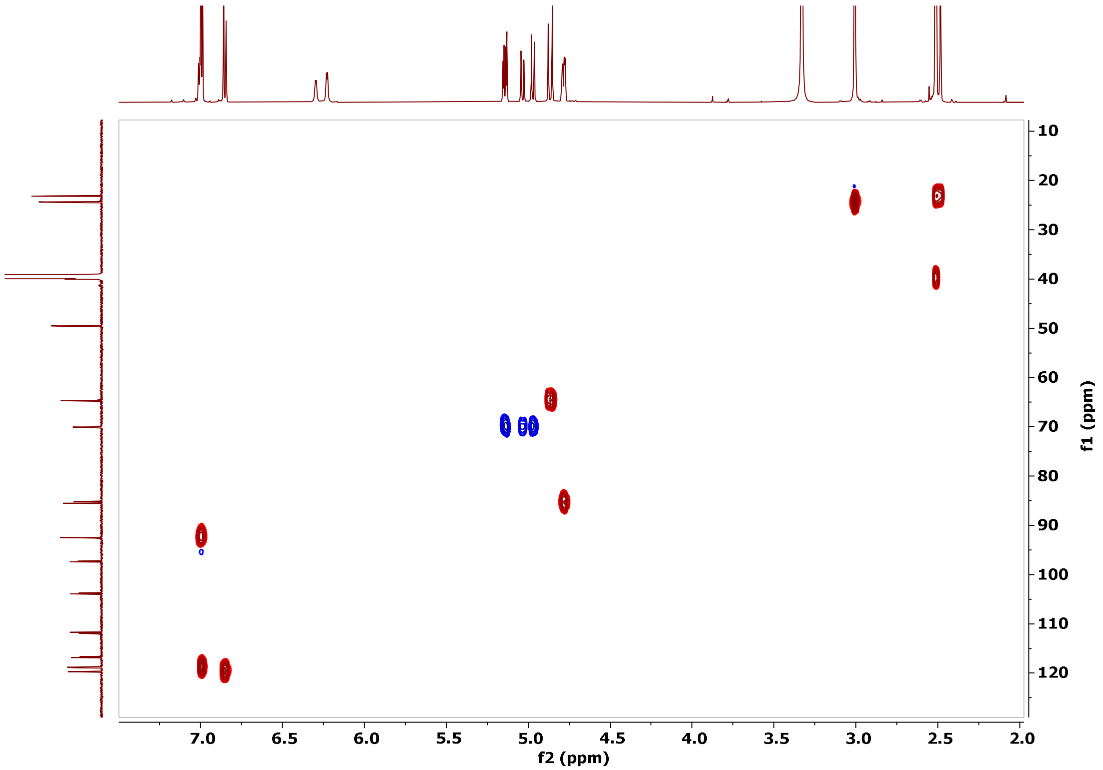

Figure S41. HSQC NMR spectrum of **12**/**13** recorded in $CDCl_3$ at 700 MHz.

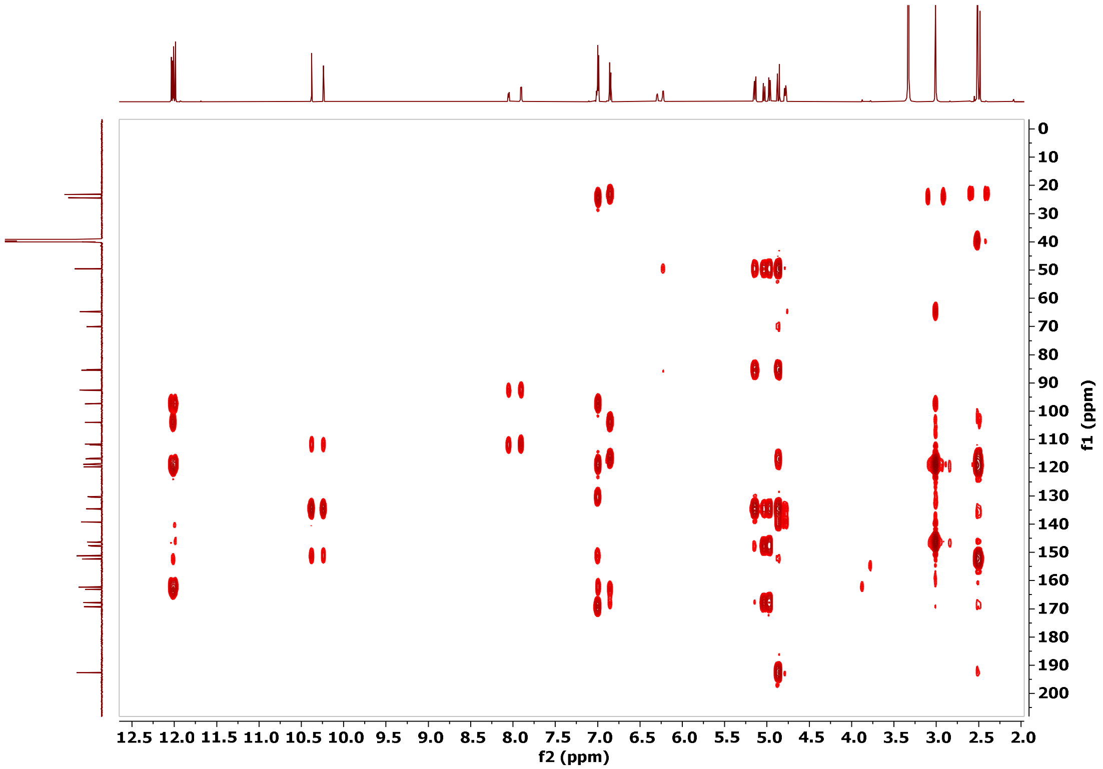

Figure S42. HMBC NMR spectrum of **12**/**13** recorded in $CDCl_3$ at 700 MHz.

Table S3. Spectroscopic data of ^{13}C-NMR and ^{1}H-NMR for **8**/**9** and **12**/**13**, recorded in $CDCl_3$, at 175 MHz and 700 MHz, respectively.

Position	δ_C	δ_H, (J in Hz)	δ_C	δ_H, (J in Hz)
	**Bacillisporin J**		**Bacillisporin I**	
1	92.5	7.01, s	92.5	6.99, d (6.5)
3	169.1	-	169.2	-
3a	97.4	-	118.7	-
3b	130.6	-	130.2	-
4	162.5	-	162.3	-
5	119.1	7.01, s	118.9	7.00, s
6	146.3	-	146.3	-
6a	118.4	-	97.4	-
7	138.0	-	139.3	-
8	134.1	-	134.4	-
9	150.7	-	151.2	-
9a	112.2	-	111.7	-
10	24.3	2.98, s	24.4	3.01, s
1′α	68.7	5.04, brs.	70.1	4.97, d (12.4)
1′β		5.13, d (12.3)		5.14, d (12.4)
3′	167.3	-	167.7	-
3′a	103.9	-	103.7	-
3′b	146.5	-	147.6	-
4′	163.3	-	163.1	-
5′	120.2	6.91, s	119.7	6.86, s
6′	152.6	-	151.9	-
6′a	116.3	-	116.6	-
7′	191.0	-	192.6	-
8′	61.3	5.04, s	64.7	4.85, d (1.1)
9′	85.4	5.83, s	85.5	4.78, d (4.0)
9′a	48.0	-	49.6	-
10′	23.1	2.52, s*	23.2	2.51, s*
<u>CH3</u>COO-9′	20.7	2.02, brs.	-	-
CH3<u>C</u>OO-9′	170.0	-	-	-
OH-1	-		-	7.90, d (6.5)
OH-4	-		-	11.99, s
OH-9	-		-	10.38, s
OH-4′	-		-	12.01, s

*This signal was assigned based on the contour observed in the HSQC experiment, as it overlaps with the solvent.

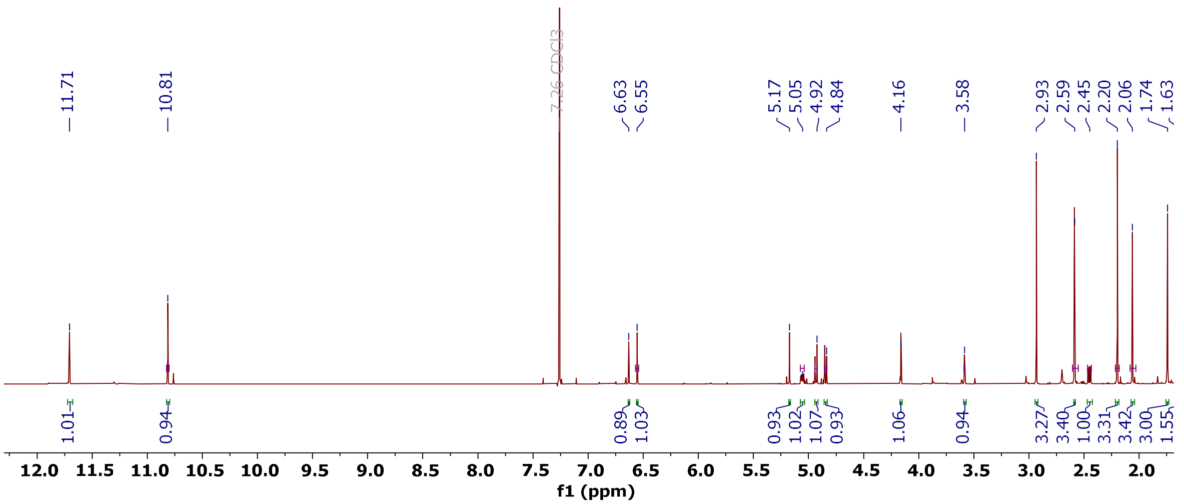

Figure S43. ^{1}H-NMR spectrum of **38** recorded in $CDCl_3$ at 700 MHz.

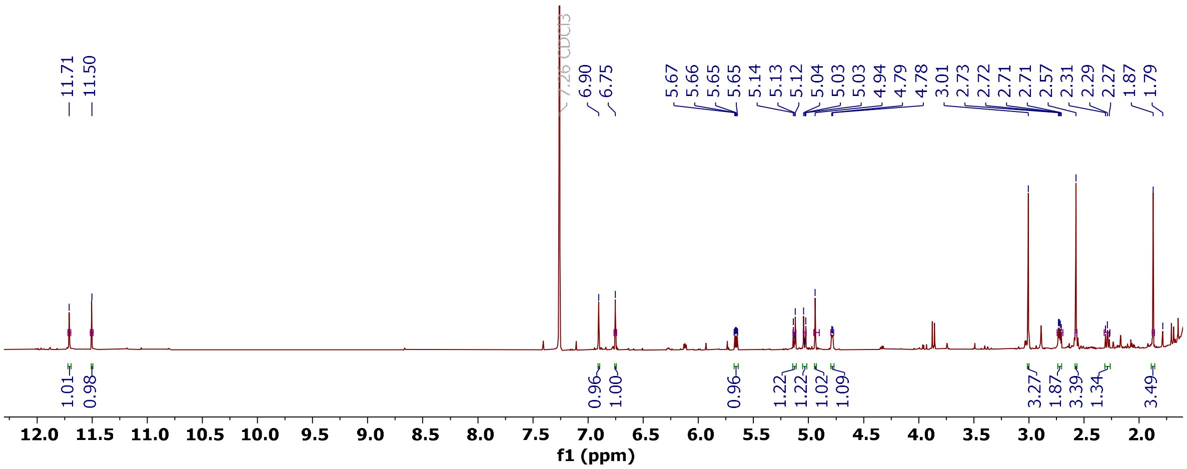

Figure S44. ^{1}H-NMR spectrum of **38a** recorded in $CDCl_3$ at 700 MHz.

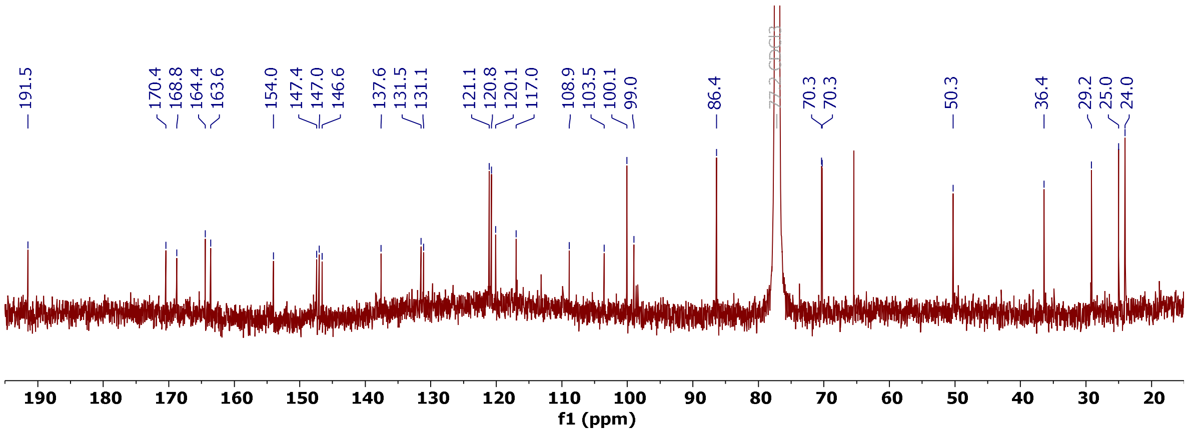

Figure S45. ^{13}C-NMR spectrum of **38a** recorded in $CDCl_3$ at 175 MHz.

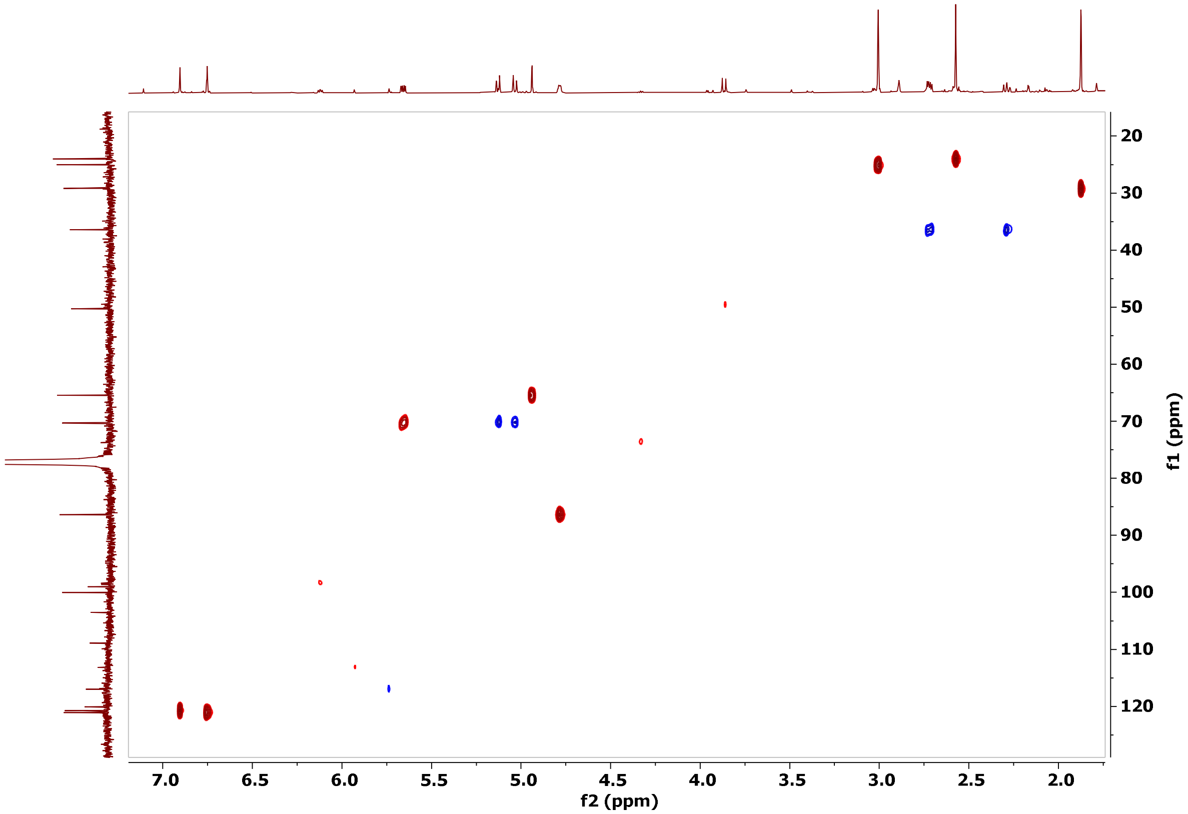

Figure S46. HSQC NMR spectrum of **38a** recorded in $CDCl_3$ at 700 MHz.

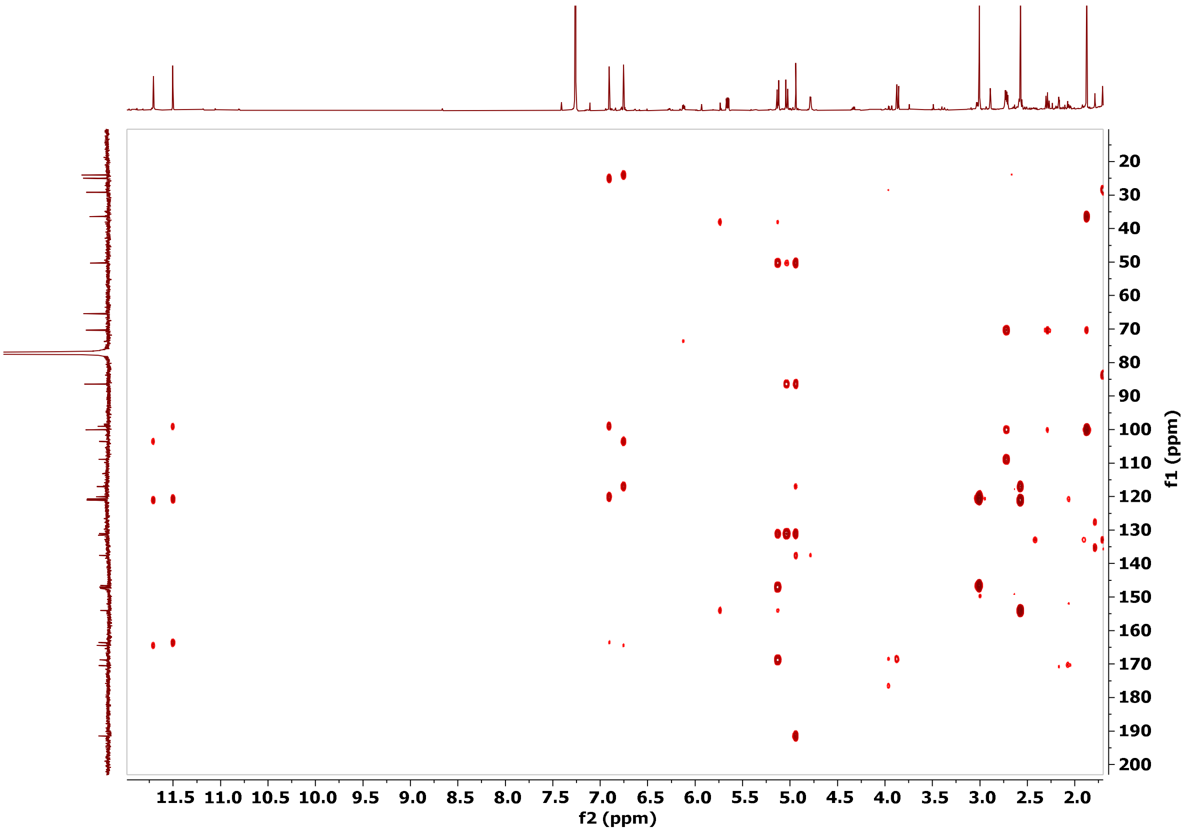

Figure S47. HMBC NMR spectrum of **38a** recorded in $CDCl_3$ at 700 MHz.

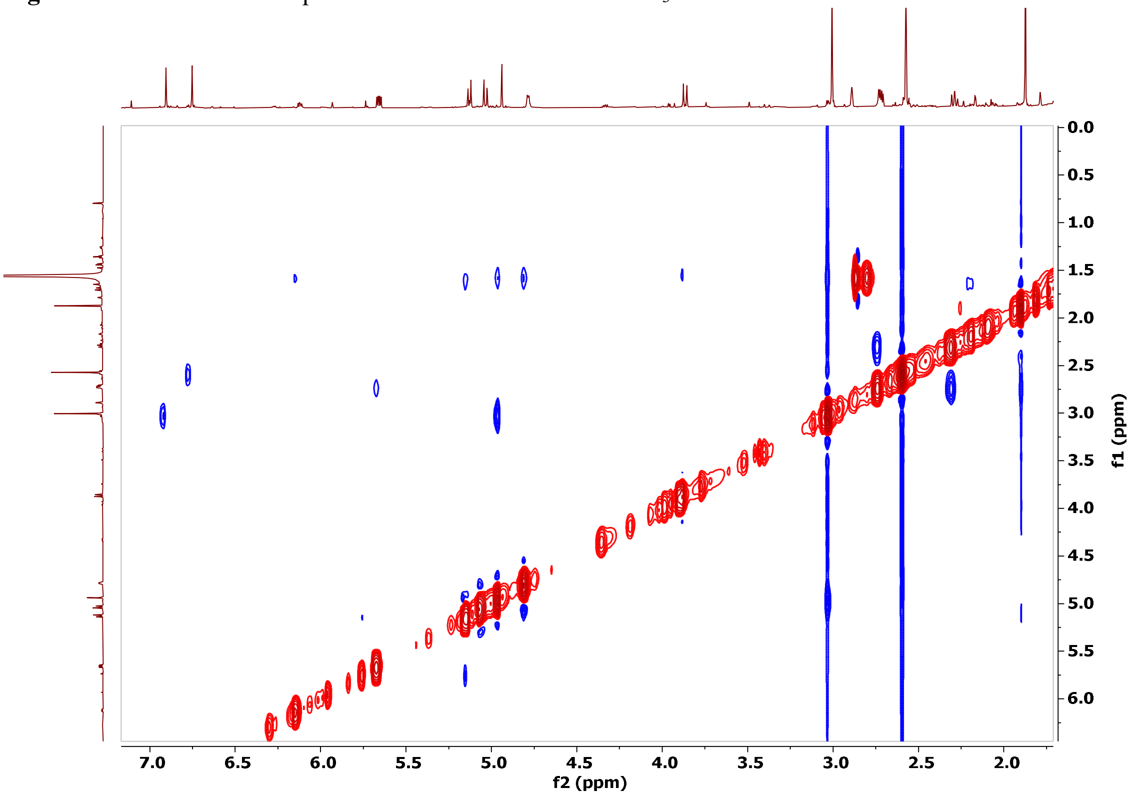

Figure S48. NOESY NMR spectrum of **38a** recorded in $CDCl_3$ at 700 MHz.

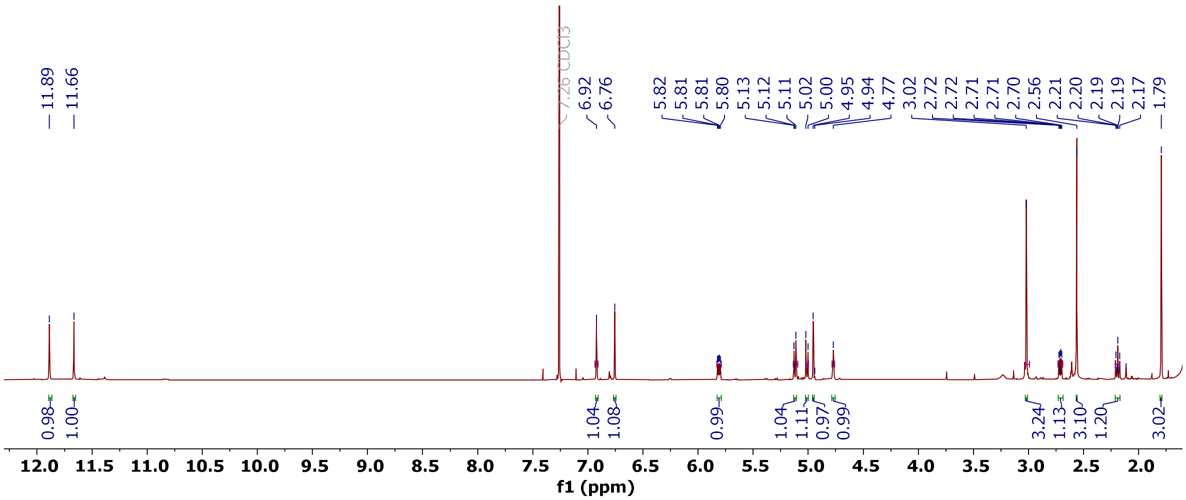

Figure S49. [13]H-NMR spectrum of **38b** recorded in $CDCl_3$ at 700 MHz.

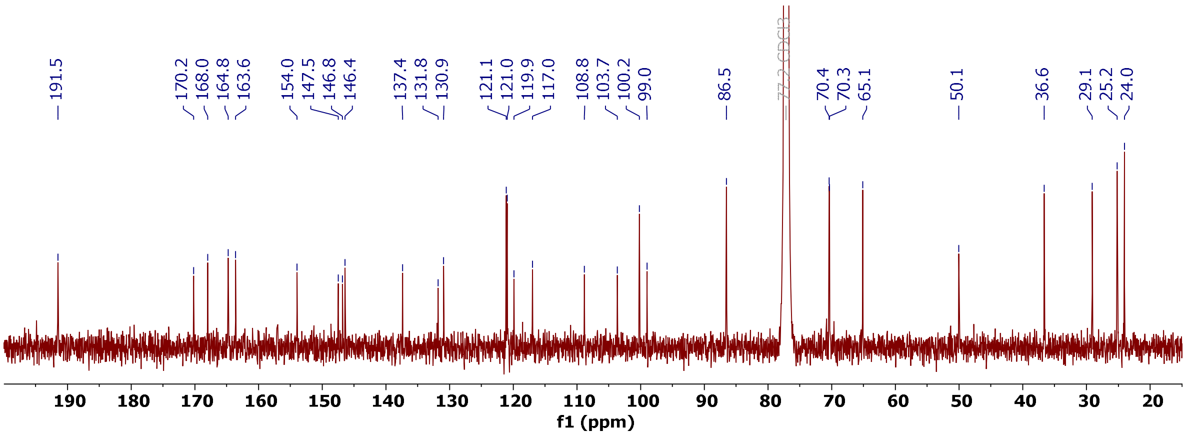

Figure S50. ^{13}C-NMR spectrum of **38b** recorded in $CDCl_3$ at 175 MHz.

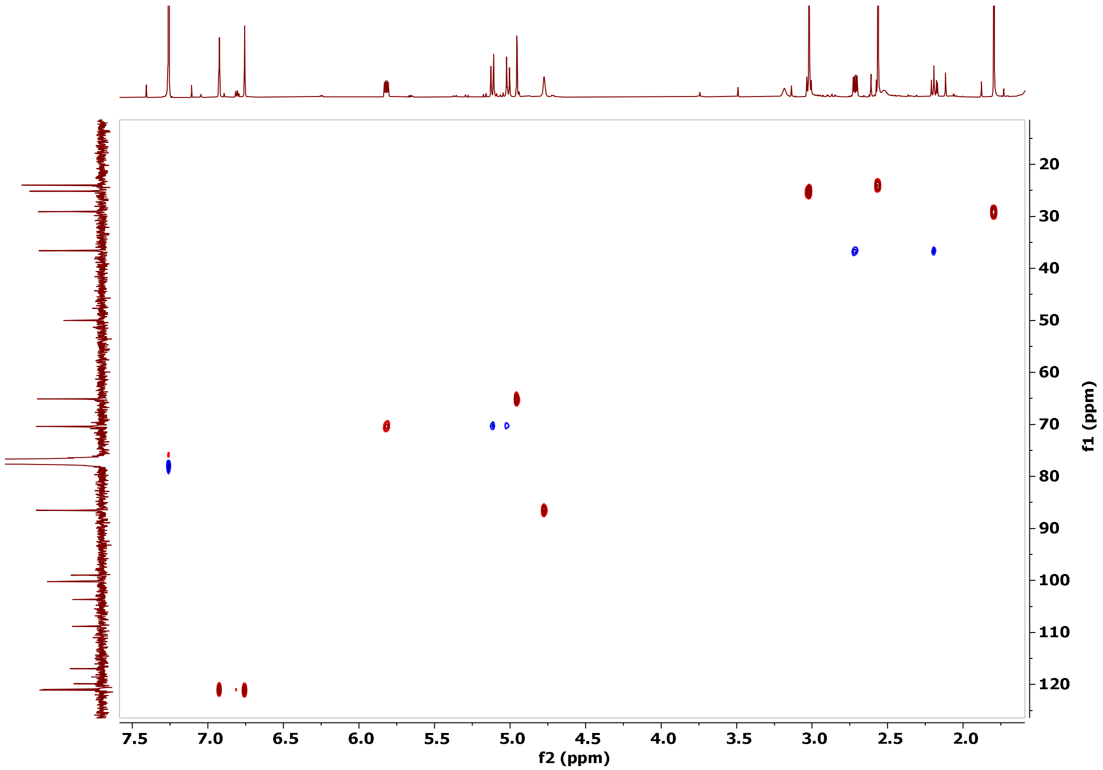

Figure S51. HSQC NMR spectrum of **38b** recorded in $CDCl_3$ at 700 MHz.

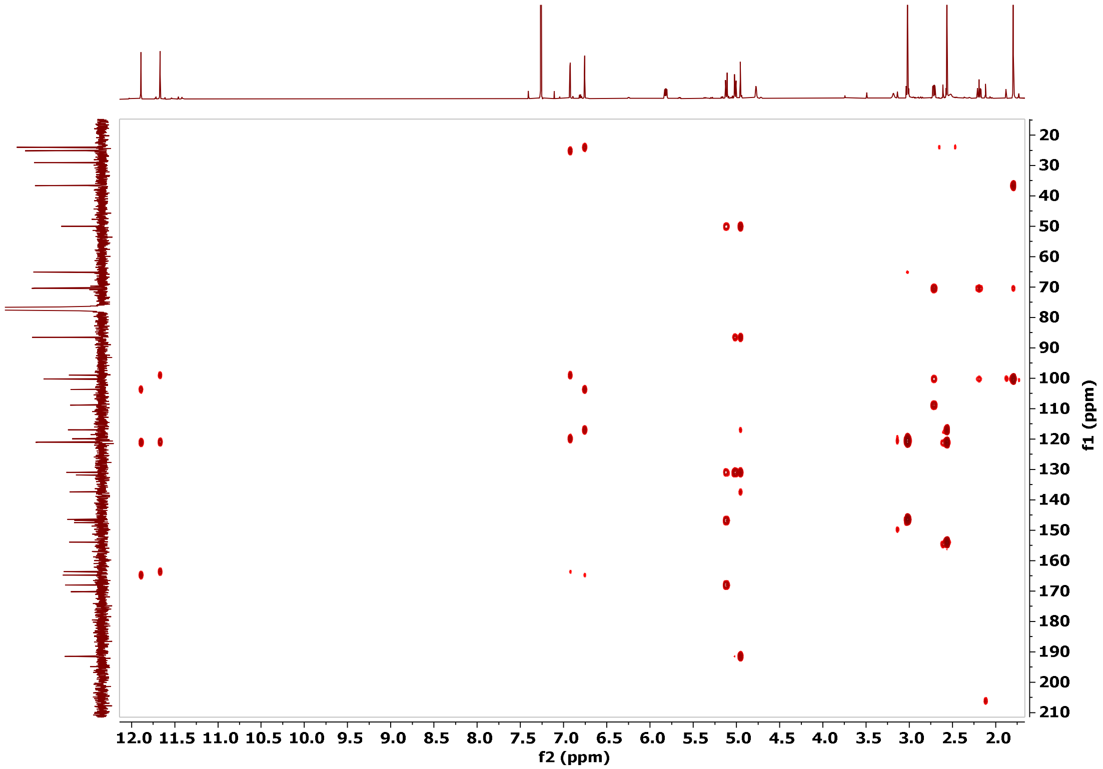

Figure S52. HMBC NMR spectrum of **38b** recorded in $CDCl_3$ at 700 MHz.

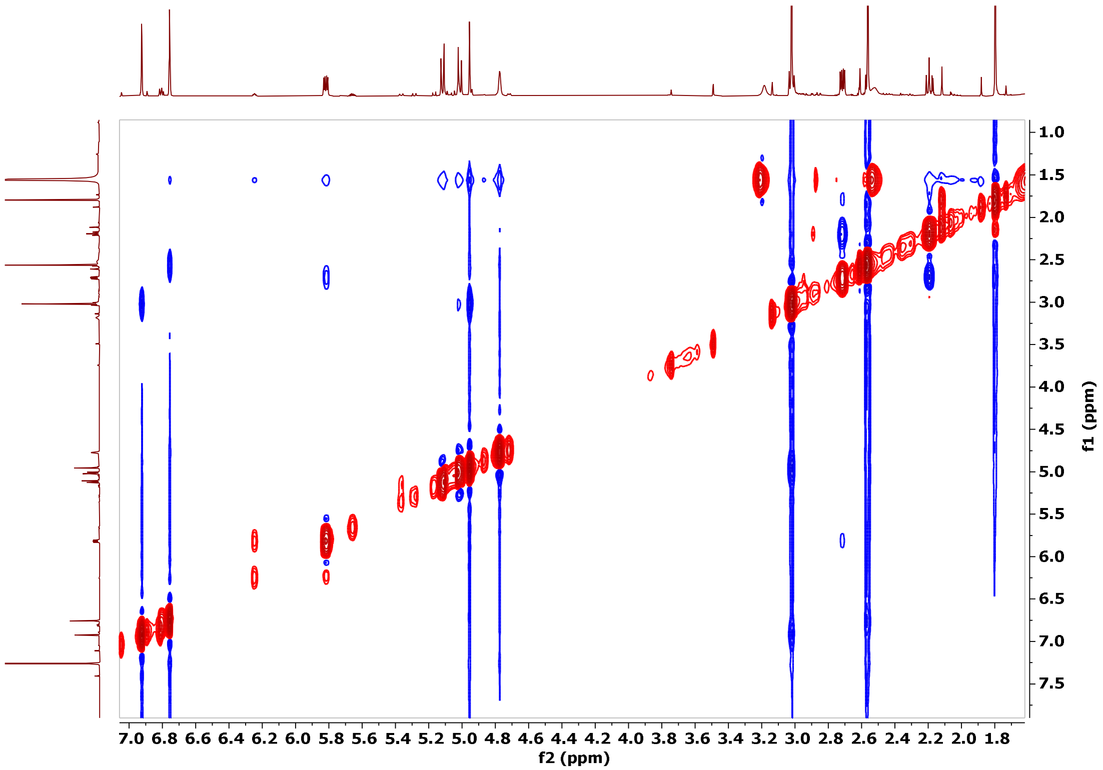

Figure S53. NOESY NMR spectrum of **38b** recorded in $CDCl_3$ at 700 MHz.

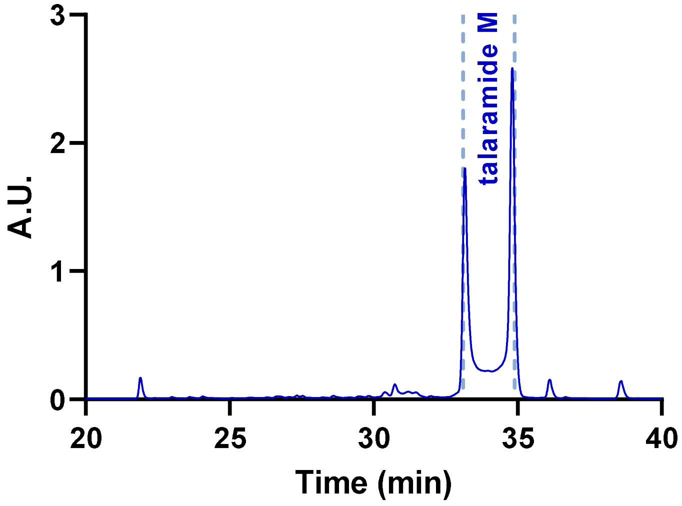

Figure S54. Chromatographic profile of talaramide M recorded at 254 nm, showing the co-existence of both, *P* and *M* atropoisomers.

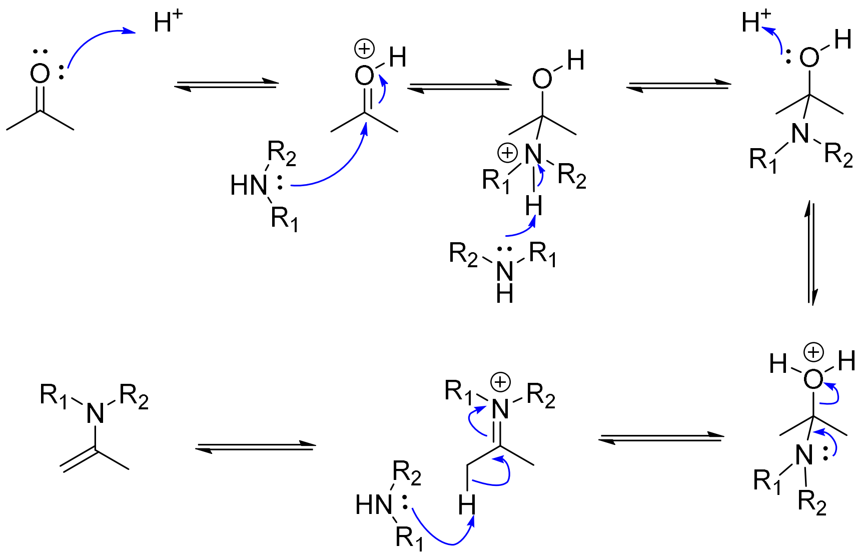

Scheme S1. General mechanism for the formation of enamines from ketones.
